# Reconstruction of the socio-semantic dynamics of political activist Twitter networks—Method and application to the 2017 French presidential election

**DOI:** 10.1371/journal.pone.0201879

**Published:** 2018-09-19

**Authors:** Noé Gaumont, Maziyar Panahi, David Chavalarias

**Affiliations:** 1 Centre d’Analyse et de Mathématique Sociales (CAMS), Centre National de la Recherche Scientifique (CNRS) / École des Hautes Études (EHESS), Paris, France; 2 Institut des Systèmes Complexes Paris Île-de-France (ISC-PIF), CNRS, Paris, France; Universidade de Lisboa, PORTUGAL

## Abstract

**Background:**

Digital spaces, and in particular social networking sites, are becoming increasingly present and influential in the functioning of our democracies. In this paper, we propose an integrated methodology for the data collection, the reconstruction, the analysis and the visualization of the development of a country’s political landscape from Twitter data.

**Method:**

The proposed method relies solely on the interactions between Twitter accounts and is independent of the characteristics of the shared contents such as the language of the tweets. We validate our methodology on a case study on the 2017 French presidential election (60 million Twitter exchanges between more than 2.4 million users) via two independent methods: the comparison between our automated political categorization and a human categorization based on the evaluation of a sample of 5000 profiles descriptions; the correspondence between the reconfigurations detected in the reconstructed political landscape and key political events reported in the media. This latter validation demonstrated the ability of our approach to accurately reflect the reconfigurations at play in the off-line political scene.

**Results:**

We built on this reconstruction to give insights into the opinion dynamics and the reconfigurations of political communities at play during a presidential election. First, we propose a quantitative description and analysis of the political engagement of members of political communities. Second, we analyze the impact of political communities on information diffusion and in particular on their role in the fake news phenomena. We measure a differential *echo chamber* effect on the different types of political news (fake news, debunks, standard news) caused by the community structure and emphasize the importance of addressing the meso-structures of political networks in understanding the fake news phenomena.

**Conclusions:**

Giving access to an intermediate level, between sociological surveys in the field and large statistical studies (such as those conducted by national or international organizations) we demonstrate that social networks data make it possible to qualify and quantify the activity of political communities in a multi-polar political environment; as well as their temporal evolution and reconfiguration, their structure, their alliance strategies and their semantic particularities during a presidential campaign through the analysis of their digital traces. We conclude this paper with a comment on the political and ethical implications of the use of social networks data in politics. We stress the importance of developing social macroscopes that will enable citizens to better understand how they collectively make society and propose as example the “*Politoscope*”, a macroscope that delivers some of our results in an interactive way.

## Introduction

Digital spaces are becoming increasingly present and influential in the functioning of our democracies, to the point where events transpiring in these spaces can no longer be ignored when it comes to analyzing trends in the political environment. In the wake of each of the major political upheavals that have shaken western democracies in recent months (e.g. Brexit, the election of Trump), commentators have turned to the social networking sites in the search for a better understanding of these events.

In the space of a few years, the use of social networking sites has become fully integrated into the strategies of political parties. Barack Obama was qualified as a “game changer” in 2012, when he became the first presidential candidate to make massive use of social networking sites for his campaign. The 2016 presidential campaign in the USA confirmed this trend, with an estimated total budget of around 500M € devoted to the use of social networking sites [1].

France followed the same trend. Nowadays, the largest political parties call on the services of specialized companies, in order to gain a better understanding of their potential voters, through the analysis of very large volumes of data provided by social networking sites [2]. Among these, Twitter is very well ranked among accessible sources of information. Moreover, it is the social networking site used by the majority of political figures, in response to current affairs or to provide visibility for their opinions and public statements. This social networking site has thus become an unavoidable medium for the political world, when it comes to delivering messages or establishing legitimacy.

With the objective of evaluating the potential of platforms like Twitter for research on social dynamics, and particularly in political sciences, we propose a method in the framework of complex networks analysis allowing the political communities and their evolution in a multi-polar political environment to be reconstructed and analyzed.

As a case study, we implement and evaluate this method on data we have collected during the 2017 French presidential elections that feature almost 60 million Twitter exchanges between more than 2.4 million users (*Politoscope* project—https://politoscope.org). The unusual hectic nature of these elections (see Text A in S1 File for a summary) represents a worse case scenario for this kind of reconstruction and guaranties the robustness of our approach. We then present some perspectives this type of reconstruction brings to social sciences and discuss its relevance for political sciences.

## Related work

A considerable volume of academic literature has been devoted to Twitter, as this platform benefits from two different advantages. Its content is massively duplicated by the traditional press or television and it is accessible to the general public, even though it may not be representative of the global or even France’s population [3]. Furthermore, data collection is relatively straightforward, thanks to various APIs proposed by Twitter (https://dev.twitter.com/overview/api).

Numerous methodologies can be used to access Twitter productions, in the context of the study of political activities. Some specific methods [4–6] are designed to take advantage of the content of tweets to infer the political orientation of a tweet or user. However, the constraints associated with tweet formats appear to limit the efficiency of these methods. For example, according to Cohen and Ruths [7], the hashtags used to discuss policy do not discriminate against types of political activism for people with low levels of activism.

In an effort to avoid the problems associated with automatic language processing, research has been channeled towards methods that analyze the structure, rather than the content, of exchanges made on Twitter. They have thus been inspired by a pioneering study of political blogs made in the USA [8], which has shown that there are more links between two blogs if they express the same political views.

In the framework of Twitter, there are several possible definitions for the relationship between two accounts. An account can follow or mention another account. It can answer, cite, comment or simply relay (retweet) a tweet from another account. All of these notions of a ‘link’ are significant, and depend on the objectives of the study [9]. However, in order to rank accounts according to their political orientation, it appears that the use of retweets is the most commonly used definition for a link between two accounts. In particular, Garimella *et al*. [10] and Connover *et al*. [6] have observed that in order to study political debates on Twitter, the use of retweets is more relevant than Twitter mentions.

Connover *et al*. [6] were among the first to take advantage of retweets in a study of the structure of the political environment in the USA. They retrieved its bipartisan nature, and showed that there are significantly less retweets between two accounts when they belong to different parties. Subsequently, numerous other studies have made use of this type of relationship. Boutet *et al*. [11] (resp. Larsson and Moe [12]) applied the same method to study the structure of the political space in England (resp. Sweden), and to analyze differences in practice, in terms of the manner in which political groups use Twitter mentions, hashtags and Internet links.

Garimella *et al*. [10] and Morales *et al*. [13] used the notion of retweets to measure the existence of polarization and controversies on Twitter. Amor *et al*. [14] used this to characterize the debate on *care.data* of the English National Health System and Guerrero *et al*. [15] also used this approach to study the *Catalan Process Toward Independence*.

These techniques have made it possible to retrieve bipolar political structures, although the notion of retweets also makes it possible to retrieve multi-polar structures. This was the case with Cherepnalkoski *et al*. [16, 17], who used retweets to determine the political affiliation of the Members of the European Parliament (MEP). However, these authors note that it is difficult to generalize this method, because in addition to their political connections, MEPs also have national preferences.

The retweet is not the only usable relationship that can characterize the structure of a political environment. The research of Barberá [18] made use of follower/followee relationships between accounts. The originality of their research is that of assigning a position, between -1 and +1 on a left-right ideological axis, to each account. Thus, an account is not assigned to the Democrats (resp. Republicans) party, but to a position close to other members of the Democrats (resp. Republicans). This approach makes it possible to compare a set of accounts over an ideological continuum. These results were successfully reproduced using Twitter data in France by Briatte and Gallic [19].

Using their ideological estimation technique, Barberá *et al*. [20] have also shown that there is a strong degree of retweet polarization when a political subject is mentioned, i.e. that there is a high number of retweets between ideologically close, and a low number between ideologically distant, accounts.

Finally, it is important to note that despite the publication of numerous studies evaluating ideological alignments, one of the aims of political science, *i.e*. that of predicting voting intentions, remains problematic [21], in particular as a consequence of the imperfect representativeness of Twitter with respect to the real population.

In just a few years, Twitter has thus become a productive research domain for social sciences. Although it has certain limitations in terms of the types of population that can be correctly studied, it is noteworthy that there have been a huge increase of Twitter use in the last few years with more than 15M active users in France in 2017 [22] (France population in 2017 was 66.9M) and an estimated number of active users that has tripled worldwide since the first studies in 2011 [23]. Consequently, it can be expected that some limitations that have been noted in previous papers might now be mitigated and new results could be obtained.

In the present paper, we reappraise the aforementioned retweet analysis techniques and we improve them to handle temporal and multipolar analyses, by applying them to a corpus generated by political activists over a period of nearly one year. We also combine two Twitter scrapping techniques (following accounts and following keywords) that have usually been used separately, leading to a Twitter corpora of very high quality.

The extend of the tweets collection and the prolonged period of study combined with an improved methodology and new analytics have, for one of the first times, made it possible to study the *socio-semantic dynamics* of a multi-polar political environment.

## Materials and methods

### Data

We used the CNRS Multivac platform (http://multivac.iscpif.fr), a platform dedicated to stream data capture and analysis, to capture in real-time the political twittosphere activity in France. For a period of 11 months prior to the presidential election, we continuously collected almost 60 million tweets concerning more than three thousands of the key French political figures and few dozens political keywords, that were used by more than 2.4 million individual accounts (see Text B in S1 File). We refer to this set of data as D.

All data was collected according to Twitter’s terms of service and privacy conditions and are made available according to the terms of use of Twitter [24]. All anonymized data derived from the analysis of this database in the context of this paper are available for download [25].

We estimated the proportion of tweets in D sent by bots to be less than 5% (see Text C in S1 File), thus ensuring that our results remain representative of online political activism.

Retrospectively, given the unusual hectic nature of these elections (see Text A of S1 File for a summary) that took place in a multi-polar political environment, this dataset was found to be both of great interest to understand the French political landscape and of high value to challenge community reconstruction methods.

### Identification of political communities

#### Definition of social groups

Social groups, whatever their definition, have blurry and shifting boundaries. Our approach is not focused on finding the exact location of social groups boundaries, but rather the way in which they shift with time, the forking they can reveal, the subcorpus of tweets that may be established through the estimation of social groups affiliations and the diffusion processes it allows to study.

The methodology we propose for the identification of political social groups does not assume that these groups can be determined accurately or exhaustively. The method takes on board qualification inaccuracies at the level of the individual (for example, a some errors in the determination of some political affiliations), and the arbitrary nature of certain choices related to the positioning of boundaries between these groups (for example the choice of parameters values for reconstruction algorithms).

One definition of social group from a sociological perspective is particularly adapted to Twitter studies. In 1890, Gabriel Tarde [26] defined a *social group* as “*a collection of beings as they are imitating each other or as, without imitating each other at present, they resemble each other and their common traits are ancient copies of the same model*” (“*Une collection d’êtres en tant qu’ils sont en train de s’imiter entre eux ou en tant que, sans s’imiter actuellement, ils se ressemblent et que leurs traits communs sont des copies anciennes d’un même modèle*”). The notion of imitation invoked by Tarde was relatively general, and covers a high proportion of broader notions related to *social learning* or *social influence* (see [27, 28] for the conceptualization of these ideas) and in particular, all cases where an attitude, a way of doing or expressing oneself, or an opinion is transmitted with few modifications between two people.

Twitter offers an ideal terrain for the adaptation of this definition to digital information space, by analyzing the way people express themselves on this platform. In fact, Twitter combines three key elements, allowing the propagation cultural traits to be monitored:

- Everyone can publish, without filtering, content that is visible to all other users,
- The follower/followee system allows anyone to subscribe to the publications of any other user,
- The retweet system makes it possible to effortlessly copy the content produced by an other person (retweet) by forwarding an identical copy of that content to all of his/her followers.

By concentrating on retweet events (relayed, unmodified information), we can establish markers allowing to define, for a given period, a set of user groups within which the Twitter contents tend to circulate whilst being affected by modifications that are less significant (exact copies), than when they circulate between such groups (transitions during which tweets can be distorted, ironically or humorously commented, contradicted or simply not copied). That being said, we considered the following definition:

***[Definition] Social groups in the political twittosphere***: *dense networks of accounts that recurrently relay content, without modification (retweet), between each other*.

#### The retweet communities

In order to define user groups based on retweets, we selected the retweets from our dataset D and defined a graph *G*_*T*_ = (*V*, *E*) in which each node, *u* ∈ *V*, is a Twitter account and each (*u*, *v*, *w*) ∈ *E* is a weighted and undirected link between nodes *u* and nodes *v*, *w* being proportional to the number of retweets having occurred between accounts *u* and *v* during a period of time *T*.

To detect political groups in *G*_*T*_, we adopt an approach similar to those proposed by [29–31] by applying a non-overlapping community detection algorithm to *G*_*T*_. From the set of existing and regularly cited state-of-the-art algorithms [32–34], we apply the Louvain algorithm developed by Blondel *et al*. [35] which, in view of the definition adopted for the notion of a link, computes node communities that correspond to our interpretation for the social groups we are looking for: these are subgroups of accounts, which exchange much more information without modification within the group than with the rest of the network.

Thereafter, we adopt the following definitions:

***[Definition] Link w-strong***: *two accounts A and B are related by a link w-strong over the T period if they have retweeted each other at least w times, in either direction*.
***[Definition] w-communities over period T***: *communities detected with the Louvain algorithm on the retweet graphs, on which only those links at least as strong as w-strong have been retained*.

**Fig 1:**
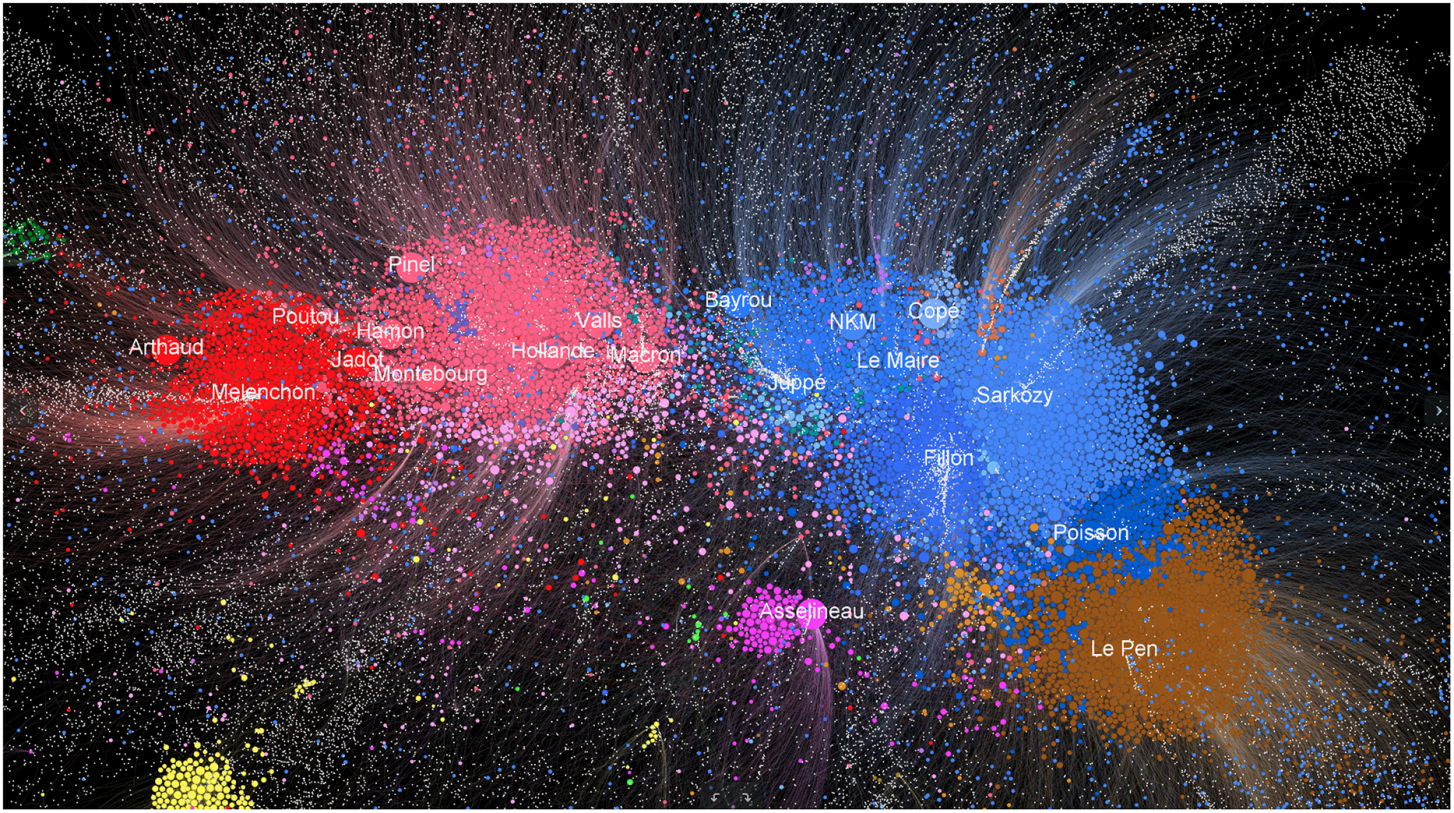
Graph of the 5–communities of the French pre-electoral political twittosphere calculated from August 1st to December 31th 2016. The multi-polar political landscape is clearly visible with a position on a left-right axis for most political parties. It should be noted that the alliances observed during the political campaign took place between political personalities that were close on this map: Hamon-Jadot, Macron-Bayrou, Le Pen—Dupont-Aignan. The right-wing (*Republicans*, blue) is divided into several currents which are reflected by different communities. The Juppé community is at the left of the right-wing while the Fillon and Sarkozy communities are close from each other and much more at the right. The latter two merged after the right-wing primary while the Juppé community gradually migrated to the Macron community. The *Socialist Party* was also made up of a “left-wing” (Hamon community, Montebourg and Pinel community) and a “right-wing” (Holland, Valls). This graph has been spatialized with the Gephi software [36] using the ForceAtlas2 algorithm [37]. See File A1 at DOI: 10.7910/DVN/AOGUIA for the anonymized gexf version of this graph.

The political communities found over a period *T* according to this definition are noted $C_{w}^{T}={\left\{C_{i}^{T}\right\}}_{1\leq i\leq N}$. In the analyses and visualizations, they are labeled with the names of the main political figures present in these groups, in an effort to provide a view of the development of political communities with respect to the key personalities in the French presidential campaign (cf. Fig 1 for an example).

In the following, we have taken labels from the list of presidential candidates, and added a small number of political figures who, although they were not candidates, played an important role in this campaign (e.g. outgoing President, former President, preferred personalities in the polls, etc., see S 2.3 Text). The resolution used with the Louvain algorithm was its default value.

A significant portion of accounts do not belong to any community with this approach. They correspond to accounts that, although dealing with political issues, do not fit into the informational sphere of a group of political activists. We will refer to this set of account as “*the sea*”.

#### Temporal and social granularity

It is possible to study the political environment coarsely or finely, by selecting a shorter or longer list of representative accounts, and by adjusting the resolution of the Louvain algorithm in order to identify distinct communities in the neighbourhood of these accounts. We observed that the different political currents within a party could be identified in this way. Such an analysis lies however beyond the scope of the present study and we choose to keep the default Louvain algorithm settings that made it possible to identify the major political parties and currents accurately.

Socio-semantic networks evolve together: the actors are influenced by the content they are exposed to, which leads them to develop their local network, and thus to modify the nature of the content they will be exposed to in the future. As a result, it is common for individuals to change their sphere of political influence during a presidential campaign, or to be influenced by new political figures, which leads to a modification of the Twitter patterns.

In order to take this phenomenon into account, we consider sets of graphs $G={\left\{G_{T}\right\}}_{T\in T}$ where T is a set of time intervals, $T={\left\{T_{i}\right\}}_{1\leq i\leq K}$, such that $T_{i}=\left[t_{min}^{i},t_{max}^{i}\right]$, with $t_{min}^{i}$ and $t_{max}^{i}$ being the positive integers representing dates in the Unix reference system (to which we refer using the conventional date). The links in a graph $G_{T_{i}}$ are defined only by retweets that occur within the time interval *T*_*i*_.

The choice of T is important: if the intervals are too long (several months), it will not be possible to follow the social groups reconfigurations as a result of excessive temporal aggregation. On the other hand, if the intervals are too short (e.g. 1 day), the corresponding graphs will be almost empty and have no statistical significance. It is thus important to find a suitable compromise between the temporal adaptability of the political groups reconstruction method and its stability.

As a consequence of variations in political opinion, resulting from the overlapping of several dynamic processes with highly different time constants (e.g. reconfiguration of political currents inside a party, reconfiguration of parties themselves, development of the European political scene, etc.), it may be relevant to analyze the data using several different time scales.

For example, the analysis of the 5–communities over the 5 months period August 1st to December 31 2016 (cf. Fig 1) depicts a quite accurate picture of the pre-electoral political landscape. Its relevance was confirmed by the subsequent alliances that took place in 2017 during the campaign while respecting the personalities’ proximities within this map: Hamon with Jadot, Macron with Bayrou, Sarkozy with Fillon, Le Pen with Dupont-Aignan.

For the present study, we chose a time scale matching timing of Twitter and the media in the context of a political campaign. By analyzing the temporal distribution of retweets within our corpus, we observed that both current political events and Twitter activity have a weekly periodicity, and that on average the retweet period of an original tweet is of the order of one week, with 98.13% of retweets being made within a period of seven days (see Text D in S1 File).

We thus considered seven-days intervals to be a characteristic period for political activity on Twitter, and used two-week intervals *T*_*i*_ in order to obtain overlapping periods in the temporal reconstruction (see below). In the following analyses, we consider the communities computed every day over a 14 days period to assign a community to each tweet of that day. For the analysis of the dynamics of the communities, we took the subset of the communities computed every Monday, with $G={\left\{G_{T}\right\}}_{T\in T}$ where T is a set of temporal intervals, such that ∀*k* ∈ [2, *K* − 1], |*T*_*k*_| = 14 *days*, |*T*_*k*−1_ ∩ *T*_*k*_| = |*T*_*k*_ ∩ *T*_*k*+1_| = 7 *days*, when the intervals are arranged according to their starting date.

Each tweet in our dataset is thus taken into account in exactly two retweet graphs for the analysis of community reconfigurations. As shown below, this choice allows transformations within political communities and their semantic space to be followed in a reasonably continuous manner.

**Fig 2:**
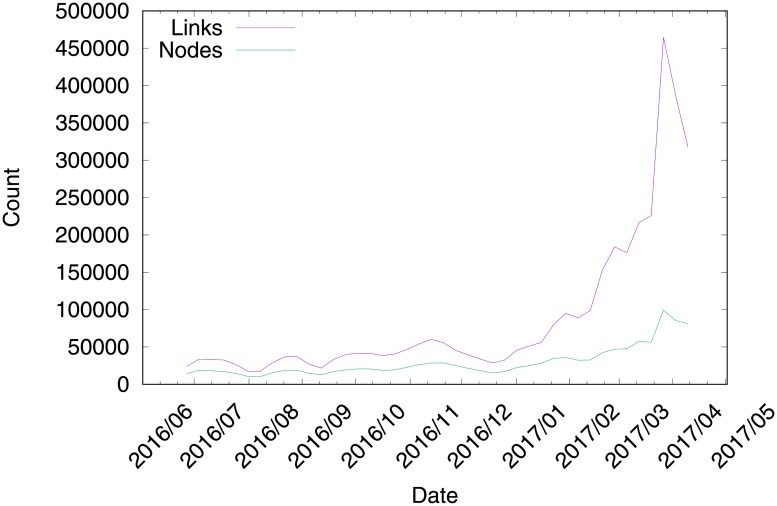
Variation in the number of nodes and links for the graphs of $G_{T_{i}}$.

To set the value of *w* in the community definition, we choose to retain on average the strongest 10% retweet relationships over *T* for the analysis, which led us to conduct our analysis on the 3–communities. The change in the number of links and accounts of these 3–communities over time is shown in Fig 2.

#### Detailed analyses

Not all candidates have the same communication strategy and some use relatively little Twitter. We respect this choice and we have decided to present detailed statistics only for the communities which leaders have a significant presence on Twitter.

Computing the number of tweets of each candidates on March 31, 2017, we observed a gap between the six most active candidates that tweeted more than 500 times and the others. Consequently, we retain these six communities for detailed investigations: Asselineau, Fillon, Hamon, Le Pen, Macron and Mélenchon. Among them, Asselineau was ranked in the “small” candidates (0.92% of the votes at the first round). The other five represent the main political trends in France: very left (Melenchon), left (Hamon), center liberal (Macron), right (Fillon), extreme right (Le Pen).

All 5 other candidates received less than 1.5% of the votes at the first round except for Dupont-Aignan who received 4.7%. The total of their weight at the first round was 7.82% of the votes.

#### Remarks on the use of retweets

On Twitter, markers of social influence related to forms of expression or opinions extend far beyond the framework of retweets. Individuals can for example influence one another in their use of expressions or hashtags, without necessarily having to retweet. Furthermore, the act of retweeting can be interpreted in several different ways, with for example a negative interpretation being common when the retweet is delayed (see Text J in S1 File). However, in the vast majority of cases, when retweeting is repeated in a political context, the retweet contributes to the propagation of ideas and opinions across the network [38–41], and can be considered as a form of agreement with the ideas of a candidate or a political party [42].

Account following is another well-studied functionality on Twitter, which could also be used as a proxy to identify some form of social influence. It is however rather uncommon for a user to stop following an account, such that from this perspective the “follow” action is less informative than the retweet, which is intrinsically temporal and indicates a user’s affinity with comments made on another account, at a given point in time. Furthermore, the “follow” function can be used for the simple purpose of keeping up with statements and comments made on another account, whilst at the same time maintaining a certain distance from that user.

Finally, retweeting has the advantage of quantifying the social influence present between two accounts since, for a given period, there may be no, few or many retweets between two accounts. In addition, these retweet relationships can be mono- or bi-directional, however this information is not considered in the present study.

### Characterization of the themes of the 2017 presidential campaign

To further characterize the political communities, that have been reconstructed solely from social interactions, we have analyzed their semantic profile. Our objective is not to characterize them in an exhaustive manner, but to provide a preliminary overview of how communities differ in their political agenda and framing of the societal issues.

For this purpose, we have carried out an analysis of the political discourse in order to extract the main debates of this campaign. This was done using two types of sources: policy measures reported in campaign programs, written in developed language, and candidate tweets, written in frequently spoken language.

For the analysis of candidates’ programs we first contacted, on 1 March 2017, all the campaign teams that were about to achieve to collect the 500 signatures from French mayors required to be able to become an official candidate (the Constitutional Council published the official list of candidates only on 20 March 2017). We invited them to provide us with their program elements and key expressions that they would consider characteristic of their program. Unsurprisingly, we had almost no response, only the team of a “small” candidate indicated to us that given the size of the campaign team, they were not sure they could participate. We used then the programs of the eleven candidates downloaded from their websites (between February 14 and March 24, 2017) from which we manually extracted 980 policy measures (see File A2 at DOI: 10.7910/DVN/AOGUIA for an editable version of these policy measures).

To complement this corpus, we extracted from the *Politoscope* database 25, 883 tweets published by the eleven candidates and few other key political figures between August 2016 and January 2017 (see Text B in S1 File). This second corpus has the advantage of highlighting the themes that emerged during the political debates, independently of the candidates’ programmatic orientations.

There are two categories of mainstream methods for the extraction of topics from unstructured text: co-word analysis [43] and topic modeling with LDA like methods [44]. In these approaches, topics are defined as “bags of words”, inferred from the statistics of appearance of a list of predefined keywords the documents. This list is itself obtained through more or less advanced text-mining methods within the fields of natural language processing (NLP) and machine learning.

It has been demonstrated that LDA has some limitations on analyzing short documents [45] or corpora of small size [46], which are two constraints present in our Twitter corpora (short text messages) and political measures corpora (less than 1000 documents).

Consequently, we analyzed these two corpora using the CNRS text-mining software *Gargantext* (https://gargantext.org; open source at https://github.com/ISCPIF/gargantext), that implements advanced NLP methods and co-word topic detection; as well as visual analytics methods for the representation and interaction with the results.

In the first few steps, *Gargantext* uses a combination of lemmatization, post-tagging and statistical analysis like tf-idf [47] and genericity/specificity analysis [48] to identify from the text-mining few thousand groups of keywords that are specific to the political discourse. These keywords were further screened by the authors in order to select the most meaningful ones (*i.e*. stop words or poorly formed expressions that would have passed the text-mining steps have been removed, important hashtags or neologisms from Twitter like *frexit* have been added). Last, we carefully read all the political measures with the selected keywords highlighted in the text in order to check that no important keyword was missing. This led to a vocabulary of nearly 1600 groups of keywords qualifying the themes of the presidential campaign (see Text I in S1 File for the list of keywords).

We used the *confidence* proximity measure to assess the thematic proximity between the selected terms. The *confidence* measure is the maximum between two conditional probabilities. If *P*(*x*|*y*) is the probability that a document mentions term *x* knowing that it already mentions term *y*, the *confidence* is defined by *max*(*P*(*x*|*y*), *P*(*y*|*x*)). It has been demonstrated to be one of the best choices to automatically induce general-specific noun relations from web corpora frequency counts [49].

**Fig 3:**
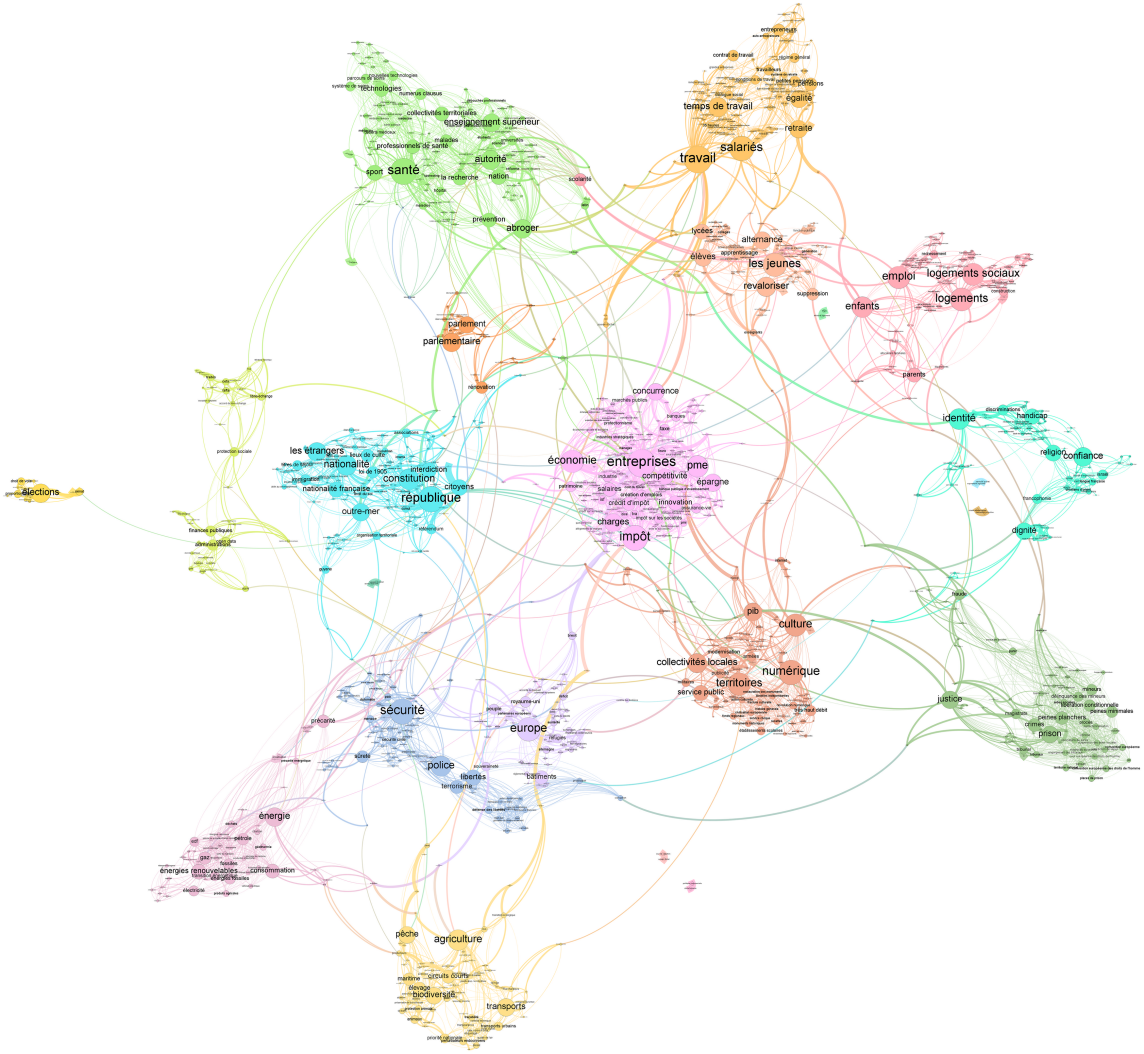
Map of the themes covered in the 2017 presidential programs, drawn-up using *Gargantext*. The map has been built from policy measures extracted from the candidates’ programs. The nodes of the map are labels for groups of terms deemed equivalent in political discourse. The link between a label *A* and a label *B* indicates that the probability that *A* and *B* are jointly mobilized in the same political measure is high. *Gargantext* applies the Louvain algorithm to identify clusters of labels with strong interaction between them and displays them in the same color. To improve readability, the map was edited in the Gephi software (https://gephi.org) to set the size of nodes and labels according to a monotonous function of their PageRank [50]. File A3 at DOI: 10.7910/DVN/AOGUIA provides an editable version of this map (gexf).

We applied the Louvain algorithm [35] to identify groups of terms delineating topics. Last, we generated the topic map for each of these two corpora (cf. Fig 3 for the map from the 2017 presidential programs). All these processing steps are part of the *Gargantext* workflow.

We relied on these maps to select eleven topics that we identified as particularly important and representative of the debates.

### Validation analysis

In order to validate our reconstruction method, we have manually verified the political categorization on Monday 6 February (communities computed over the activity period Monday 23 January—Sunday 5 February 2017) for all active followed accounts (2,440) and a sample of 2,500 active random accounts that day. This period corresponds to the end of the primary of the right, before any changes in the political landscape due to some alliances between candidates (ecologists/Jadot with socialists/Hamon); center/Bayrou with En Marche/Macron, DLF/Dupont-Aignan with FN/Le Pen).

For these two samples, we have extracted the Twitter meta-data of the accounts and we have manually coded the accounts political affiliation each times there was a clear support or a declaration of party membership in the account profile.

We associated all the main political leaders with a reference party using the correspondence in Table 1.

**Table 1:** Mapping between political leaders and their party on Feb. 6 2017.

Leader	Party
Nathalie Arthaud	LO
François Asselineau	UPR
François Bayrou	MODEM
Jacques Cheminade	S&P
Nicolas Dupont-Aignan	DLF
Francois Fillon	LR
Benoit Hamon	PS
François Hollande	PS
Yves Jadot	EELV
Alain Juppe	LR
Marine Le Pen	FN
Emmanuel Macron	EM
Jean-Luc Melenchon	FI
Philippe Poutou	NPA
Nicolas Sarkozy	LR
Manuel Valls	PS

In the account descriptions, some account holders were referring to parties that hadn’t any candidates running for this election. We discarded the mentions that correspond to parties that had not clearly rallied any running candidate (PCF and Parti Progressiste among the most mentionned). We considered as equivalent to *France Insoumise* (FI) all the parties that were in the former Mélenchon’s *Front de Gauche* coalition (before he decided to run for the presidency and created the *France Insoumise*). Given the official declarations of the UDI party [51] we also considered the UDI (*Union des Démocrates Indépendants)* as supporting *Les Républicains* (LR) as of Feb. 06 2018, although this position it was clearly not unanimous since few weeks later, a dozen UDI elected officials rallied Macron [52].

This manual analysis resulted in two distinct lists of 885 followed accounts and 414 random accounts respectively that were publicly supporting one of the 11 running candidates at the time of the community reconstruction (other accounts in the samples did not give enough information in their profile to identify political affiliation).

We then compared the automatic categorization made on that period with this manual coding to assess the precision and recall of our algorithm. We limited this comparison to accounts that have made at least 10 tweets over the 14 days period of observation.

Although we could expect a selection bias due to the fact that the most politically engaged people are both more likely to declare a political affiliation in their Twitter profile and are more easily categorized through automated methods, we consider this as a reasonable limitation to the precision of our estimations of our reconstruction accuracy. It should also be emphasized that there is no ground truth regarding political affiliations since at any time, a significant proportion of the population is undecided or is changing its opinion. There will thus always be an intrinsinc uncertainty in the categorisation of accounts whatever the methodology.

To complement this validation, we compared the key reconfigurations highlighted in the landscape reconstruction with major news events (see the Results section).

## Results

### Precision and recall of the political landscape reconstruction

The comparison between the two categorizations revealed a very good fit between the automatic categorization and the manual coding of political affiliations: a precision above 93% for both samples and a recall above 63% for both samples.

Tables 2, 3, 4 and 5 show the recall, the precision and the confidence intervals for each political party and sample.

**Table 2:** Recall for the followed accounts that were active on the period January 23–February 6 2017 with at least 10 tweets.

	Correctly retrieved	Manually tagged	Recall	Confidence Interval
LR	162	265	61.13%	[-5.99%, +5.67%]
PS	132	215	61.40%	[-6.65%, +6.25%]
FN	47	50	94.00%	[-10.22%, +3.94%]
FI	18	36	50.00%	[-15.53%, +15.52%]
MODEM	2	3	66.67%	[-45.90%, +27.18%]
EM	1	2	50.00%	[-40.55%, +40.55%]
Total	362	571	63.40%	[-4.03%, +3.85%]

**Table 3:** Precision for the followed accounts that were active on the period January 23–February 6 2017 with at least 10 tweets.

	Coherently tagged	Auto. tagged	Precision	Confidence Interval
LR	164	165	99.40%	[-2.75%, +0.50%]
PS	132	138	95.65%	[-4.81%, +2.34%]
FN	47	47	100.00%	[-7.56%, +0.00%]
FI	18	18	100.00%	[-17.59%, +0.00%]
EM	1.0	14	7.14%	5.87%, +24.32%]
DLF	0	1	0.00%	[0.00%, +79.35%]
EELV	0	4	0.00%	[0.00%, +48.98%]
Total	362	387	93.54%	[-2.90%, +2.04%]

**Table 4:** Recall for the random sample of accounts that were active on the period January 23–February 6 2017 with at least 10 tweets.

	Correctly retrieved	Manually tagged	Recall	Confidence Interval
LR	69	122	56.56%	[-8.86%, +8.46%]
PS	33	60	55.00%	[-12.51%, +11.91%]
FN	66	86	76.74%	[-9.95%, +7.67%]
FI	37	52	71.15%	[-13.43%, +10.52%]
EM	31	51	60.78%	[-13.70%, +12.19%]
UPR	7	8	87.50%	[-34.59%, +10.26%]
EELV	5	6	83.33%	[-39.68%, +13.66%]
S&P	1	1	100.00%	[-79.35%, +0.00%]
DLF	3	3	100.00%	[-56.15%, +0.00%]
MODEM	0	3	0.00%	[0.00%, +56.15%]
PP	0	1	0.00%	[0.00%, +79.35%]
Total	252	393	64.12%	[-4.86%, +4.58%]

**Table 5:** Precision the random sample of accounts that were active on the period January 23–February 6 2017 with at least 10 tweets.

	Coherently tagged	Auto. tagged	Precision	Confidence Interval
LR	69	71	97.18%	[-6.88%, +2.04%]
PS	33	39	84.62%	[-14.34%, +8.14%]
FN	66	66	100.00%	[-5.50%, +0.00%]
FI	37	39	94.87%	[-11.76%, +3.71%]
EM	31	32	96.88%	[-12.62%, +2.57%]
UPR	7	7	100.00%	[-35.43%, +0.00%]
EELV	5	5	100.00%	[-43.45%, +0.00%]
S&P	1	1	100.00%	[-79.35%, +0.00%]
DLF	3	4	75.00%	[-44.94%, +20.44%]
Total	252	264	94.55%	[-3.23%, +1.93%]

The precision and recall are similar between political parties and samples with two exceptions.

The FN (extreme right) has a higher recall for the followed accounts. This indicates that FN supporters express online their political opinions more strongly than any other supporters.

The EM party (Macon’s new party) has a very small precision for the followed sample (7.14% vs. an average of 93.54). This means that these accounts did not mention their support to EM in their profile but their on-line behavior could be analyzed as relaying pro-Macron information. Indeed, the list of followed accounts was based on the political figures elected before the creation of the *En Marche* movement. At the time of the sample capture (01/23-06/02 2018) Macron’s movement what at it’s beginning and was gaining momentum. It started recruiting support from other parties supporters but apparently not to the point that these supports would openly express their opinion in their Twitter profile.

### Evolution of political communities

Another external validation of our approach comes from its ability to follow with great temporal accuracy the changes in the French political landscape that have been reported in the media.

Opinions and political communities vary together. Political campaigns involve reversals, bombshells, and reconfigurations of political support. Commentators do their best to anticipate and analyze these changes, whereas the political parties strive to mask what is going on behind the scenes, in order to catch their adversaries off guard. The main result of our study is that the automatic analysis of retweet patterns is able to measure, sometimes before the media can make any announcement, the changes taking place inside political communities in response to these events and, in some cases, in response to anticipated events.

**Fig 4:**
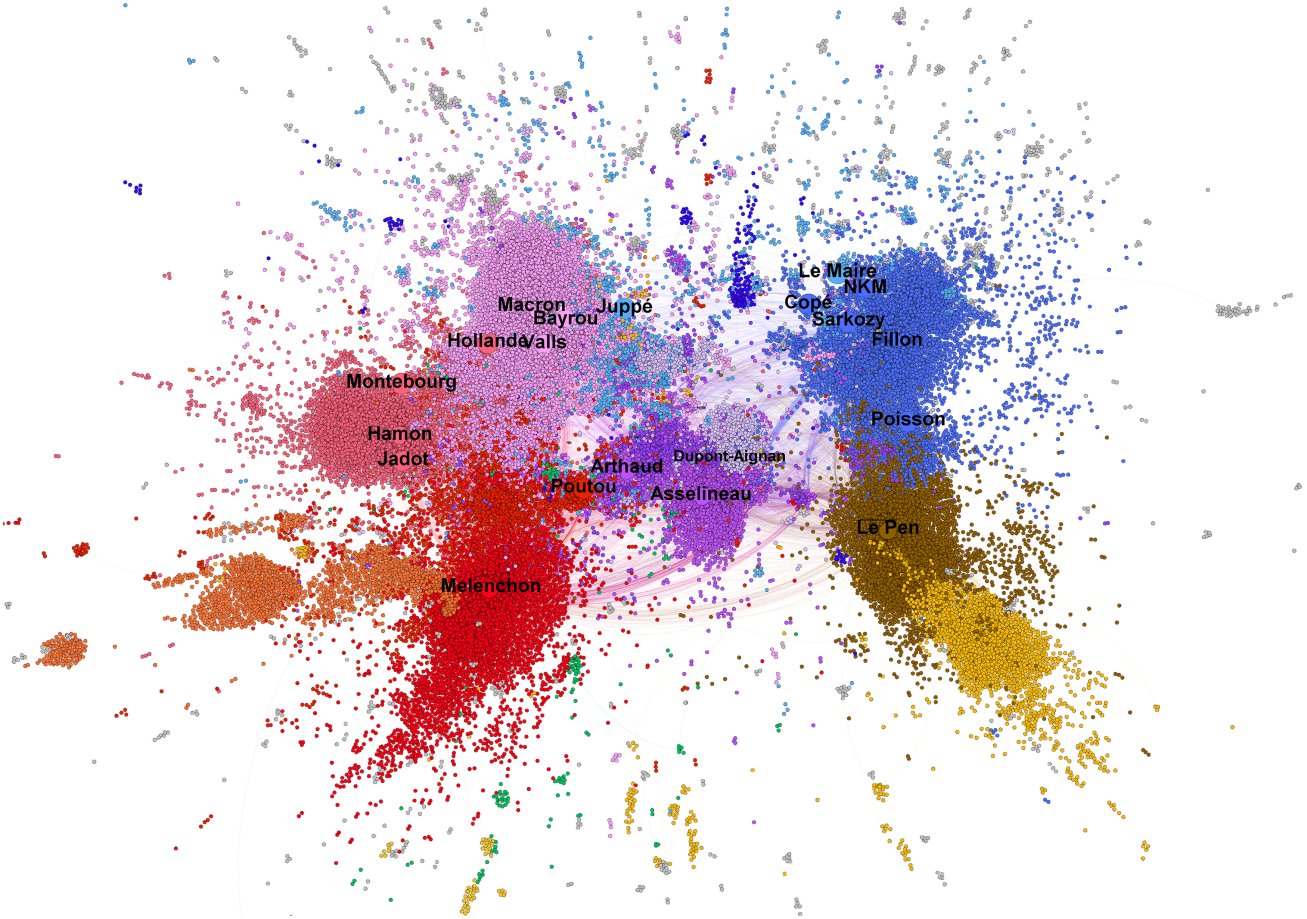
3-communities in the French political environment on the day of the first round of elections (T = [9 April 2017–23 April 2017]). The environment is highly fragmentary and multi-polar, and the main political forces are represented (the percentages shown correspond to the scores obtained in the first round): Mélenchon and *France Insoumise* (bright red—19.58%), Hamon and the *Parti Socialiste* (light red—6.36%), Macron and *En Marche!* (pink—24.01%), Fillon and *Les Républicains* (blue—20.01%), Le Pen and the *Front National* (brown—21.30%), Dupont-Aignan and *Debout la France* (lilac—4.7%). In the case of the “small” candidates, Asselineau (0.92%) and Arthaud (0.64%) are located inside the same community (purple), Poutou (1.09%) is positioned close to Mélenchon, but does not have a real Twitter community. It can be noted that not only is Bayrou located inside Macron’s community, which is not surprising in view of their unification on February 23, 2017, but also that Juppé, who was previously a candidate for the *Les Républicains* primary election, is considerably closer to the community of Macron than to that of Fillon and Sarkozy. His “lieutenant” Edouard Philippe, was later to become Macron’s Prime Minister. There are in addition two significant communities that are not labeled with a political leader. Colored orange, to the left of the figure and close to Mélenchon’s community, one can discern a community identified as being associated with the *Discorde Insoumise*, a video-game community which played an important role in Mélenchon’s campaign. Coloured yellow, at the bottom-right of the figure, close to Le Pen’s community, we identify a community of English-speaking populist and nationalist accounts which strongly supported Le Pen. This community is itself made up from sub-communities, in particular English-speaking supporters of Le Pen, Trump supporters, and UK pro-Brexit supporters. One can also observe significant differences with respect to the pre-presidential campaign of 2016 (Fig 1): in addition to the “left-right” axis, which was dominant at that time, a new (“vertical” on the map) axis appears to be significant, distinguishing between parties with a nationalist, patriotism or protectionist focus and those accepting or adhering to the globalization of economic trade. Note that the fact that the “small” candidates are located at the center of the map should not be interpreted as a form of centrality. Only topological adjacencies are significant on this type of graph. As the “small” candidate communities are not well connected with the other communities, they are located at the center as a consequence of the spatialization algorithm used to make the graph. File A4 at DOI: 10.7910/DVN/AOGUIA provides an editable version of this graph.

**Fig 5:**
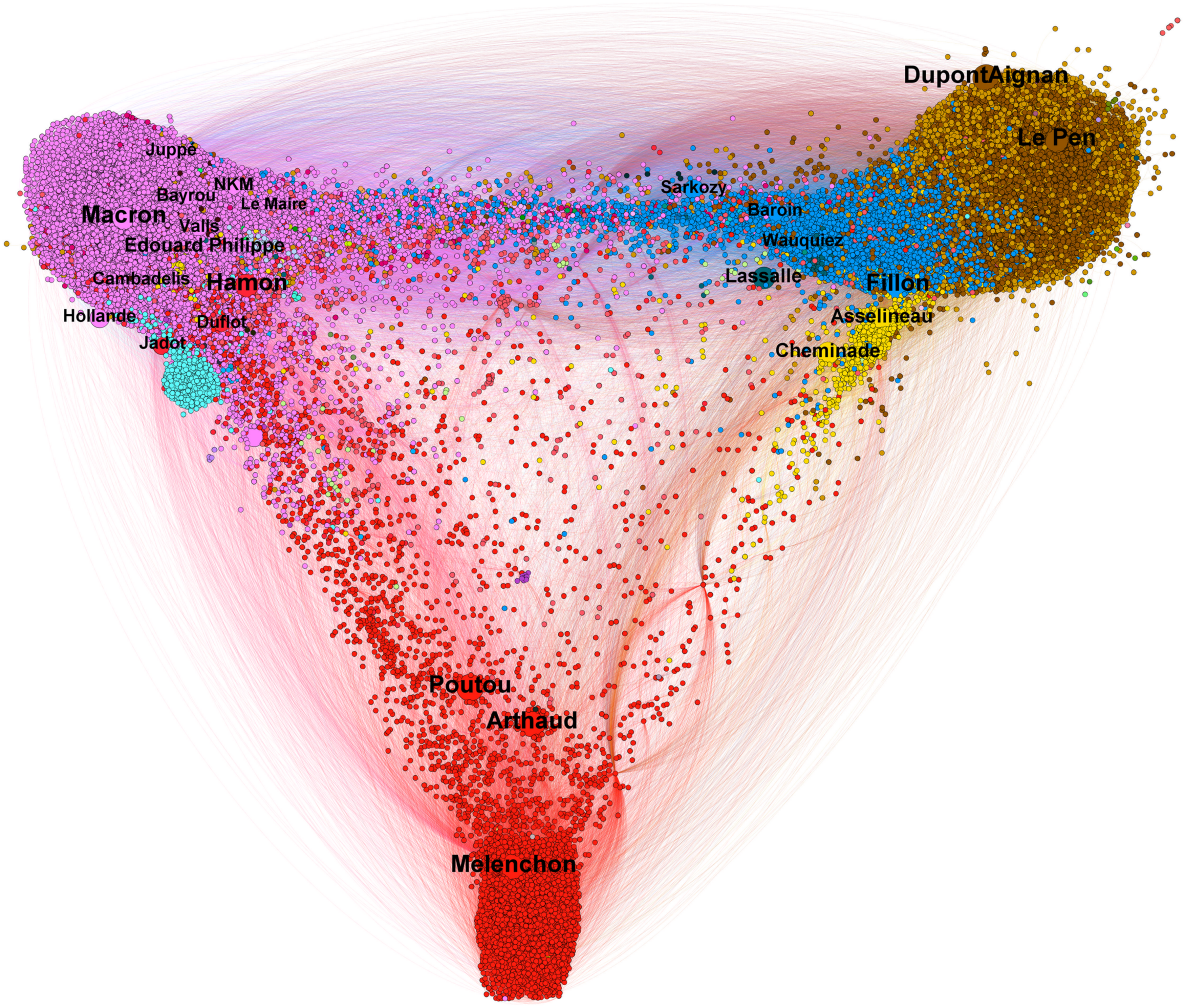
3-communities in the French political environment between the two rounds of elections (T = [April 27 2017–May 07 2017]). With only two candidates left running for the presidency (Macron and Le Pen), the political patterns of Twitter political activist changed radically with two poles corresponding to supporters of the two remaining candidates (that came from the recomposition of former communities) and a third pole constituted by those who refused to take sides. These latter positioned themselves to be the next opposition force to the future government. It should be noted that the party *Les Republicains* split during this between-two rounds, the tenors of this party being scattered between Macron and Le Pen informational spheres. File A5 at DOI: 10.7910/DVN/AOGUIA provides an editable version of this graph.

These changes can be of large magnitude in a very short period of time. For example, the comparison between the 3–communities political landscape of the two weeks before the first round (Fig 4) and the landscape of the two weeks between the first and second round (Fig 5) shows a complete reorganization of the communities, some being torn apart in the process.

In an effort to monitor political community reconfigurations over periods of time, when changes are perceptible, we compared the communities pairwise, over consecutive intervals of time. This approach allows transformations in the political communities to be followed without interruption.

**Fig 6:**
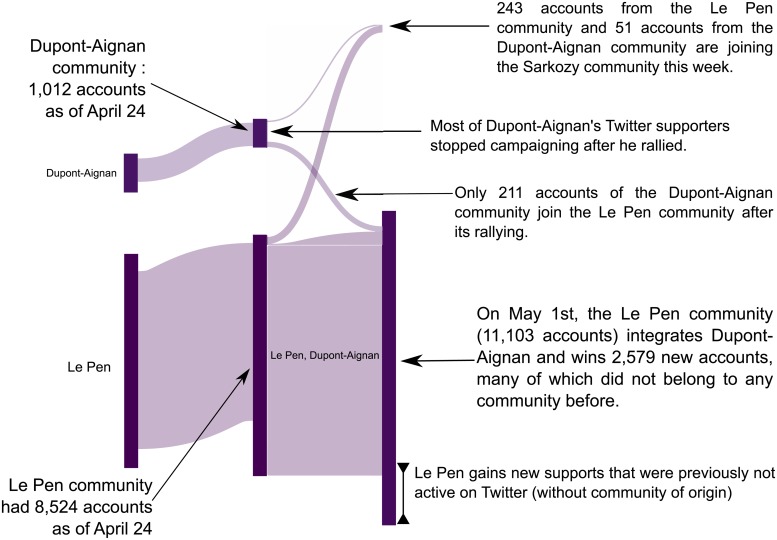
Representing communities evolution with an alluvial diagram.

**Fig 7:**
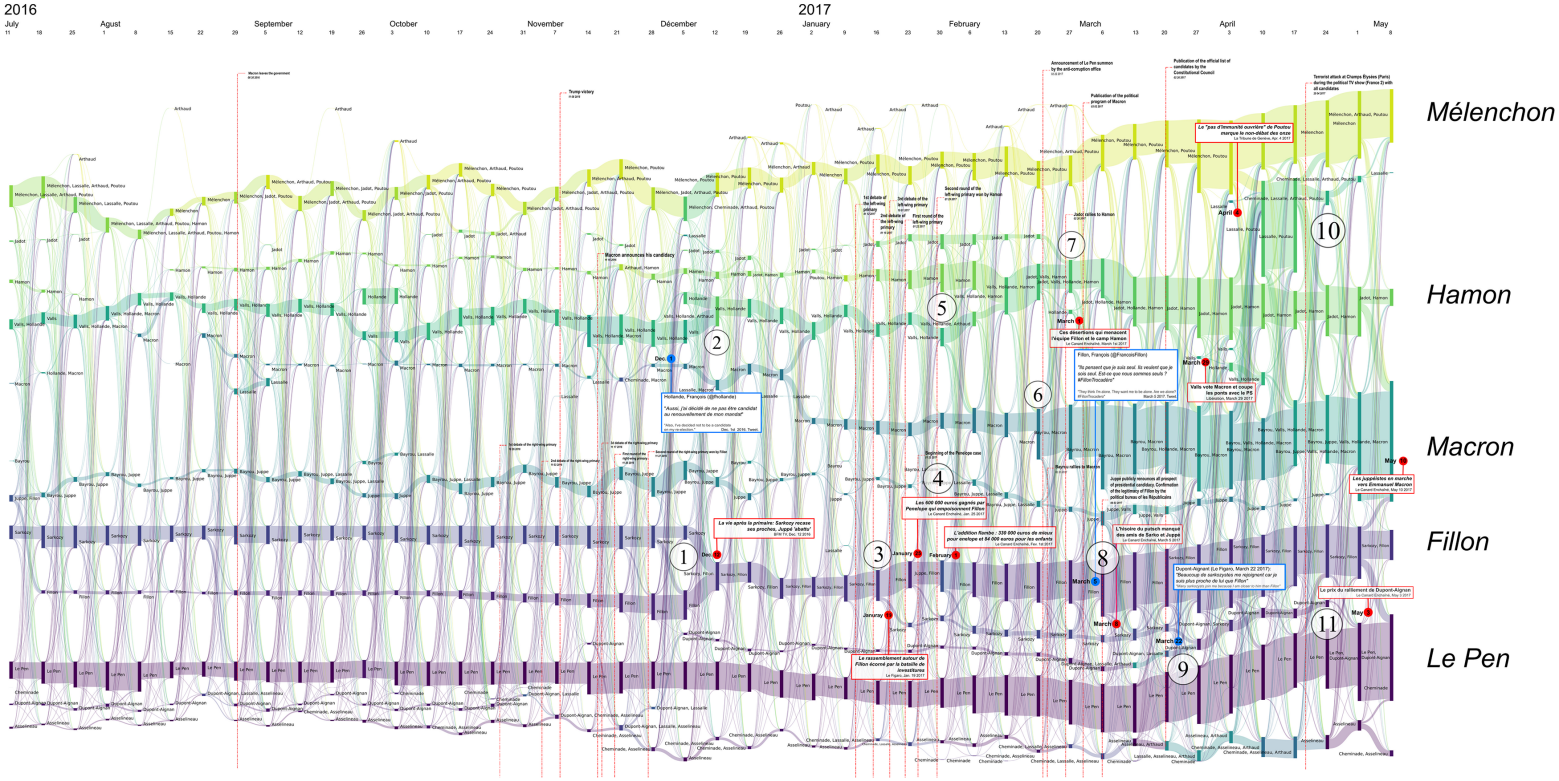
Reconfigurations of political communities between June 2016 and April 2017. Several major newspaper headlines have been added to shed light on the observed bifurcations. The labels of communities that were still active during the second round have been added in large characters to improve their readability. Each vertical bar corresponds to a political community on Twitter, labeled with the accounts of candidates who were active in the community. The bar’s height is proportional to the number of Twitter accounts belonging to this community. These are computed every Monday.

Between each period, for each community, we computed the number of community-changing accounts, and generated the global structure synthesizing the development of the political landscape. This structure can be visualized with an alluvial diagram (cf. Fig 6), a visualization that summarizes the reconfigurations of the political space over a period of 11 months, with sufficient resolution to provide an accurate narrative of this presidential election (Fig 7). It can be seen that whereas most of the major political events can be interpreted on this view, it also shows more subtle relationships between communities that are not the subject of official declarations as is the case for rallies or victories in primaries. For example (see analysis below), we can observe the distancing of the Sarkozy community from that of Fillon, shortly before the Penelopegate affair, followed by the rallying of a considerable number of Sarkozy activists to Dupont-Aignan. This last rally was relatively ephemeral, since most of these accounts ceased to follow Dupont-Aignan when he himself rallied to Le Pen between the two rounds of elections.

**Fig 8:**
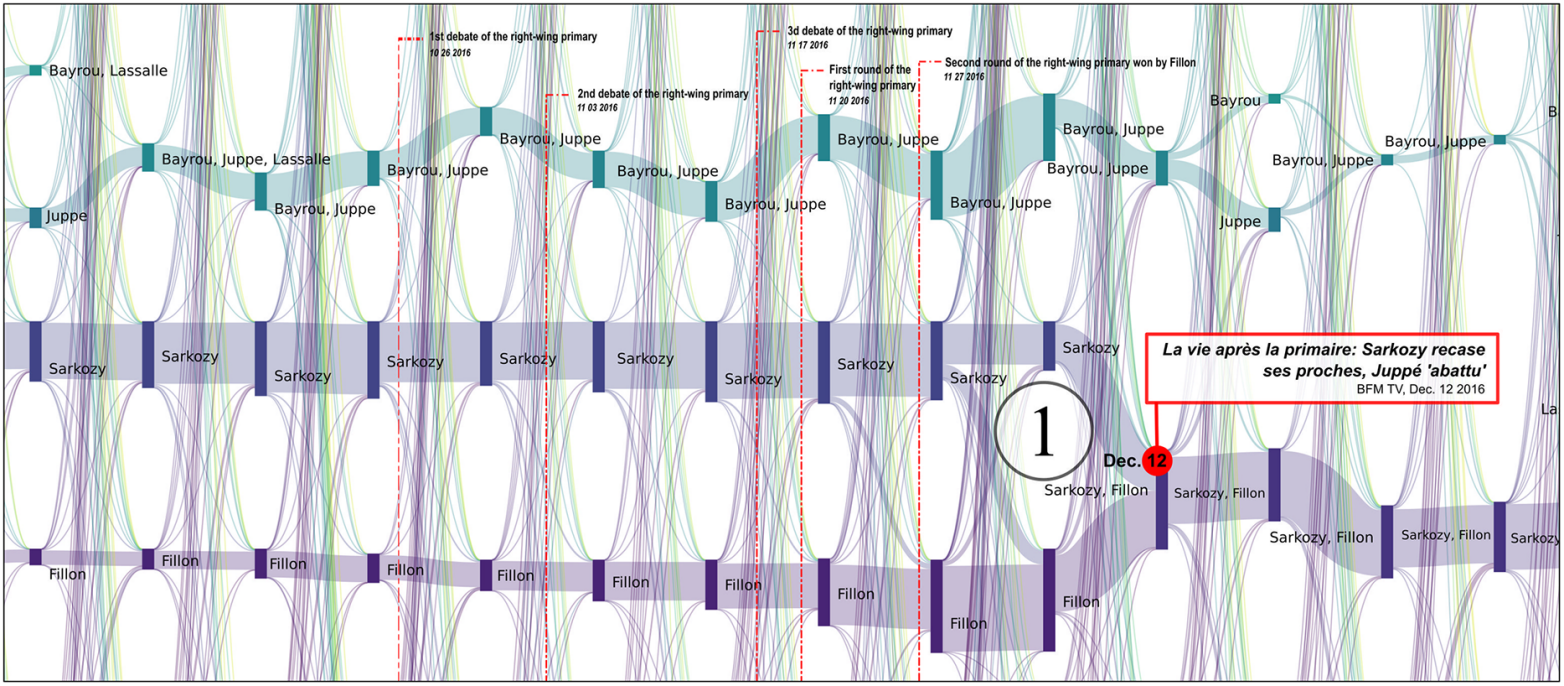
Reconfiguration of political communities at the time of the primaries held by the right-wing party. These primaries were marked by three debates and two votes. One can observe the progressive and regular reinforcement of the Fillon community, week after week, although no-one was predicting his victory in the second round, since Juppé was tipped by the polls to win. As a result of the first round, following the surprise victory of Fillon and the equally surprising elimination of Sarkozy (president of the *Les Républicains* party and ex-President of France), only a minute portion of the Sarkozy community moved to that of Fillon. The Sarkozy community was reluctant to join the Fillon community, and Sarkozy spent several days negotiating positions for his members within the Fillon structure. The Juppé community, however, immediately distanced itself from the new political orientations adopted by the party. This right-wing split was apparent in the pre-electoral environment (see Fig 1). Following the failed return of Juppé in March 2017 (see Fig 11), part of his community moved to join Macron. The bifurcations observed here are clearly summarized by an article published by BFMTV on 12 December 2016: *“Life after the primary: Sarkozy relocates his close allies, Juppé is ‘down’”*. Each vertical bar corresponds to a political community on Twitter, labelled with the accounts of candidates who were active in the community. The height of the bar is proportional to the number of Twitter accounts belonging to this community. These are computed every Monday.

**Fig 9:**
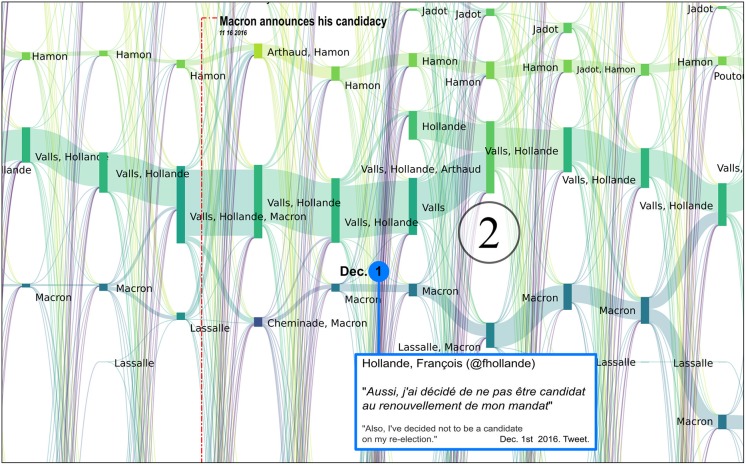
Reconfiguration of political parties when the incumbent president François Hollande abandons the race for reelection. This declaration was made shortly after Macron had announced his own candidacy. The alluvial graph clearly shows that Hollande’s announcement revitalized the Macron community, at the expense of Valls. Whereas the size of the Macron community had remained stable at the time when he announced his candidacy (427 accounts the previous week, 561 the week of the announcement, 472 the following week), it jumped to 749 accounts during the week when Hollande renounced standing for a second term, and then to 1479 accounts the following week. From that time onwards, this community continued to grow regularly until the elections. This change coincides with a slowdown in the Valls-Hollande community, the right-wing section of the *Parti Socialiste* that was ideologically closest to Macron. However, the Hamon community, which is further to the left, remained relatively stable during this period. Each vertical bar corresponds to a political community on Twitter, labeled with the accounts of candidates who were active in the community. The height of the bar is proportional to the number of Twitter accounts belonging to this community. These are computed every Monday.

**Fig 10:**
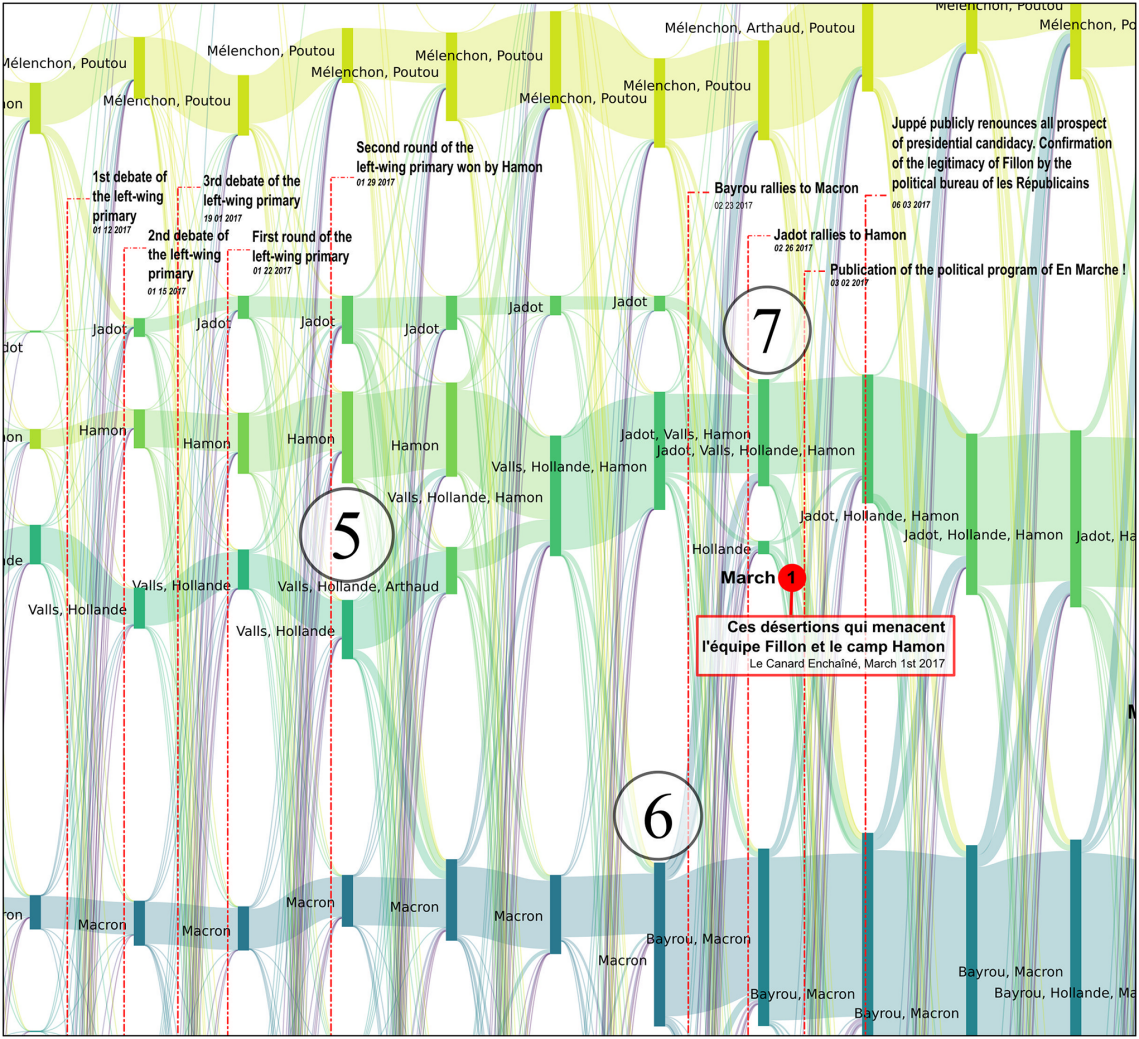
Reconfiguration of political parties at the time of the primaries of the left-wing party. Benoît Hamon (36.03% in the first round and 58.69% in the second round) and Manuel Valls (31.48% in the first round and 41.31% in the second round) were the two favorites of the primaries and represented two different currents in the Parti Socialiste, referred to by Valls as the “irreconcilable lefts” during his campaign. Although François Hollande did not officially state his preference for one particular candidate, Valls declared that he had his support (see for example in *Le Monde* on 4 January, 2017: “*According to Valls, he has Hollande’s support*”). Indeed, in our reconstruction, during the full period of observation, the Hamon community remains quite separate from the Valls community. For most of the time, the latter community is also the Hollande community, in particular at the time of the primaries, thus revealing the ideological proximity between these two political figures. Following Hamon’s victory, a controversy broke out concerning the commitment made by the primary candidates to align themselves with the winner, whereas some were already clearly indicating their preference for Macron. This indecision is clearly visible on the alluvial graph, with significant movements of activists from the Valls-Hollande community towards the Macron community immediately following the second round of the primary. From the 2,075 accounts making up the Valls-Hollande community shortly before its fusion with the Hamon community on February 13 (Valls also joined the Hamon community), only 959 accounts remained. Significant movements of activists continued to take place between these two communities, until the time of the first round of the presidential election. Although Valls finally gave his official support to Macron on March 29, he was no longer present in the Hamon community after March 13. He started appearing in the Macron community on April 17, and remained until the second round, thus indicating his active support. One can also note in this zoom the rallying of the ecologist Jadot to Hamon on February 23, 2017, which is immediately revealed by the fusion of his community with that of Hamon. Each vertical bar corresponds to a political community on Twitter, labeled with the accounts of candidates who were active in the community. The height of the bar is proportional to the number of Twitter accounts belonging to this community. These are computed every Monday.

**Fig 11:**
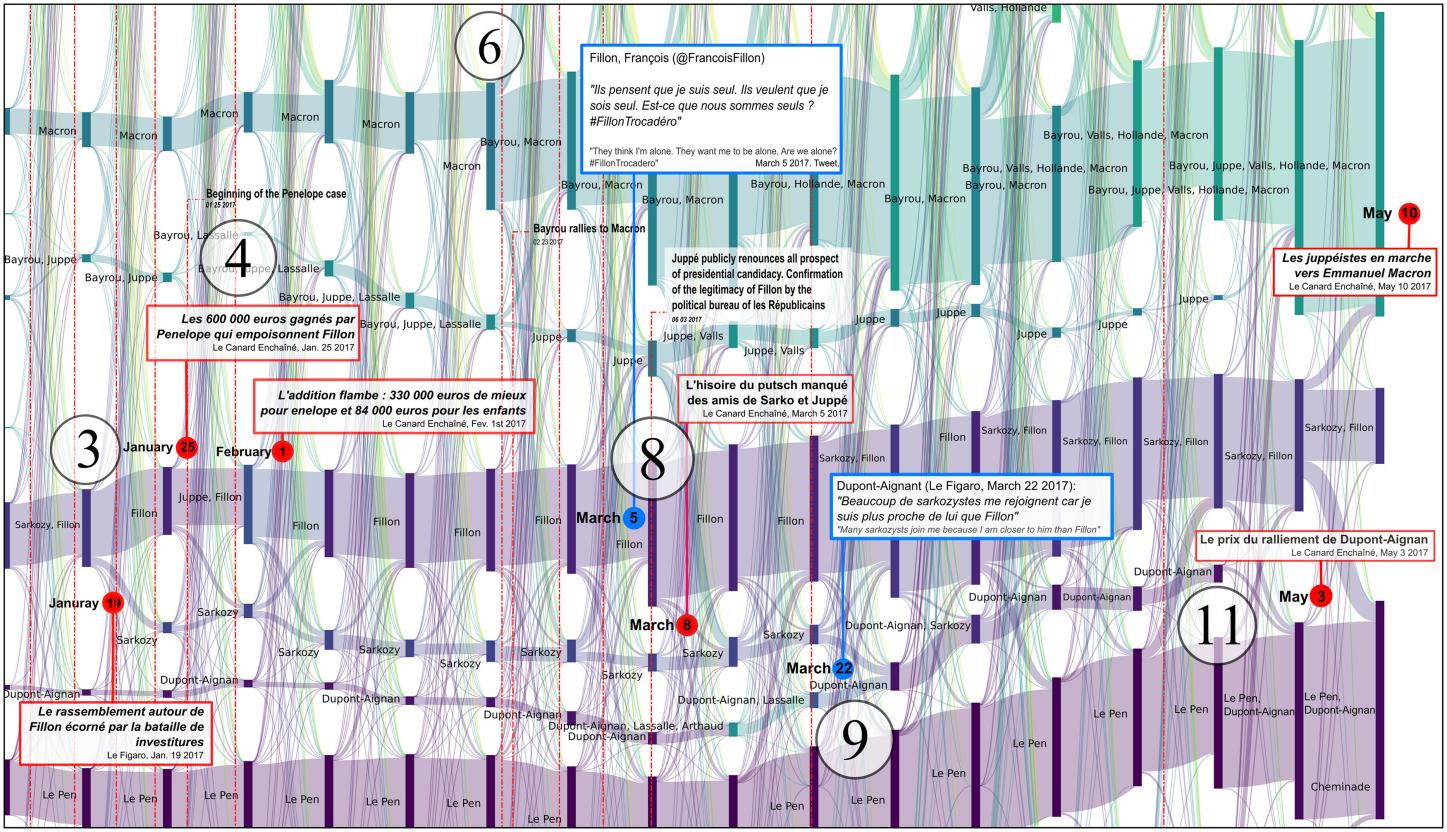
Reconfiguration of political communities at the time of Penelopegate. Penelopegate arrived at a time when Fillon’s following was weakening as a consequence of a controversy surrounding the investiture for parliamentary elections, which was badly received by Sarkozy supporters. Some of these individuals had already started to move away from the *Les Républicains* community, as early as January 23, 2017, and had formed a separate community throughout the full duration of this controversy. Various revelations made by the *Canard Enchaîné* led to major movements of activists between different communities, with the communities supporting Juppé (a potential alternative, i.e. a “plan B”, for Fillon’s candidacy) and Sarkozy being progressively reinforced. On March 1st, the day when Fillon was summoned in the context of his investigation, a large proportion of Sarkozy supporters were in support of Juppé’s return, in a final attempt to push Fillon to resign, which was revealed by an unusual flow of accounts from the Sarkozy community towards that of Juppé. The public gathering that took place on March 5th at the Trocadéro Square (Paris) brought an end to the perspectives of Plan B. Although the Fillon community gained a large number of new activists following this takeover, some of the Sarkozy and Juppé followers kept their distance, and finally joined, respectively, Dupont-Aignan and Macron. Sarkozy himself withdrew for several weeks, which led to his absence from most of the Fillon communities between January 16th and April 10th. It was only from April 7th, that he actively gave his renewed support to Fillon’s candidacy, as was for example mentioned in the *Parisien* headline on that day *“Presidential elections: Sarkozy (finally) supports Fillon”*, and could be seen by his constant presence within the Fillon community starting on April 10th 2017. As can be seen, the bifurcations observed in the *Politoscope* take place at the same time, or in some cases even anticipate comments in the press concerning the activity of political communities. Each vertical bar corresponds to a political community on Twitter, labelled with the accounts of candidates who were active in the community. The bar’s height is proportional to the number of Twitter accounts belonging to this community. These are computed every Monday.

**Fig 12:**
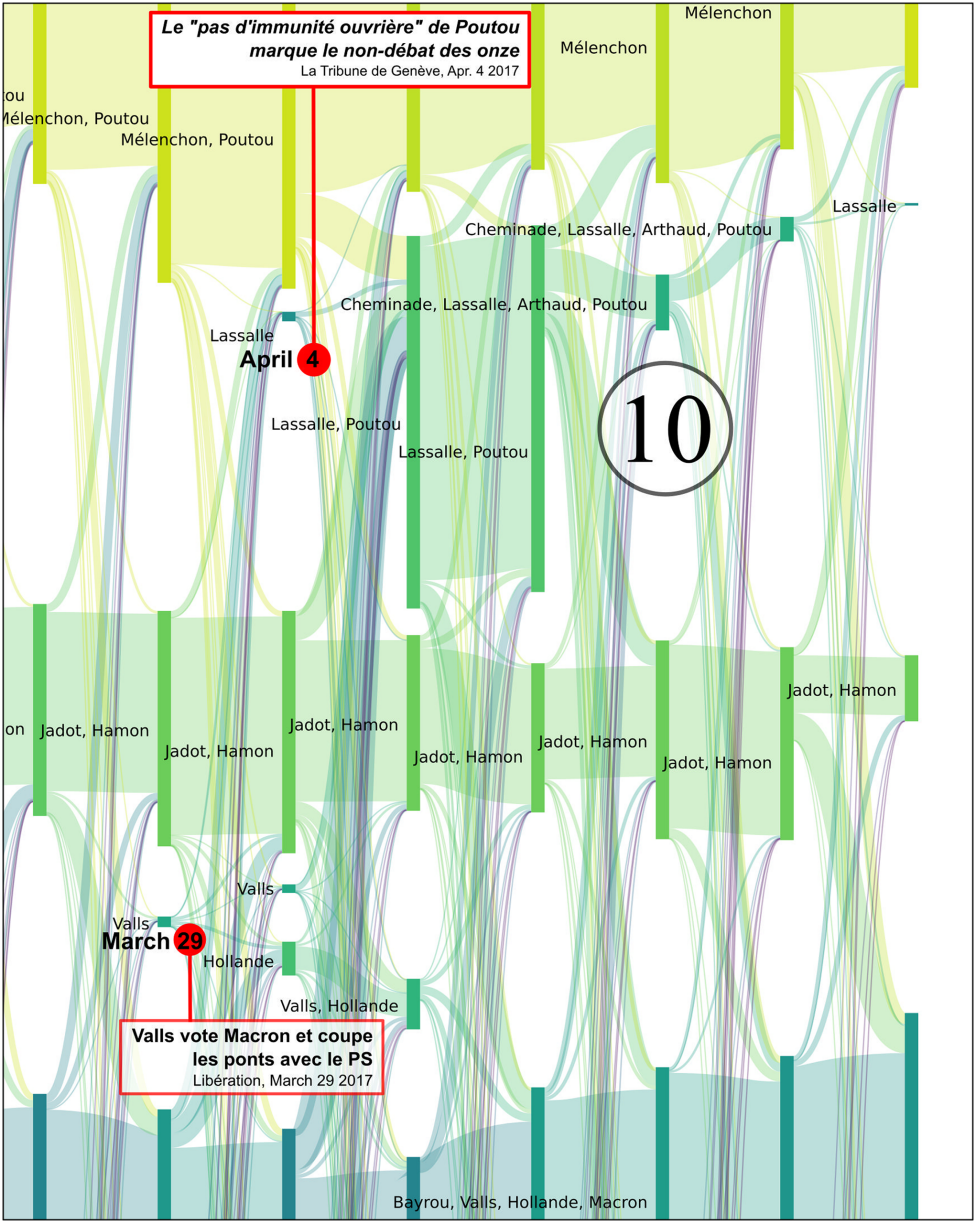
Reconfiguration of political communities at the time of the televised “*Le Grand Débat*” on April 4th, 2017. Philippe Poutou created a buzz during this confrontation when he openly condemned the trickery and corruption of candidates Fillon and Le Pen, by criticizing their anti-system attitudes: *“When we are summoned by the police, we have no working-class immunity, we go to the police station.”* His remarks were relayed by most of the media, and received more than 1 million online consultations on YouTube. Following this intervention his Twitter community, which until that time had been almost inexistent, and often overlapped with that of Mélenchon, developed considerably (to more than 13,000 accounts) over a short period of time.

The many episodes that can be described by this alluvial visualization are all evidences that an analysis of Twitter political tweets can successfully reconstruct the political landscape dynamics: key events of the presidential campaign correspond to key elements in the reconstruction, and thus in the visualization (like merge or split of communities, abrupt change in community size, etc.). Among others, the following events can easily be identified as merge *[M]*, split *[S]*, emergence *[E]*, fade-out *[F]* or abrupt size change *[C]* of some of the communities and associated to press coverages (cf. Fig 7 where each of the following event has been identified):

1. **2016/11/27-2016/12/01** [53]: *[M + F]* the reconfiguration of the right after the primaries with the alliance after negotiations between Fillon and Sarkozy and the fade-out of Juppé’s community,
2. **2016/12/01** [54]: *[E]* the leverage effect of Holland’s renunciation on the Macron’s campaign,
3. **2017/01/17** [55]: *[S]* Frictions among *les Républicains* after disputed legislative investitures, including that of Nathalie Kosciusko-Morizet (NKM), to whom Fillon offered an easy-to-win district,
4. **2017/01/25** [56]: *[M + S + E]* Start of the Penelope Gate, some fillionnists joint the Sarkozy community, the Alain Juppé community reappears,
5. **2017/01/29** [57]: *[C + M + S]* Second round of the left-wing primary won by Hamon,
6. **2017/02/23** [58]: *[M]* Bayrou rallies to Macron,
7. **2017/02/26** [59]: *[M]* Jadot rallies to Hamon,
8. **2017/03/05-06** [60]: *[S + M + C]* François Fillon organizes a rally in Trocadéro (Paris). He announces 200 000 attendees (including leading supports of Sarkozy) and presented this as a support to the upholding of his candidacy, sarkozists support Juppé come back but this latter finally publicly renounces all prospect of presidential candidacy. Confirmation of the legitimacy of Fillon by the political bureau of *les Républicains*, which was originally convened to ask that he be dismissed [61],
9. **2017/03/23** [62]: *[M]* Dupont Aignan claims that “many sarkozists are joining my movement”,
10. **2017/04/23** [63]: First round of the presidential election lead by Macron and Le Pen. Mélenchon refuses to give voting instruction to his supports, in contradiction to what he did in 2002 (he called to vote against the *Front national*),
11. **2017/04/28** [64]: *[M]* Dupont-Aignan rallies to Le Pen,

We analyze these key events in more detail in Figs 8, 9, 10, 11 and 12.

The reconstruction method we propose reveals the deep reconfigurations of a political landscape during a presidential campaign in multi-polar political environment like the French one. By describing the behavior of a set of activists, corresponding to a wide spectrum of political affiliations, it is positioned at an intermediate level between political leaders and their electoral base.

It provides access to a level of observation that is rarely accessible in the framework of sociological studies or in political sciences. In total, between Aug 1st 2016 and May 8 2017, nearly 190,000 individual accounts have been associated to a political community, with most communities involving several tens of thousands of activists (see Table C in S1 File). These communities are made up from a large number of opinion leaders and political outlets. From these results, we can safely assume that the political tribulations of sub-populations within this set of individuals are indicators, and in some instance the precursors, of political change and reconfiguration.

In the following sections, we propose insights and open questions about what can be learned from such reconstructions beside the accurate and synthetic narrative of a political campaign. We explore two distinct feature of communities: their social structure and their semantic profiles.

### Characterization of communities structure and political engagement

#### Stability and composition of the communities

Political communities evolve by gaining or losing members who were not previously included among politicized accounts on Twitter, or by recruiting new members from other communities. Our reconstruction of political landscape describes the co-evolution between the structure of a community and its members’ opinions. Communities govern the circulation of information, and the members of a given community are exposed to specific content. However, this exposure can contribute to changes in their opinion and eventually cause them to switch to another community, thus modifying the overall structure of the communities.

An important challenge in understanding the evolution of a political landscape is to understand what are the structural particularities of each community and how they might impact their evolution.

We analyzed the stability and composition of the communities of the 6 candidates who used Twitter most extensively in their campaign. A given candidate *c* may appear in communities at different periods of time over a set of periods T. Since these 6 candidates have always had distinct communities, for sake of clarity, we will name “the community of *c*” the set of all communities *c* has belonged to over the set of time periods T (noted $C_{c}^{T}$).

In addition, in order to ensure that the indicators calculated on the communities are comparable, we calculated them over the set T of periods covering February 1st to April 22th 2017. February 1st is the day all the names of the candidates running for the presidency have been confirmed. April 22 2017 is the eve of the first round.

The analysis of the cumulative distribution of time spent in a given community (see Text E in S1 File) shows that for most communities (except Asselineau who was a “small” candidate with 0.92% of votes), about 15% of accounts were present for just 1 day, and more than 10% of accounts were involved for more than 40 days.

There is thus a general political community structure on Twitter, comprising a core of activists who remain for several weeks within the community, a considerably more volatile peripheral membership, and an intermediate set of accounts covering a broad spectrum of participation.

This however loosely characterizes the structure of a political community and the political engagement of its members. To refine the analysis of the communities, we propose several indicators that contribute to characterize quantitatively the types and degree of political engagement of their members.

#### Indicators of political engagement

Let $C_{c}^{T}$ be the community of a candidate *c* over the set of periods T. We can compute the number of tweets categorized in this community and the number of accounts having been involved at least one day in that community.

The *days contribution* of every account *a* to every community $C_{c}^{T}$ is the number of days *a* has been involved in $C_{c}^{T}$ over the set of periods T. Averaged over a community, we get the average number of days accounts contributing to $C_{c}^{T}$ have been involved in $C_{c}^{T}$ (designed by < *days* >). The cumulative distribution of days spent within a community is given by Fig F in S1 File.

Overall during T, within the Twitter communities of the 6 leaders that used the most intensively Twitter for their campaign (resp. all the Twitter communities), there were 18, 018, 417 tweets emitted (resp. 19, 483, 583) by 92, 762 distinct accounts (resp. 105, 418) with an average number of tweets per account of 184.82 (resp. 194.24).

Let the ***commitment*** of an account *a* toward a candidate *c* be the percentage of *a*’s community tweets that belong to $C_{c}^{T}$: $co\left(a,c,T\right)=\frac{N_{tw}^{T}\left(a,c\right)}{N_{tw}^{T}\left(a\right)}$, where $N_{tw}^{T}\left(a,c\right)$ is the number of tweets issued by *a* within $C_{c}^{T}$ and $N_{tw}^{T}\left(a\right)$ is the total number of tweets issued by *a* within any community during the same period. This index indicates the extent to which *a* contributes to the circulation of ideas in the community of *c* when *a* is involved in interactions with political groups. The *average commitment* of a community $C_{c}^{T}$ is then defined as the average of *commitments* of accounts participating to $C_{c}^{T}$: $<co\left(C_{c}^{T}\right)>=\frac{\Sigma_{a\in C_{c}^{T}}co\left(a,c,T\right)}{\left|\{a\in C_{c}^{T}\}\right|}$.

Several other indicators can be computed at the account level. Since one account can be involved in several political communities over a set of periods (that could reflect the fact that the owner of the account is undecided), we generalized these indicators at the community level by weighting them by the *commitments* of the accounts (see Tables 6 and 7):

- the ***activity*** of an account *a* over T is the number of political tweets emitted for that set of periods (*i.e*. number of tweets captured by the *Politoscope*). The *activity* of a community $C_{c}^{T}$ is then defined as the average of weighted *activity* of accounts participating to $C_{c}^{T}$.
- the ***politicization*** of an account *a* over a set of periods T is the percentage of political tweets emitted by an account (*i.e*. number of tweets captured by the *Politoscope* during T over the total number of estimated tweets during the same period). Since we don’t follow most of the accounts that interact with the *Politoscope*, we estimate for every account the total number of tweets emitted during T as the difference between the total number of tweets for that account mentioned in the meta-data of the last observed tweet and the same quantity mentioned in first observed. This estimate can be biased in two ways. First we might not capture all political tweets. Second, users can delete tweets. This decreases the number of tweets mentioned in the meta-data but not the number of tweets seen in the *Politoscope*, leading *politicization* to be more than 1 for some users. Consequently, we set the maximum of *politicization* of an account to 1. As these two biases should apply equally to all communities, we consider this estimate as a good indicator for comparing the degree of politicization of communities. The *politicization* of $C_{c}^{T}$ is then defined as the weighted average of *politicization* of accounts participating to $C_{c}^{T}$.
- the ***political integration*** of an account *a* over T is the percentage of tweets emitted within any community among the total number of political tweets captured with *a* as author. An account that tweets mostly in interaction with a community will have a high *political integration* index. On the contrary, an account that mostly tweets political content not in relation with any structured group on Twitter (*i.e*. this account if often categorized as belonging to the “sea”) will have a low *political integration* index. The *average integration* of $C_{c}^{T}$ is then defined as the weighted average of *political integration* of accounts participating to $C_{c}^{T}$.

**Table 6:** Detailed statistics for communities of the 6 candidates with the most intensive use of Twitter in their campaign (*T*_*k*_ ∈ [Feb. 1st 2017–April 22 2017]): Average time spent per account inside the community (< *days* >), total number of tweets from that community (|*Tweets*|), number of accounts present at least once in the community (|*accounts*|) and number of accounts present at least once in the community weighted by the commitment (|*weighted accounts*|). Outliers are bolded for greater readability.

Name	< *days* >	|*Tweets*|	|*accounts*|	|*weighted accounts*|
Jean-Luc Mélenchon	12.11	3,110,517	22,118	17,375.96
Benoit Hamon	11.69	1,828,418	18,950	14,340.52
Emmanuel Macron	13.92	3,643,822	**24,803**	**18,717.05**
François Fillon	**18.49**	**5,032,109**	18,264	14,428.71
Marine Le Pen	**17.18**	3,693,650	18,438	14,622.39
François Asslineau	8.95	709,901	6,597	3,713.01

**Table 7:** Detailed statistics for communities of the 6 candidates with the most intensive use of Twitter in their campaign (*T*_*k*_ ∈ [Feb. 1st 2017–April 22 2017]): Average commitment of accounts in the community (< *commitment* >); quantities weighted by the commitment: Average number of tweets per account (< *activity* >), political integration (< *integration* >) and politicization (< *politicization* >). Outliers are bolded for greater readability.

Name	< *commitment* >	< *activity* >	< *integration* >	< *politicization* >
Jean-Luc Mélenchon	93.88%	140.63	54.19%	2.62%
Benoit Hamon	91.96%	96.49	57.16%	**6.16**%
Emmanuel Macron	91.93%	146.91	54.51%	2.91%
François Fillon	93.24%	**275.52**	**63.40**%	5.42%
Marine Le Pen	**95.74**%	200.33	60.72%	3.63%
François Asslineau	85.43%	107.61	56.05%	2.80%

These indicators makes it possible to characterize different types of political engagement that could be specific to some communities. In turn, the identification of some particularities at the level of the communities could point toward specificities in the mode of recruitment of new members.

The Asselineau community is clearly an outlier in this set of political groups with by far the smallest community size, lowest commitment level and a politicization and an integration among the lowest.

Excluding this atypical community and ranking the other five along a left-right axis, we observe a U shape for the commitment which indicates that people with extreme political views tend to be the most committed to their ideology. This is to be compared with the observation of Preotçiuc-Pietro *et al*. [65] in the US that the people whose political ideas are most extreme have the highest level of political engagement.

Le Pen and Fillon community members are most active on Twitter with an activity of 200.33 (resp. 275.52). They are also the most long-lasting ones inside communities (resp. 17.18 and 18.49 days) and the ones with the highest integration (resp. 63.40 and 60.72).

While the average number of days spent within a community is directly linked to the activity level, high integration means that more than for other communities, when members of Fillon and Le Pen communities express their political views on Twitter, it is to rally some particular political group. These two communities are however distinct with respect to the commitment of their members, Le Pen community members having the highest commitment score of all communities (95, 74%). Combined together, high commitment and high integration of Le Pen community members means that they are the most inflexible of all political activists in their opinions and the most committed to a leader. Given the fact that Le Pen community is also one of the most active on Twitter, it is the one with the most stable community core. This probably explains why right-wing leaders are unable to attract far-right voters despite their stated will to do so; and why, as reported in [42], instead of fighting Le Pen who was in good position to pass the first round of the election, most leaders preferred to fight among themselves to win the opportunity to compete against her in the second round of the election.

The Macron and Melenchon communities are by far the ones with the largest numbers of accounts (24*k* and 22.1*k* respectively) but also the ones with the smallest integration and politicization and quite low averages of days spent inside the community. This reflects the highly dynamics character of these both candidacies which final scores were unanticipated at the beginning of the campaign and that got momentum when getting closer to the end of the campaign. This also suggests that they were successful in recruiting a low politicization audience.

Hamon community is the one with the highest politicization (6.16%) with moderate levels of commitment and integration which tends to indicate that it was a community with a strong political background that was more focused on ideas than on the support to a particular leader. This is in line with the analysis of [42] that demonstrated from the analysis of the leaders mentions in the *Politoscope* data that the Hamon community was the one that expressed its political ideas in the most detached way from any reference to a candidate or a party. The low average number of days spent in the community reflects the useful voting phenomenon (*vote utile*): at some point (around March), many people from the Hamon community joined either the Melenchon or the Macron communities when the collective belief ended on the idea that Hamon would not qualify for the second round. And indeed, he ended up far behind all the other candidates.

The decomposition of the online engagement of political communities members into *activity*, *politicization*, *political integration* and *commitment* allows to discriminate different types of political groups. As a perspective, we conjecture that the analysis of their evolution through time would be a good predictor of the destinies of communities.

### Propagation of political information

Social systems are among the most sophisticated complex systems in terms of entanglement of levels and multi-scale dynamics. We should consequently expect their multi-level structure to be a key factor in the diffusion phenomena, social networks being a privileged place to observe their role.

In this section, we look at how communities act on the dissemination and circulation of political information, defending certain themes or highlighting certain topics with a specific framing. Particular attention is paid to the phenomenon of fake news which can be studied here in all its community dimension.

#### Semantic profiles and the echo chamber effect

Given our reconstruction method, a first intuitive results is that political communities generate strong *echo chamber effects* [66]: the analysis of retweet propagation (see Text F in S1 File) shows that the vast majority of tweets are retweeted within their original community.

To go beyond the *echo chambers* effect observation, we have selected 11 of the main topics of discussion for this election (see Text G in S1 File) and analyzed the evolution of the semantic profiles of the tweets circulating within each community with respect to these topics. This reveals a high heterogeneity among communities both in terms of topics, framing and agenda.

More precisely, we analyzed the specificity of the political discourse, as a function of theme, at several levels:

- Diversity of the political parties dealing with each theme,
- Diversity of the themes discussed by a given political party,
- Diversity of the expressions used by each political community.

For each theme, the computed value of entropy, based on the number of communities addressing this theme, shows that all of the latter are covered by an average of approximately six communities, with a lesser degree of diversity for the themes of *homeland security* and *immigration and multiculturalism*, which are strongly promoted by the extreme right (see Table D in S1 File).

For each political community, the computation of the diversity of the addressed themes clearly reveals communities heterogeneity (see Table 8) and a differentiated positioning of themes on the political agenda, at different times during the campaign (see the analysis of the dynamics of themes by community in Text G.2 in S1 File). This heterogeneity can also be found at the level of the vocabulary used by the members of each political community (see Table 9). However, as this analysis does not search for all possible themes, the present study cannot be used to qualify the candidates with respect to the diversity of the themes they address.

**Table 8:** Entropy of the themes addressed by each candidate, and its 3–community. The two dominant themes as well as their associated percentages are also shown. The maximum entropy is 3.46, if the 11 themes are discussed in a uniform manner. With respect to the selected themes, Benoît Hamon is the candidate who uniformly addressed the greatest number of themes, whereas François Asselineau is the candidate who was the most focused on a subset of themes (including foreign policy and democracy). The variations in attention paid by the candidates to these themes are plotted in Figs I to N in S1 File.

Name	Entropy	# themes	Dominant themes	%
François Asselineau	2.63	6.2	foreign policy, democracy	57.7
François Fillon	3.06	8.3	immigration and multiculturalism, democracy	33.5
Benoît Hamon	3.19	9.1	employment, foreign policy	39.3
Marine Le Pen	2.78	6.9	immigration and multiculturalism, foreign policy	49.0
Emmanuel Macron	3.03	8.2	foreign policy, democracy	44.2
Jean-Luc Mélenchon	3.10	8.6	democracy, foreign policy	37.3
All candidates	3.08	8.4	foreign policy, democracy	36.9

**Table 9:** Entropy of the keywords used by each candidate and its 3-community. The 10 dominant keywords as well as their percentage are also shown. The maximum entropy is 10.64, when the 1596 keywords are discussed uniformly.

Name	Entropy	*N*^0^ keywords	Dominant keyword	%
François Asselineau	5.98	63.2	Frexit, Europe, poll, media, Brexit, nation, elected officials, democracy, treaties, the Euro	48.7
Benoît Hamon	7.77	218.6	republic, youth employment, universal income, democracy, Europe, elected officials, ecology, nation, children	21.1
François Fillon	7.75	215.8	media, poll, republic, upturn, elected officials, unemployed, police, nation, work, security	19.6
Emmanuel Macron	7.71	209.1	Penelope Fillon, republic, elected officials, work, poll, justice, unemployed, Europe, police, indictment	19.8
Marine Le Pen	7.82	226.2	nation, islamists, patriotism, police, media, poll, unemployed, migrants, elected officials, terrorism	20.7
Jean-Luc Mélenchon	7.53	184.8	French labor law, nation, work, republic, nuitdebout, poll, elected officials, media, Penelope Fillon, unemployed	24.3
All	8.23	301.23	nation, republic, poll, elected officials, media, unemployed, work, Frexit, police, Europe	17.5

The *Politoscope*, the companion platform of this paper (https://presidentielle2017.politoscope.org/dashboard), makes it possible to explore, for each of the main candidates and each of the eleven themes, the changes in the 15 most significant keywords for each of the respective candidates. One can also observe, in addition to the semantic specificity of the communities with respect to these themes, a variation as a function of time for the different candidates, with the usage of certain keywords appearing, strengthening or disappearing over time (see Text G.3 in S1 File for details of the method and examples).

#### Fake news circulation within the communities

Fake news is an ill-defined problem as it seems to cover propaganda, disinformation and false allegation in the media. Nevertheless studying the effectiveness of these phenomena is of paramount importance. Fake news are currently discussed in the United State of America, particularly in the context of the 2016 presidential election [67, 68] and is now the subject of an adaptation of legislation in France since the attempts to manipulate public opinion during the French 2017 presidential elections [69].

The question of how comparatively true and false information spreads on a social networking site like Twitter and which contexts influence this spread is an important issue for the “science of fake news” that should help to solve the post-true crisis [70, 71]. To assess the importance of the reconstruction of political communities in the understanding of this phenomenon, we used the *Decodex* database, an initiative from *Le Monde*, one of the major French newspaper (center-left oriented), that has collected, throughout the year 2017, URLs of web pages and Facebook posts that include fake news [72]. URLs referring to the same fake news (*e.g*. “*Jean-Luc Mélenchon has a Rolex*” or “*More than 30% of Emmanuel Macron’s campaign was financed by Saudi Arabia*”) have been grouped together in *stories* and associated to a *debunk*, a blog post in which *Le Monde* journalists unveil and explain why this news is a fake. There are 179 stories and 179 debunks in total (see Files A6 and A7 at DOI: 10.7910/DVN/AOGUIA for a json version).

The *Decodex* database is a good entry point to identify the stakes of the fake news phenomenon through the lens of our political landscape reconstruction.

#### Differential echo chamber effet for fake news

After cleaning and normalizing the *Decodex* URLs; and having double checked that the tweets mentioning a link to a fake news or a debunk have really for purpose to broadcast them (see Text H in S1 File), we have studied their diffusion. We observed only 4 888 shares of fake news links inside our dataset of 60 million tweets (0.081%). This indicates that either fake news are not heavily shared in France by people interested in politics on Twitter or that the *Decodex* failed to identify a significant proportion of fake news references on Twitter. Likewise, the debunks were shared only 1 275 times.

There is an average number of 27.3 sharing per fake news story whereas the average number of share for debunks is 7.12. Consequently, for this fake news/debunks dataset, fake news stories were shared almost four times more than debunk news.

This difference may be structural or situational in nature. It could also be due to the fact that we might miss a significant proportion of debunks for these set of stories published by newspapers other than *Le Monde*. It is nevertheless comparable with the results of Vosoughi *et al*. 2018 [73] on US political fake news on Twitter: “falsehood diffuse significantly farther, faster, deeper, and more broadly than the truth in all categories of information, and the effects [is] more pronounced for false political news.”

Still, our reconstruction of political communities invite us to reconsider Vosoughi *et al*. results given of the new perspectives if offers on fake news diffusion. Computing the entropies of the fake news and debunks diffusions at the community level, we found that the phenomena is reversed (see Table 10): on average, fake news spread in much less communities (3.78) than debunks (7.67), that in turn themselves spread in much less communities than the average of all political tweets (13.45).

**Table 10:** Entropy and average number of communities [in brackets] reached on average by fake news, debunks and tweets with or without URL mention over the period. We also present the result when considering all communities as a single one except for the 6 main political online communities identified during the French presidential election. Tweets without communities (43.44%) were considered as a single community for the processing of entropy. For comparison with the debunks and fakes news dataset, identified by the URLs they spread, we also provide the global statistics for the subset of tweets that mention URLs.

entropy [# communities]	All communities	6 majors communities
Fake news retweet (n = 4,888)	1.92 [3.78]	1.53 [2.88]
Debunks retweet (n = 1,275)	2.94 [7.67]	2.23 [4.69]
All retweets with url (n = 7,318,878)	3.75 [13.45]	2.28 [4.85]
All retweets (n = 43,101,891)	3.72 [13.17]	2.54 [5.82]

Fake news might reach on average more people than other political news but they spread more locally. This *immergence effect* of the community structure on micro-dynamics, that entails a reversal of perspective during the transition from the micro-level (fake news reach more accounts than other news) to the macro-level (fake news reach less communities than other news) is very important when addressing this issue from a societal perspective.

#### On Twitter, fakes news are primarily from some political communities, not from the sea

The number of shares is not the only relevant variable. The analysis of fake news and debunks circulating inside the five major political communities (Emmanuel Macron, Marine Le Pen, François Fillon, Jean-Luc Mélenchon and Benoît Hamon) reveals some clear patterns in terms of localization and temporal distribution.

18.9% (922) of the shares of fake news correspond to tweets with no community affiliation, *i.e*. from what we have called “the sea” (we remind that communities are processed each day and the community of a tweet is the community attributed to the account issuing the tweet at the time of the tweet issuance). Since globally 43.44% (25 695 888) of tweets have no community, this means that the accounts that are the least politicized or not involved into some group of activists share on average much less fake news than the most politically integrated accounts.

There is also a significant heterogeneity in the practice of fake news sharing within communities. The communities of Fillon (right-wing) and Le Pen (extreme-right wing) are the most active with respectively 50.75% and 22.21% of the total number of fake links shares (89.9% of fake news shared within communities); while the community of Hamon is the least active with only 0.43% of the total shares (cf. Table 11).

**Table 11:** Number of users, number of tweets mentioning a fake news link and the proportion of early spreaders for the tweets containing fake news links. Early spreaders are users that have been among the first 1/30 percentile of users to share a fake news.

Candidates	#Users	#Tweets	% early spreaders
François Fillon	1236	2481	0.06
Benoît Hamon	19	21	0
Marine Le Pen	784	1086	0.05
Emmanuel Macron	61	65	0
Jean-Luc Mélenchon	11	143	0.03
Other communities	123	170	0.03
Sea	614	922	0.03

The temporal dynamics is also noteworthy. Insides stories, we ranked tweets by their date of issuance with a time resolution of one second. Removing the stories that have been shared less than 30 times to mitigate small size effects, we computed the percentage of *early spreaders* per communities defined as the users that rank in the 1/30 percentile (cf. Table 11). The Fillon community is the one with the highest percentage of early spreaders (6% of accounts from that community are early spreaders), before the Le Pen community (5%); while none of the Hamon and Macron communities accounts are among early spreaders.

To summarized, based on the *Decodex* fake news list, the Fillon community is both the one that was the quickest to spread fake news and the major source of fake news sharing.

We must not make too hasty conclusions about the origins of these fakes news. Several hypothesis are compatible with these preliminary results. Within communities, the status of the accounts spreading fake news are of utmost sociological importance. It could be the work of leaders, second fiddle, activists, bots or accounts that try to influence a particular community through astroturfing (these accounts could even be held by foreign persons). The estimation of this social status is out of the scope of the present paper but these accounts have sufficiently close interactions with political communities to be associated with them through our community detection method.

The analysis of the tweets containing debunk links offer a radically different pattern (cf. Table 12). While 38.11% of debunks circulated within the sea, the most active community is the one of Macron with 39.18% of the total of the debunks alone. Moreover, 12% of the users spreading debunks inside the Macron community are among the first 1/10 percentile of user sharing a debunk when they are sorted by the time of the tweet (early spreaders). The Mélenchon community is also very reactive with 14% of debunks early spreaders even though they are much less active.

**Table 12:** Number of users, number of tweets and the proportion of early spreaders for the tweets containing debunk links. Early spreaders are users that have been among the first 1/10 percentile of users to share a debunk.

Candidates	#Users	#Tweets	% early spreaders
François Fillon	15	15	0.07
Benoît Hamon	47	69	0.11
Marine Le Pen	7	8	0
Emmanuel Macron	361	499	0.12
Jean-Luc Mélenchon	83	98	0.14
Other communities	82	100	0.12
Sea	345	486	0.05

**Fig 13:**
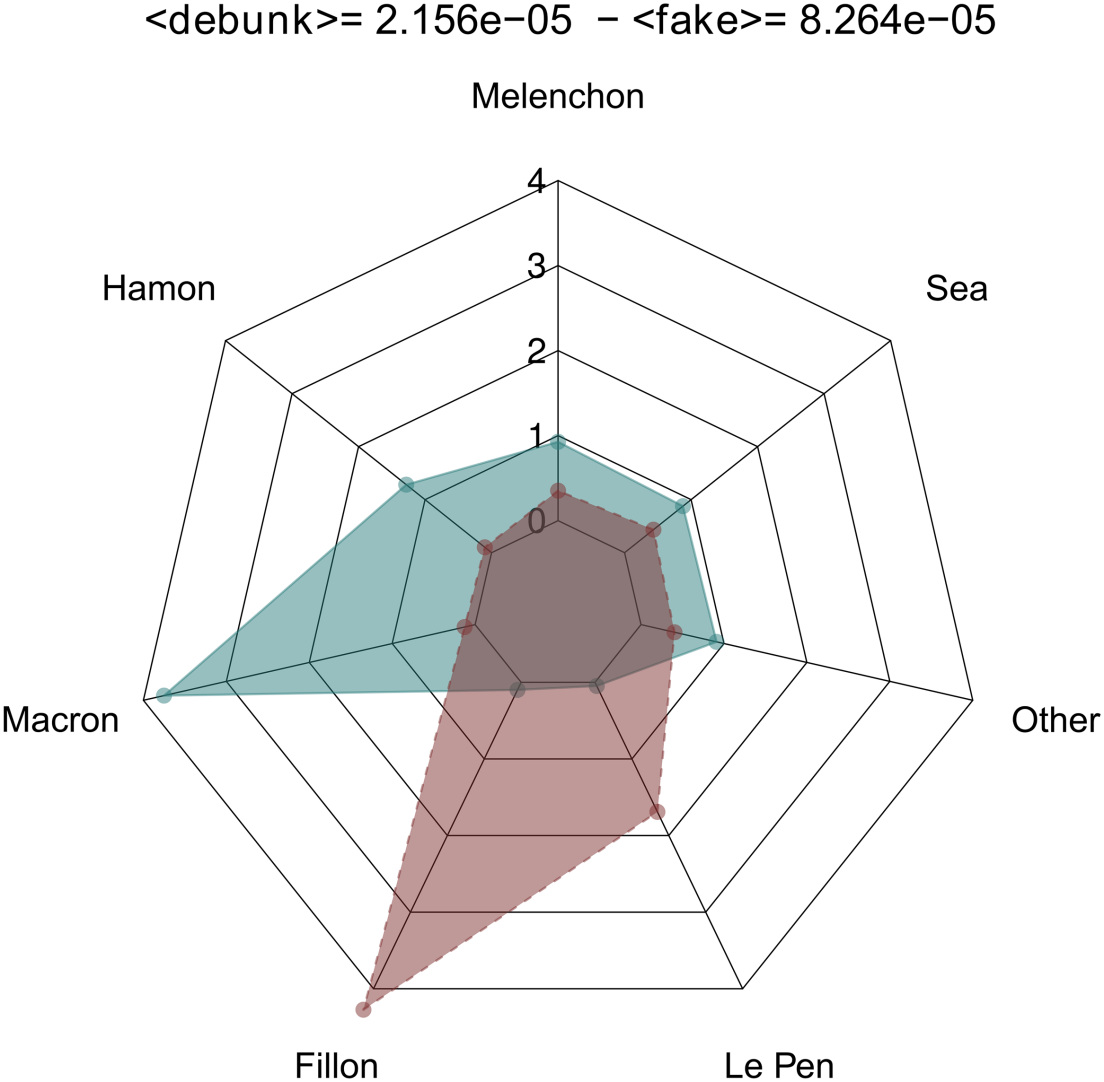
Radar chart of the normalized propensity to spread fake news (dotted red) and debunks (blue) per community. 1 means a spread at average level.

To compare the fake news and debunks sharing propensities, Fig 13 shows the normalized propensity of each community to share fake news and debunks (a propensity of 1 is the average). The Macron community shared almost four times more debunks than the average, while the Fillon community shared more than four times more fake news than the average.

This preliminary study depicts a “split-brain” political landscape from the perspective of fake news: one “hemisphere” produces or spreads fake news, the other produces or spreads debunks. This dissociation between the audience that reads fake news and the one that reads their debunks must be taken into account in mitigation strategies for the fake news phenomenon.

Fighting fake news is a very tricky task with at least two counter-intuitive effects: the *backfire effect* [74]—repeating a myth increases familiarity, reinforcing it—and the *continued influence effect* [75]—despite a retraction, people continue to rely on misinformation. In addition, in absence of media exposure, fake news are spreading locally, from close to close. But when exposing a large audience to the debunk of a fake news, there is no guaranty that a significant proportion of this audience will not choose to trust the fake news source rather than the debunk (*e.g*. believers in conspiracy theories). Thus exposing some communities that were previously not aware of the existence of a fake news could start a new outbreak of fake news in areas of social media that were spared until then.

Unlike vaccination for which people have to be treated preventively, in the domain of fake news, people have to be debunked after exposure. In that sens, the fact that fakes news tend to spread in less communities than other news is a good news: counter measures can target only those particular communities or the media they rely on without interfering with a broader audience. The second good news, when we look closely at the figures, is that at least on Twitter, political fake news do not seems to be that much popular. Our study identified that in France, only 0.08% of political tweets contained fake news. Although it is likely that de *Decodex* failed to identify a significant proportion of fake news, this is nevertheless a quite low proportion. This estimate is also in line with Vosoughi *et al*. (2018) who identified “only” 4.5M shares of stories over 10 years of Twitter records, to be compared to an estimate of 500M tweets sent per *day* in 2017.

## Conclusions

We have demonstrated that methods involving the analysis of exchanges of political content on social networking sites such as Twitter allow automatic reconstruction of political communities which, by their semantic profile and patterns of development, provide deep insights into the dynamics at play inside the space of political activists.

By applying these methods to data from the 2017 French presidential election, we reconstructed with a high accuracy the multi-polar French political space, retrieved the main events which occurred during the campaign, and revealed the multiple reconfigurations of political communities, some of which took place under relatively confidential circumstances. By using interaction data only, our approach has the advantage of being independent from the language used during these exchanges, provided the studied social activity involves a certain level of commitment from the participants, as it is the case in politics.

After checking the accuracy of this reconstruction, we demonstrated how this approach can help to better characterize online political communities, both from the perspective of their structure and from point of view of the kind of information they propagate, fake news included:

1. Political communities can be reconstructed from Twitter data:
  - The usage of Twitter by political communities makes it possible to reconstruct the evolution of the political landscape of a country during presidential campaigns through correspondence between the different political currents and communities reconstructed algorithmically from their digital traces,
  - Official and non official key political events correspond to identifiable mathematical structures in the reconstructed political landscape like split, merge, fade-out, fade-in or abrupt change of sizes of communities,
2. This reconstruction gives insights into the structures of political communities and their dynamics (the case of France):
  - Online communities are able to reconfigure themselves in a very short period of time as a result of key events such as victories at primaries or alliances between leaders,
  - There is a generic political community structure on Twitter, comprising a core of activists who remain for several weeks within the community, a considerably more volatile peripheral membership, and an intermediate set of accounts covering a broad spectrum of participation,
  - Political communities are heterogeneous with respect to the online activity, commitment, political integration and politicization of their members suggesting both different profiles of political commitment from one community to another and distinct dynamics for recruiting new members. The characterization from digital traces of the variety of processes sustaining the cohesion of a community is an open challenge,
  - People with extreme political views tend to be the most committed to their ideology,
3. This reconstruction gives insights into the impact of communities on information diffusion and in particular on their role in the fake news phenomena (the case of France):
  - The online community structure constrains the diffusion of information among Twitters activists, most retweet cascades occur within one or two communities,
  - The French political Twitter landscape looks like a “split-brain” from the perspective of fake news: one “hemisphere” produces or spreads fake news, the other produces or spreads debunks,
  - Fake news seems to be more shared than their debunks in terms of number of accounts reached, but the phenomena is reversed at the community level: the spread of fake news tends to be confined to fewer communities than other political news. Consequently, in fake news impact assessment, the variety of communities they reach should be taken into account in addition to the number of people they reach.
  - Overall, on Twitter, for French politics, the fake news phenomena seems rather limited both in terms of volume (order of magnitude of 0.1%) and in terms of the diversity of the communities concerned. 72,9% of all fake news analyzed was produced and disseminated by only two political communities. Their diffusion is therefore above all the work of political communities, not laymens.

As a consequence of its 11-months duration, its temporal resolution (real-time capture of data, once every second), and its scale (≥2.4 million Twitter accounts of which ≥180, 000 had a sufficient degree of interaction with the *Politoscope* to be cataloged into a political community), this study establishes the basis for a series of analyzes providing unprecedented insight into opinion dynamics and the reconfiguration of political communities. It provides access to an intermediate level of resolution between sociological surveys in the field and global statistical studies (such as those conducted by national census agencies), from which we expect that, in the future, it will represent a major source of political and sociological knowledge supplementing the more traditional approaches.

We conclude this paper with a comment concerning the political and ethical implications of the development of these methods. In recent years, the boundary between private and public life has become increasingly thin, as the digital trails left by citizens have been systematically analyzed, by combining an ever increasing number of databases (purchase history, web navigation history, votes, store loyalty cards, public transport, GPS location, etc.), and by considering an increasingly significant history based on population sample sizes that tend to be of the order of magnitude of the population size itself. Marketing and the economic sector were the first areas of activity to seize these new opportunities, in the interests of financial gains. More recently, the political sphere has adopted these new opportunities and has tried to take advantage of them to gain greater insight into the preferences and political persuasion of voters.

A perfect example of this tendency can be found in the leak of sensitive personal data concerning nearly 200 million Americans, compiled for the Republican party during the Trump 2016 campaign (≈ 99% of the electorate), which occurred in July 2017 [76].

We took considerable care during the entire *Politoscope* project to anonymize the processed data and to produce aggregate data only, thus making it impossible to identify specific individuals, with the exception of a small number of well-known political figures. However, the research presented in this paper demonstrates that it is possible, using the conspicuous trails left in the public digital domain, and with relatively accessible computational means, to catalog a significant proportion of the electorate of an entire country, according to their political orientations, even though, as we have pointed out, there is still a certain margin of uncertainty. From this observation, it is important for citizens to realize that they can be accountable for any trace they leave on the web. The open platform politoscope.org is, in particular, designed to promote this awareness. We can only count on the honesty and good faith of current and future political leaders to refrain from using, as has happened so often in the past and continues to be the case in certain countries [77–80], data of this type for the purposes of discrimination, manipulation, or even repression and persecution.

## Supporting information

S1 File Exhaustive data description and complementary analyses. Table of content:
Text A: Main political events of the 2017 French presidential campaign,Text B: Data collection platform
B.1 Collection focused on main political accounts (follow API),B.2 Collection focused on political terms (track API),B.3 List of the followed political figures (track and follow API),B.4 Platform architecture,B.5 Availability of the data,
Text C: Bots detection,Text D: Lifetime of tweet and Twitter activity patterns,Text E: Community stability,Text F: Correlation between community structure and retweets patterns: confirmation of the echo chamber effect,Text G: Analysis of the communities’ semantic background
7.1 Definition of the themes through queries,7.2 Distribution of topics by political communities,7.3 Evolution of community vocabulary,
Text H: Fake news data cleaning,Text I: Vocabulary of the political discourse of the presidential election 2017,Text J: Analysis of late retweets.
(PDF)
