# Supplementary material for: Reconstruction of the socio-semantic dynamics of political activist Twitter networks—Method and application to the 2017 French presidential election
Source: PLoS One. 2018 Sep 19;13(9):e0201879. doi: 10.1371/journal.pone.0201879 (PMC6145593; doi:10.1371/journal.pone.0201879)
Supplement: S1 File — Table of content: Text A: Main political events of the 2017 French presidential campaign,Text B: Data collection platform B.1 Collection focused on main political accounts (follow API),B.2 Collection focused on political terms (track API),B.3 List of the followed political figures (track and follow API),B.4 Platform architecture,B.5 Availability of the data, Text C: Bots detection,Text D: Lifetime of tweet and Twitter activity patterns,Text E: Community stability,Text F: Correlation between community structure and retweets patterns: confirmation of the echo chamber effect,Text G: Analysis of the communities’ semantic background 7.1 Definition of the themes through queries,7.2 Distribution of topics by political communities,7.3 Evolution of community vocabulary, Text H: Fake news data cleaning,Text I: Vocabulary of the political discourse of the presidential election 2017,Text J: Analysis of late retweets. (PDF) [file pone.0201879.s001.pdf]

PLOS | ONE

Supporting Information : Reconstruction of the socio-semantic dynamics of political activist Twitter networks - Method and application to the 2017 French presidential election

Noé Gaumont[1,], Maziyar Panahi[2], David Chavalarias[1,2,*]

1 Centre d'Analyse et de Mathématique Sociales (CAMS), Centre National de la Recherche Scientifique (CNRS) / École des Hautes Études (EHESS), Paris, France
2 Institut des Systèmes Complexes Paris Île-de-France (ISC-PIF), CNRS, Paris, France

* david.chavalarias@ehess.fr

Abstract

Background Digital spaces, and in particular social networking sites, are becoming increasingly present and influential in the functioning of our democracies. In this paper, we propose an integrated methodology for the data collection, the reconstruction, the analysis and the visualization of the development of a country's political landscape from Twitter data.

Method The proposed method relies solely on the interactions between Twitter accounts and is independent of the characteristics of the shared contents such as the language of the tweets. We validate our methodology on a case study on the 2017 French presidential election (60 million Twitter exchanges between more than 2.4 million users) via two independent methods: the comparison between our automated political categorization and a human categorization based on the evaluation of a sample of 5000 profiles descriptions ; the correspondence between the reconfigurations detected in the reconstructed political landscape and key political events reported in the media. This latter validation demonstrated the ability of our approach to accurately reflect the reconfigurations at play in the off-line political scene.

Results We built on this reconstruction to give insights into the opinion dynamics and the reconfigurations of political communities at play during a presidential election. First, we propose a quantitative description and analysis of the political engagement of members of political communities. Second, we analyze the impact of political communities on information diffusion and in particular on their role in the fake news phenomena. We measure a differential *echo chamber* effect on the different types of political news (fake news, debunks, standard news) caused by the community structure and emphasize the importance of addressing the meso-structures of political networks in understanding the fake news phenomena.

Conclusions Giving access to an intermediate level, between sociological surveys in the field and large statistical studies (such as those conducted by national or international organizations) we demonstrate that social networks data make it possible to qualify and quantify the activity of political communities in a multi-polar political environment ; as well as their temporal evolution and reconfiguration, their structure, their alliance strategies and their semantic particularities during a presidential campaign through the analysis of their digital traces. We conclude this paper with a comment on the political and ethical implications of the use of social networks data in politics. We stress the importance of developing social macroscopes that will enable citizens to better understand how they collectively make society and propose as example the "*Politoscope*", a macroscope that delivers some of our results in an interactive way.

Contents

A Main political events of the 2017 French presidential campaign **4**

B Data collection platform **7**
- B.1 **Collection focused on main political accounts (follow API)** 7
- B.2 **Collection focused on political terms (track API)** 7
- B.3 **List of the followed political figures (track and follow API)** 9
- B.4 **Platform architecture** 12
- B.5 Availability of the data 13

C Bots detection **15**

D Lifetime of tweet and Twitter activity patterns **17**

E Community stability **17**

F Correlation between community structure and retweets patterns: a confirmation of the echo chamber effect **19**

G Analysis of the communities' semantic background **21**
- G.1 **Definition of the themes through queries** 21
- G.2 **Distribution of topics by political communities** 23
- G.3 **Evolution of community vocabulary** 24

H Fake news data cleaning **30**

I Vocabulary of the political discourse of the presidential election 2017 **31**

J Analysis of late retweets **44**

K List of figures and tables **52**

List of additional files available on Harvard Dataverse

The persistent URL of these files is:
https://dataverse.harvard.edu/dataset.xhtml?persistentId=doi%3A10.7910%2FDVN%2FAOGUIA

- **A1 File - Graph_August_December.gexf:** Anonymized graph of the 5 communities between August and December 2016. The user ids are anonymized but the labels of main political figures are displayed.
- **A2 File - 2017 French Presidential Elections Programs.csv:** Political measures taken from the campaign programs of the candidates for the 2017 French presidential election.
- **A3 File - French_Presidential_Election_2017 Topics.gexf:** Topics maps (co-word analysis with confidence proximity) from the analysis of the programs of the candidates to the 2017 French presidential election.
- **A4 File -Politoscope_RetweetGraph_2017-04-09_2017-04-23_min_3_anon.tab:** Anonymized graph for the French political landscape (3-communities) over the period [9 April 2017 – 23 April 2017] (on the day of the first round of elections).
- **A5 File - Politoscope_RetweetGraph_2017-04-24_2017-05-08_min_3_anon.tab :** Anonymized graph for the French political landscape (3-communities) over the period [April 24 2017 – May 8 2017] (on the day of the second round of elections).
- **A6 File - hoax_lines.json** List of fake news made by *Les Decodeurs* (https://github.com/multivacplatform/presidentielle2017-info). All links have been manually screened to remove links that were judged openly parodic.
- **A7 File - debunk_lines.json:** List of debunks made by *Les Decodeurs*(https://github.com/multivacplatform/presidentielle2017-info).
- **A8 File - topic_queries.txt:** Queries used to define major topics of the 2017 French presidential election (https://github.com/multivacplatform/presidentielle2017-info)
- **A9 File - keywords-query.txt:** Keywords extracted from the programs of the 2017 French presidential election and a sample of tweets from the candidates. (https://github.com/multivacplatform/presidentielle2017-info).

The List of all the tweet IDs collected by the Politoscope.org platform between Aug. 1st 2016 to May 8 2017 (**A10 File** - tweet_id.tar.xz) is available at
https://dataverse.harvard.edu/dataset.xhtml?persistentId=doi:10.7910/DVN/6739SP

A Main political events of the 2017 French presidential campaign

In the spring of 2016, many anticipated a left-right alternation with Alain Juppé being the favorite of all polls. But the 2017 French presidential election were exceptionally eventful with many major upheavals in very short periods and the collapse of the two main candidates (Fillon and Hamon) and parties that had dominated the political scene for more than forty years. Here we present the main milestones of the campaign. Two major international events also took place concurrently, which surprised all analysts and opened possibilities for future elections: Brexit and the election of Donald Trump.

- **2017/06/12:** Interview with Juppé on TF1 deploring his nickname "Ali Juppé" (a defamatory action launched by the extreme right and then taken up by some members of the Sarkozy and Fillon communities during the right-wing primary),
- **2017/06/23:** The United Kingdom votes Brexit,
- **2016/07/14:** Terrorist attack in Nice (86 people killed and 458 injured),
- **2016/08/28:** François Fillon, a candidate for the right-wing primary, attacked his rival Nicolas Sarkozy by asking *"Who imagines for a single moment the General de Gaulle put under indictment?"*. This sentence will be repeated several times during the Penelope Gate when Fillon himself was indicted,
- **2016/08/30:** Macron leaves the government as Minister of Economy and Finance,
- **2016/10/13:** 1st debate of the right-wing primary, Sarkozy and Juppé are the favorites of the polls,
- **2016/11/08:** Election of Donald Trump ,
- **2016/11/03:** 2nd debate of the right-wing primary,
- **2016/11/16:** Macron announces his candidacy announcement,
- **2016/11/17:** 3rd debate of the right-wing primary,
- **2016/11/20:** First round of the right-wing primary lead by Fillon and Juppé,
- **2016/11/27:** Second round of the right-wing primary won by Fillon,
- **2016/12/01:** The incumbent president François Hollande announces that he would not stand for a second term in office,
- **2017/01/12:** 1st debate of the left-wing primary,
- **2017/01/15:** 2d debate of the left-wing primary,
- **2017/01/17:** Frictions among *les Républicains* after disputed legislative investitures, including that of Nathalie Kosciusko-Morizet (NKM), to whom Fillon offered an easy-to-win district,

- **2017/01/19:** 3rd debate of the left-wing primary. Marine Le Pen, president of the *Front National*, is credited with 25% to 26% of voting intentions in the first round, according to an Ipsos poll published by *Le Monde*. As for Emmanuel Macron, he is close to François Fillon who becomes less popular in the polls,
- **2017/01/20:** Trump's presidential inauguration. Attack of Rachida Dati (ex. former Minister of Justice of Sarkozy) against NKM investiture,
- **2017/01/21:** Maurice Leroy (UDI and support of Sarkozy during the primary) warns François Fillon,
- **2017/01/22:** First round of the left-wing primary lead by Hamon and Valls,
- **2017/01/25:** The *Canard Enchaîné* unveils the first piece of information on the *Peneloppe Gate*, which raises suspicions of misappropriation of public funds by Fillon,
- **2017/01/29:** Second round of the left-wing primary won by Hamon,
- **2017/02/21:** Fillon announces its health plan, which differs from previous statements,
- **2017/02/22:** Announcement of Le Pen summon by the anti-corruption office ; she will not attend the summons by the judge,
- **2017/02/23:** Bayrou rallies to Macron,
- **2017/02/26:** Jadot rallies to Hamon,
- **2017/03/01:** Summon of Fillon for an indictment ; several leader of *les Républicains* call for another candidate. Juppé is considered as a possible replacement for Fillon,
- **2017/03/02:** Publication of the political program of *En Marche !*,
- **2017/03/03:** Summon of Le Pen for a possible indictment (parliamentary assistants),
- **2017/03/05:** François Fillon organizes a rally in Trocadéro (Paris). He announces 200 000 attendees (including leading supports of Sarkozy) and presented this as a support to the upholding of his candidacy,
- **2017/03/06:** Juppé publicly renounces all prospect of presidential candidacy. Confirmation of the legitimacy of Fillon by the political bureau of *les Républicains*, which was originally convened to ask that he be dismissed,
- **2017/03/20:** Publication of the official list of candidates by the Constitutional Council,
- **2017/03/20:** Debate on TF1 between five *big* candidates (Mélenchon, Hamon, Macron, Fillon and Le Pen),
- **2017/03/29:** Valls announces that he will vote for Macron,
- **2017/04/04:** Debate on BFMTV with all 11 candidates. Poutou stands out with his commentaries on Le Pen and Fillon judicial troubles,
- **2017/04/16:** European Parliament committee could convene Marine Le Pen before second round of presidential elections,

- **2017/04/20:** Champs Élysées terrorist attack during the political show on France 2 in which all candidates were invited to speak. Le Pen radicalises her discourse with a polemic: "colonization has brought a lot [of benefit], especially to Algeria",
- **2017/04/23:** First round of the presidential election lead by Macron and Le Pen. Mélenchon refuses to give voting instruction to his supports, in contradiction to what he did in 2002 (he called to vote against the *Front national*),
- **2017/04/28:** Dupont-Aignan rallies to Le Pen,
- **2017/05/03:** Le Pen's counter-performance during the Macron-Le Pen TV debate between the two rounds.
- **2017/05/07:** Second round of the presidential election won by Macron.

B Data collection platform

As we aim to analyze how the political space organized during the 2017 French presidential election, we chose to collect data from Twitter and by two complementary methods.

B.1 Collection focused on main political accounts (follow API)

We established a list of more than 3 700 accounts of French political figures (candidates, deputies, senators, persons with a significant party mandate). We gather informations from these accounts with the follow API of Twitter (see the inset).

The documentation describes exactly the data collected for a followed user account:

From Twitter ***follow*** *API documentation*[a]. For each user specified, the stream will contain:

- Tweets created by the user.
- Tweets which are retweeted by the user.
- Replies to any Tweet created by the user.
- Retweets of any Tweet created by the user.
- Manual replies, created without pressing a reply button (e.g. "@twitterapi I agree").

The stream will not contain:

- Tweets mentioning the user (e.g. "Hello @twitterapi!").
- Manual Retweets created without pressing a Retweet button (e.g. "RT @twitter-api The API is great").
- Tweets by protected users.

[a]https://dev.twitter.com/streaming/overview/request-parameters

Given an account X, a content A and $T(X, A)$ a tweet published by X containing A, we collected, within the Twitter API volume limit, for each followed account X and any account Y the tweets $T(X, A)$, $T(Y, T(X, A))$ (*e.g.* retweet), and if A is a response to X("@X ..."), all $T(Y, A)$ (see the Fig. A).

B.2 Collection focused on political terms (track API)

The collection of tweets from accounts that are not followed is limited by the follow API limits. To overcome this limitation and get the tweets that cite an important candidate in text, we have compiled a list of candidate related terms (screenname, party name, etc.) and used the track API, which allows to capture all tweets mentioning a given expression (see the inset). Several hashtags have been created specifically for the presidential election to categorize the tweets relevant for this event. We have identified most of them and added them to the list of

expressions followed in order to collect tweets mentioning them (*e.g;* #legranddebat [1,360,502 mentions], #2017ledebat [761,400 mentions]).

> *From Twitter* ***track*** *API documentation*[a]*.*
> A comma-separated list of phrases which will be used to determine what Tweets will be delivered on the stream. A phrase may be one or more terms separated by spaces, and a phrase will match if all of the terms in the phrase are present in the Tweet, regardless of order and ignoring case. By this model, you can think of commas as logical ORs, while spaces are equivalent to logical ANDs (e.g. 'the twitter' is the AND twitter, and 'the,twitter' is the OR twitter).
> The text of the Tweet and some entity fields are considered for matches. Specifically, the text attribute of the Tweet, expanded_url and display_url for links and media, text for hashtags, and screen_name for user mentions are checked for matches.
>
> [a]https://dev.twitter.com/streaming/overview/request-parameters

This is the list of terms followed with track API:

- presidentielle2017
- présidentielle2017
- #presidentielle2017
- primaires2016
- loitravail
- frexit
- Nuit Debout
- nuitdebout
- #nuitdebout
- #Fillon
- #Macron
- #PenelopeGate
- #Hamon2017
- #Valls2017
- #Montebourg2017
- #Marine2017
- #Fillon2017
- #Peillon2017
- #Asselineau2017
- #Macron2017
- #Marinelepen2017
- #Mlp2017 MLP_officiel
- FrancoisFillon
- fhollande
- manuelvalls
- alainjuppe
- nk_m
- JLMelenchon
- NicolasSarkozy
- benoithamon
- EmmanuelMacron
- yjadot
- n_arthaud
- montebourg
- UPR_Asselineau
- jfpoisson78
- JLM2017
- #NDA2017
- dupontaignan
- jeanlassalle
- PhilippePoutou
- n_arthaud
- manuelvalls
- #legranddebat
- presidentielles2017
- présidentielles2017
- présidentielle
- presidentielle
- jevote
- avote
- avoté
- sansmoiLe7mai
- Debat2017
- 2017LeDebat
- jamaismacron
- jamaislepen
- jamaismarine
- jamaislefn
- JeVoteMarine
- JeVoteMacron
- ChoisirLaFrance
- Dupont-Aignan
- enmarche
- frontnational
- #mfga

The Twitter API is, in its free version, only a sample of the Twitter traffic. We estimated the proportion of retweet captured by comparing the amount of retweets actually captured by our platform to the number of retweets reported by Twitter in the meta-data stored in the last retweet.

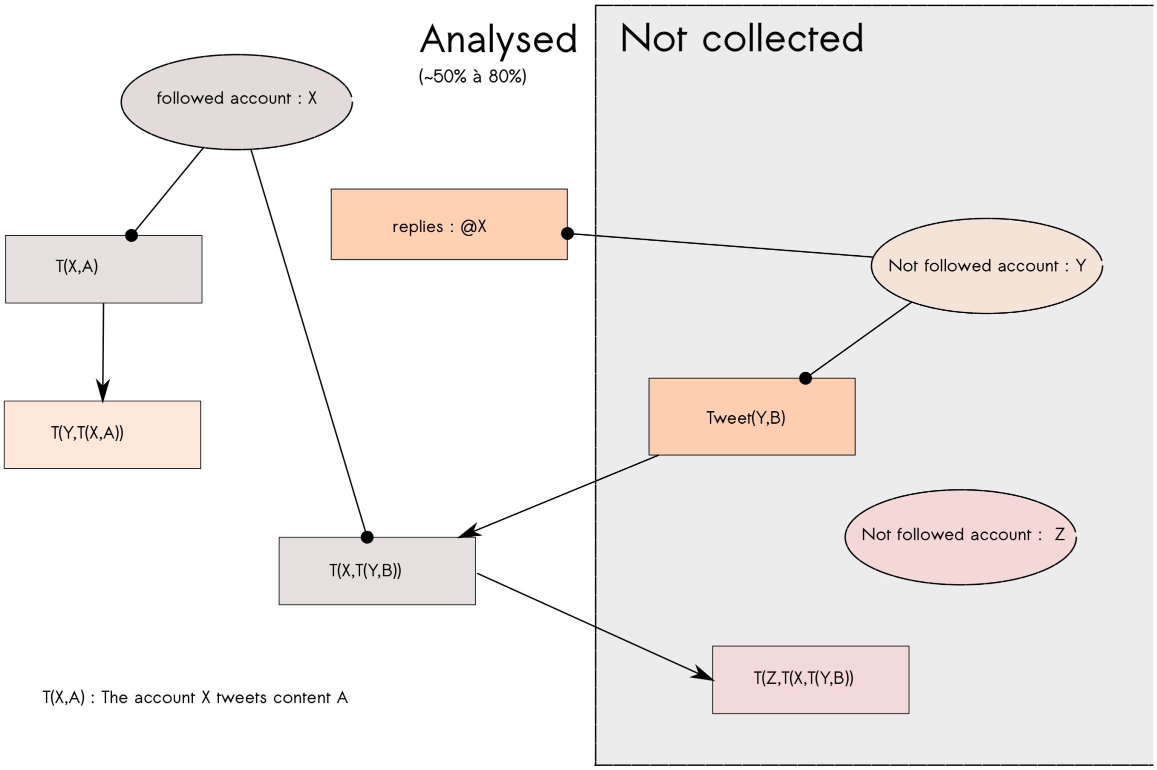

Fig A. Description of the capture protocol centred on political leaders. This is complemented by a keyword-centric capture protocol (Twitter track API).

For each day t and each original tweet Tw in this day, we denote the number of retweets captured by $n_t^c(Tw)$ and by $n_t^m(Tw)$ the maximum number of retweets reported by Twitter. We define the proportion of captured retweets by:

$$\nu(t) = \frac{\sum_{\{Tw\}} n_t^c(Tw)}{\sum_{\{Tw\}} n_t^m(Tw)}$$

Fig. B presents the evolution of the daily proportion of captured retweets as well as the total number of Tweet captured. This proportion is variable through the days and depends on Twitter in an unspecified way. On average, we captured 34% of retweets.

As Twitter does not publish the code for its API, we do not know the sampling process used by the API. In particular, we don't know if the number of retweets mentioned in the meta-data is one the feature used to select which retweet is sampled. Nevertheless, we can reasonably believe that the proportion of tweets captured (mention, quotations, etc.) by our platform is in the order of 34% of the total tweets corresponding to our capture criteria.

However, our results show that this sampling uncertainty imposed by the Twitter APIs is not a hindrance to analysing the dynamics of political communities.

B.3 List of the followed political figures (track and follow API)

For the French presidential election, two primaries have been organized by the two main political parties: the *Parti Socialiste* (left) and *Les Républicains* (right). Data on the

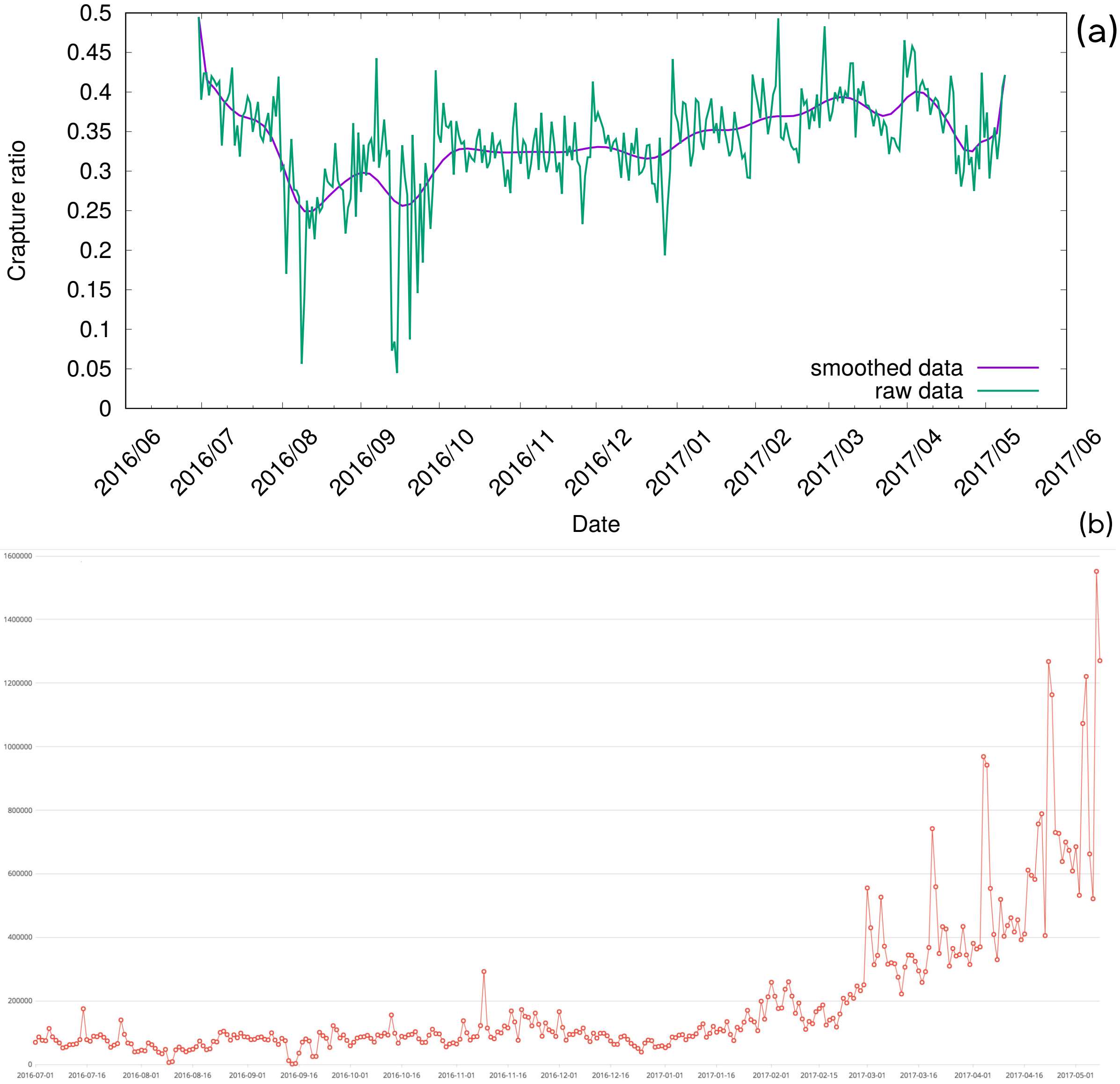

Fig B. Statistics on retweets capture volumes *(a) Up:* Evolution of retweet capture rate over time. Some low points correspond to server failures. *(b) Bottom:* Daily number of tweets collected.

presidential election candidates and the main candidates of the primaries were collected through the *track* and *follow* API (for the ~ 3700 other political figures, data were collected only through the follow API) :

Presidential election candidates (sorted by score)

1. Emmanuel Macron (EM)
2. Marine Le Pen (FN)
3. François Fillon (LR)
4. Jean-Luc Mélenchon (FI)
5. Benoît Hamon (PS)
6. Nicolas Dupont-Aignan (DF) (UPR)
7. Jean Lassalle
8. Philippe Poutou (NPA)
9. François Asselineau
10. Nathalie Arthaud (LO)

Main candidates of the primaries

1. Yves Jadot (EEV - rallied to Hamon)
2. Emmanuel Valls (former prime minister and candidate to the left-wing primary)
3. Arnaud Montebourg (former minister and candidate to the left primary)
4. Vincent Peillon (former minister and candidate to the left-wing primary)
5. François Hollande (Outgoing president)
6. Alain Juppé (former primer minister, poll favorite for the right-wing primary)
7. Nathalie Kosciusko-Morizet (former minister and candidate to the right-wing primary)
8. Nicolas Sarkozy (Former president and candidate to the right-wing primary)
9. Jean-Frédéric Poisson (candidate to the right-wing primary)

B.4 Platform architecture

The indexation of political tweets was started on June 1st 2016 using the CNRS/ISC-PIF Multivac infrastructure (https://multivac.iscpif.fr see Fig. C). It operated continuously until the day after the presidential election, with the exception of rare interruptions for maintenance of our servers (between 13-09-2016 and 15-09-2016 and between 21-09-2016 and 22-09-2016). Between June 1st 2016 and May 7 2017 (the second round of the presidential election), we collected 60 326 216 tweets created by 2 477 910 unique accounts.

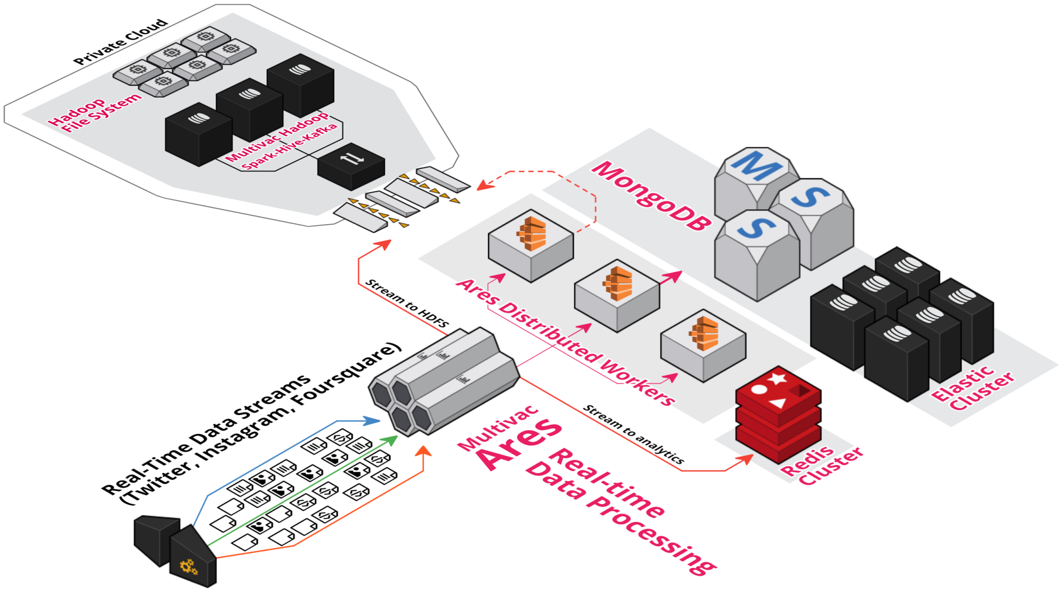

Fig C. Synthetic description of the Multivac infrastructure used to set up the real-time analysis of the tweets. This infrastructure leverages technologies such as Elasticsearch, Apache Hadoop, RabbitMQ, MongoDB and Redis.

TWITTER DATA ANALYTICS ARCHITECTURE (see also Fig. C)
Real-time data processing and micro services

- Elasticsearch Cluster (highly available and distributed)
  - Primary database with over 6 billion indexed documents
  - Full-text search
  - Indexing keywords
  - Data aggreagation (date histogram, significant, terms, nested, etc.)
- MongoDB
  - Secondary database with over 12 billions of data
- RabbitMQ
  - Distributing Tweets to workers for parallel computations
- Node.js
  - Streaming Tweets by tracking and following Twitter accounts
  - Scalable workers: annotating and tagging Tweets in realtime
  - Extracting Tweets content
- Redis
  - Caching
  - In-memory analysis

Offline and batch processing: Hadoop cluster

- HDFS (Hadoop distributed file system)
- Apache Hadoop YARN: Managing clsuter resources
- Apache kafka: Streaming data for batch processing
- Apache Spark
  - ETL
  - Pre-processing data
- Apache Hive: Run SQL queries in Hadoop

B.5 Availability of the data

Data was collected according to Twitter's terms and according to these terms, *"If you provide Content to third parties, including downloadable datasets of Content or an API that returns Content, you will only distribute or allow download of Tweet IDs, Direct Message IDs, and/or User IDs."* (cf. https://developer.twitter.com/en/developer-terms/agreement-and-policy#id34)

Consequently, for the Twitter raw data, we had to restrict the publication of the data to the Tweet Ids with a script that makes it possible to download from Twitter the full content of the tweets less the deleted tweets. All the necessary material is available at the Harvard Dataverse (A10 File) doi:10.7910/DVN/6739SP [1]. It is thus makes it possible to reconstitute the complete Twitter database used for this study, less the deleted tweets.

As mentionned by Twitter's TOFU (https://developer.twitter.com/en/developer-terms/policy) these data are made available under some restrictions, which are : *"You may not distribute more than 1,500,000 Tweet IDs to any*

entity (inclusive of multiple individual users associated with a single entity) within any given 30 day period, unless you are doing so on behalf of an academic institution and for the sole purpose of non-commercial research or you have received the express written permission of Twitter."

To facilitate the reuse of the data, we have also built an API based infrastructure available at https://api.iscpif.fr/docs/#/datapol. On request and for academic use only, this infrastructure gives access to the aggregated data that Twitter allows to redistribute.

C Bots detection

Robots or *bots* are often used to control a Twitter account and increase its on-line presence. If this usage were too massive, it could prevent us to analyze the behavior of political activists. To ensure that the proportion of bots animated accounts is at a reasonable level, we use the BotOrNot API to detect accounts that might not be animated by "real" users.

We created 2 samples of about 50,000 account each. The first one contains all 49,995 accounts that were at least once in a community while having at least 3 links in the retweet graph. This sample represents people integrated in the retweet graph. The second sample contains 50,000 users that were at least once in a community. This sample represents random accounts in the communities.

The API returns for each account its probability to be a bot. With the same threshold of 0.5 used by the API designer to classify an account as a bot, there are around 5.3% of the accounts that belonged to a community that are bots. These accounts produced less than 5% of the retweets registered in the communities (see Tables A and B).

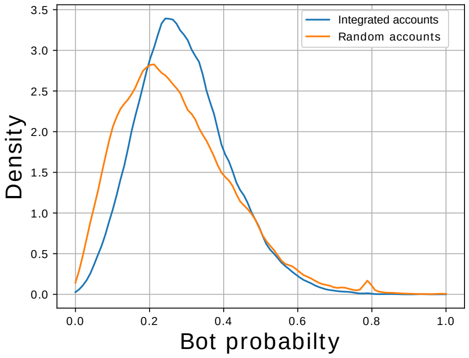

Fig D. Distribution of bot probability. Blue curve: accounts that have been at least once in a community while having at least 3 links in the retweet graph. Orange curve: random accounts that have been at least once in a community. This detection was carried out in June 2016.

Name	$< accounts >$	$< bots >$	% of bot
François Asselineau	213	14	6.5 %
François Fillon	1340	68	5.1 %
Benoit Hamon	561	31	5.6 %
Marine Le Pen	1560	70	4.5 %
Emmanuel Macron	994	44	4.4 %
Jean-Luc Mélenchon	804	30	3.7 %

Table A. Bot presence in communities. Average number of accounts per week and per community ($< accounts >$) as well as the average number of bots detected per week per community($< bots >$).

Tweet type	Political bots	Random bots
Original	7.2%	7.4%
Retweet	4.8%	3.7%
Response	0.9%	1.8%
Quote	3.4%	1.5%
Total	4.7%	3.9%
**Account proportion**	**5.1%**	**6.8%**

Table B. Proportion of tweets posted by bots among political communities and by bots among randomly selected accounts. For example, bots in political communities emitted 7.2% of the original tweets issued by all accounts being in a political community.

D Lifetime of tweet and Twitter activity patterns

Political activity on Twitter has a certain regularity which is the coupling between an endogenous activity (day/night, week/w.e.) and exogenous activity that depends on current political events. The average over the whole corpus clearly shows a weekly regularity (see Fig. E).

Regarding the typical retweet time, 89.005% of the retweets take place within 24h after the release of the original tweet and 98.977% take place within 14 days.

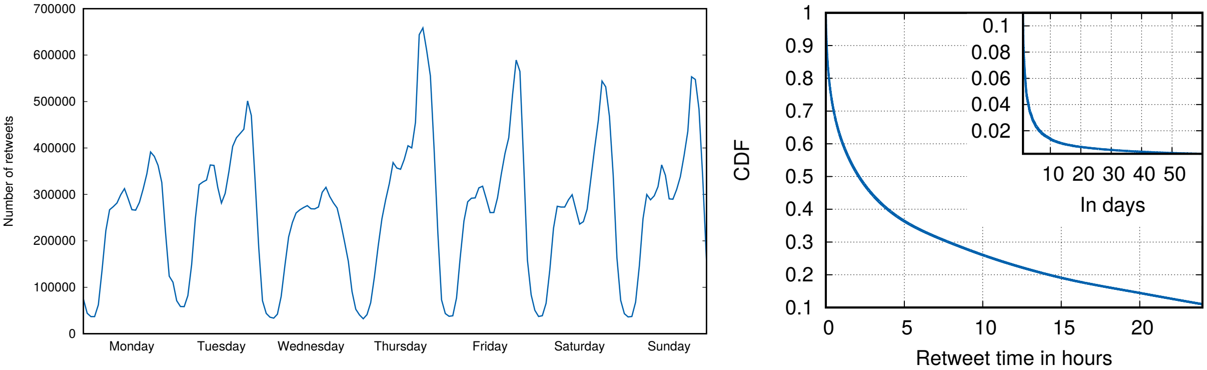

Fig E. Twitter activity patterns. *On the left:* Number of retweets observed per day and hour between 1 July 2016 and 2 May 2016; *on the right*: Cumulative distribution of retweet time.

E Community stability

Name	# people present	Average time in days
Marine Le Pen	35,812	33.8
François Fillon	30,516	30.7
Jean-Luc Mélenchon	42,206	21.8
Emmanuel Macron	57,651	16.2
Benoît Hamon	37,748	17.7
François Asselineau	14,774	11.8
Philippe Poutou	51,908	11.6
Nicolas Dupont-Aignan	30 366	5.6
Nathalie Arthaud	45,450	4.5
Jacque Cheminade	29,153	4.4
Global	187,619	55.8

Table C. Accounts statistics per community. Table showing, for each candidate, the number of accounts present at least once in his/her community inside the community over the period July 1st 2016 - May 23th 2017. The communities of Le Pen and Fillon are clearly different to the others, in the sense that they are the most stable with a very high average time. The fact that the average time spent by an account within a community (last line) is very high indicates that a significant proportion of accounts change from one community to another, without disappearing from Twitter.

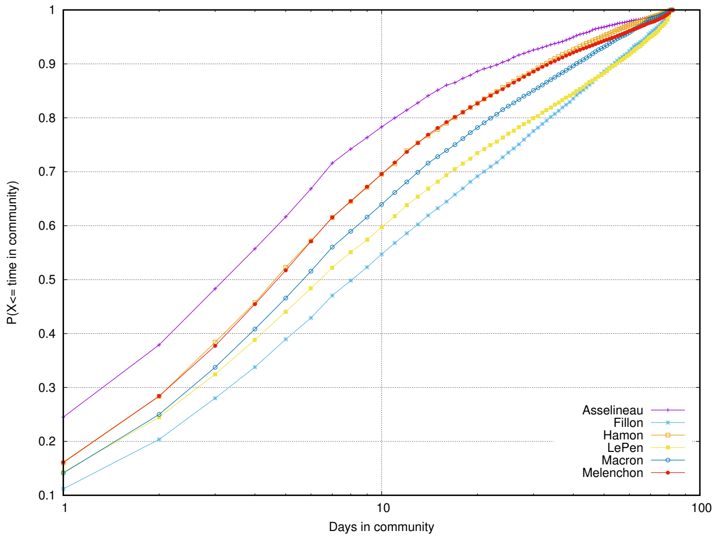

Fig F. Cumulative distribution of time spent in a community between July and the 1st round.

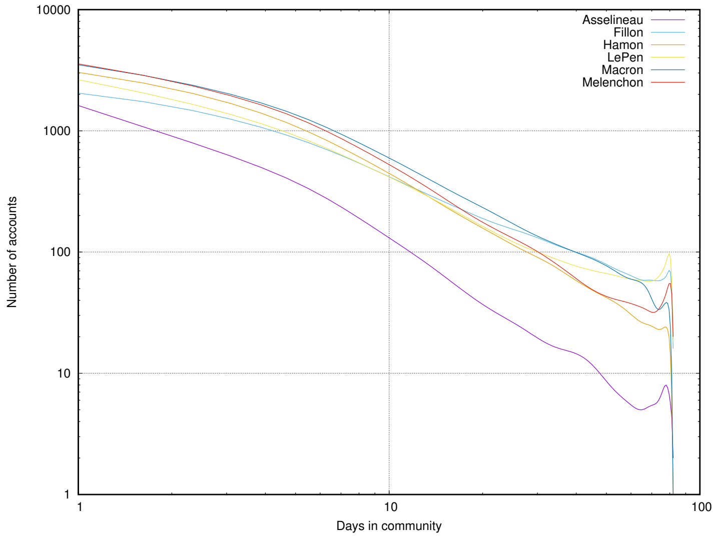

Fig G. Smooth distribution of time spent in a community between July and the 1st round.

F Correlation between community structure and retweets patterns: a confirmation of the echo chamber effect

We analysed the detail of the retweets patterns to validate the H_0 hypothesis, intuitive according to our reconstruction method, that the diffusion of retweets is strongly constrained by the calculated community structure. We checked that the retweets motives by communities are very different from what they would be if they were randomly distributed across communities by analysing the diffusion patterns for tweets with a sufficient number of retweets (≥ 50). Through our device, we have captured $4,124,888$ original tweets that totaled $39,284,569$ retweets. Of these 4 million tweets, only $118,967$ had 50 retweets or more but they totalled $21,288,537$ of retweets (54% of all retweets). By requiring that retweets come from accounts affiliated to a community, we get $82,112$ original tweets totaling $12,076,600$ retweets (31% of all retweets). Between June 1st 2016 and May 7th 2017, we processed for each day j, the N^j political communities provided by our methodology, $\mathcal{C}^j = \{C_i^j\}_{1 \leq i \leq N^j}$, and created a sub-corpus of original tweets $\mathcal{D}_5^j0$ composed of tweets that have been retweeted at least 50 times by people whose communities are known on the day j. The communities of retweets occuring after the day j are the ones knowns on the day j, thus the community detection and the diffusion process are calculated on independent dataset. This differs from our approach in the rest of the paper in which the community computed each day is used.

Over this period, we calculated the number q_i^j of retweets from $\mathcal{D}_{50}^j$ produced by all the C_i^j. We get the distribution $\mathcal{Q}^j = \{q_i^j\}_{1 \leq i \leq N^j}$ of the retweets from $\mathcal{D}_{50}^j$ by the C_i^j communities. From this, we process the χ^2 distance between this distribution and the distribution of retweets $\mathcal{P}^j(tw) = \{p(tw)_i^j\}_{1 \leq i \leq N^j}$ from each tweets tw from $\mathcal{D}_{50}^j$:

$$\chi^2(P(tw), Q) = \sum_i \frac{(P(tw)_i - Q_i)^2}{Q_i}$$

Based on this analysis over all periods, H_0 is true for 92% of diffusions with a p-value less than 10^{-16}. Considering a threshold of p-value of 1%, 99.8% of tweets emitted by communities have a very different retweets diffusion pattern compared to a random diffusion in the communities.

By analyzing a sub-corpus of tweets that were retweeted at least 50 times by the communities[1], we have shown that over the full set of time periods, in the case of 92% of propagations, H_0 is true with a $p - value$ of less than 10^{-16}. By considering a p-value threshold of 1%, 99.8% of tweets sent by the communities have a retweet propagation that is very different to that of random propagation within communities.

This validates the intuitive hypothesis H_0: *the propagation of retweets is strongly constrained by the structure of the computed communities.*

In order to have a more accurate view of the manner in which the communities structure this propagation, we computed, for each day j and each tweet $tw \in \mathcal{D}_{50}^j$, the entropy $\mathcal{H}$ of its propagation as a function of the communities $\mathcal{C}^j$, as well as, for the purposes of comparison, the entropy of propagation throughout the full set $\mathcal{D}_{50}^j$: $\mathcal{H}(P^j(tw)) = -\sum_i P^j(tw)_i \; log(P^j(tw)_i)$.

As shown in Fig. H, the entropy of the retweet distribution within the communities is very weak, with a mean value less than 1, which indicates that most retweets of a retweet cascade

[1]These tweets captured 31% of all retweets.

occur within one or two communities[2] whereas for the full corpus of tweets, the distribution of retweets covers a much greater set of communities (mean entropy [3] of 2.707, corresponding to propagation in 6.532 communities).

The analysis of retweet propagation thus shows that the vast majority of tweets are retweeted within their original community, as for example in the case of the tweet of François Fillon shown in Fig. H : "*A Rexecode study has shown that my program would allow 1.5 million jobs to be created. This is the program that we should implement.*" (779 retweets, entropy=0.245).

We thus observe the role of *echo chambers* played by political communities [2], which structure the propagation of information with, as in the case of our study, a multi-polar configuration.

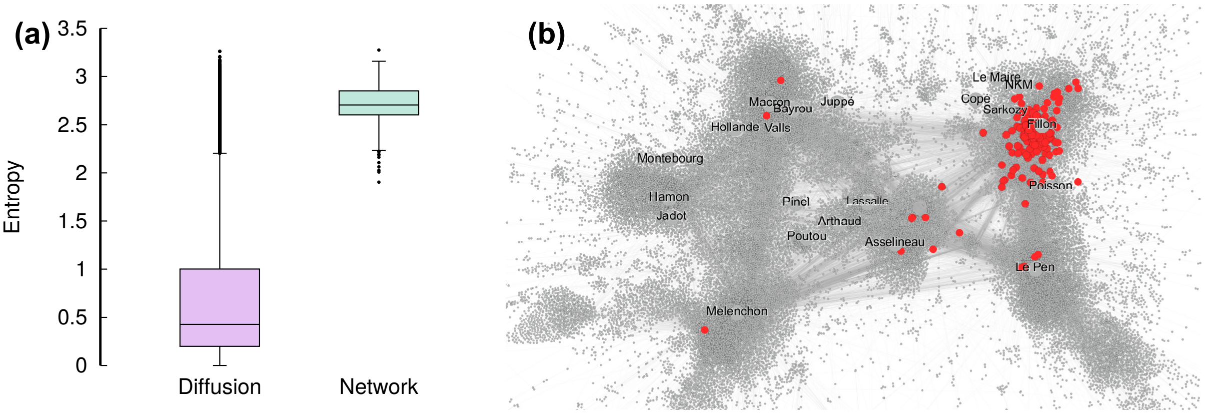

Fig H. Tweet propagation patterns. *(a) left:* boxplot of the propagation entropy of each tweet within the communities, and within the entire network ; *(b) right:* a tweet from François Fillon "*A Rexecode study has shown that my program would allow 1.5 million jobs to be created. This is the program that we should implement.*" (779 retweets, entropy=0.245) and its preferred propagation within certain communities (the reference map is Fig. 4 of the paper).

[2] As an example, if a tweet propagates in just one community, then the entropy is 0. If it propagates uniformly in 4 communities, then the entropy is 2.

[3] There is a difference between the mean entropy and the entropy for all retweets, reported in table 8 of the paper, because mean entropy for each day and entropy on the whole period are not equivalent.

G Analysis of the communities' semantic background

G.1 Definition of the themes through queries

With the help of the maps generated from the 2017 presidential programs and the Twitter corpora, we selected eleven themes that we identified as particularly important and representative of the political debates. These themes are groups of terms identified with *Gargantext* in the 2017 presidential programs (Fig. 3 of the paper), to which we retrieved overly polysemic terms susceptible to retrieve false negative in a search engine query ; and added specific keywords identified in the Twitter corpora such as *frexit*, *muzz* or *#cyberthreats*. Our objective was not to be exhaustive but to highlight the heterogeneity of attention of the political communities toward themes and their respective specializations on certain major themes. The objective was to obtain a query able to extract from the *Politoscope* database a set of coherent tweets related to this theme. A csv version of this list is provided by A8 File.

By way of example, the translation of the first query defining *employment* is:

- **Employment:** worker * OR "employment" OR "apprenticeship" OR "unemployment" OR unemployed OR "rsa" OR "smic" OR "flexisecurity" OR "vocational training" OR "professional integration" OR " vocational school" OR "initial training" OR "overtime hours" OR "part-time work" OR "professionalisation contract" OR "work conditions" OR "precarious jobs" OR "vocational and technological education" OR "vocational and technological education" OR "wage compensation" OR "39 hours" OR "social security contributions" OR "employers" OR "vocational training" OR "35 hours" OR "tutoring" OR "Pole employment" OR "entrepreneurship" OR "hourly wage" OR "wage negotiation" OR "social contribution exemptions" OR "integration of young people" OR "career path" OR "dismissal" OR "compulsory work" OR "37 hours" OR "contrat de génération" OR "loi travail" OR "increase salary" OR "bonus for employment" OR "labour code" OR "training of government officials" OR "social dialogue" OR "salary of government officials" OR "law El Khomri" OR "subcontractors" OR "become entrepreneurs" OR "suffering at work" OR "freeze of wages" OR "obligation for them to accept a job" OR "control of the wage mass" OR "no replacement of one government official in two" OR "job suppression" OR "handicraft" OR "reduction of working time" OR "RTT" OR "cice" OR "occupational mobility" OR "legal retirement age" OR "solidary retirement" OR "solidary pension" OR "hardship" OR "average salary" OR "cdi" OR "cdd" OR "relocation" OR" "universal income" OR "revenuuniversel" OR "employment contract" OR "unionisation" OR "social security contributions" OR "posting of workers" OR "posted workers" OR "delocalization" OR "relocation" OR "social plans"

The eleven themes were defined by the following queries:

- ***Employment*:** travailleur* OR emploi* OR "apprentissage" OR "chômage" OR chômeur* OR "rsa" OR "smic" OR "flexisécurité" OR "formation professionnelle"OR "insertion professionnelle" OR "lycées professionnels" OR "formation initiale" OR "heures supplémentaires" OR "temps partiel" OR "contrat de professionnalisation" OR "conditions de travail" OR "emplois précaires" OR "enseignement professionnel et technologique" OR "compensation salariale" OR "39 heures" OR "charges sociales salariales" OR "employeurs" OR "filières professionnelles" OR "35 heures" OR "tutorat " OR "pôle emploi" OR "création d'entreprise" OR "salaire horaire" OR "négociation salariale" OR "exonérations de cotisation sociale" OR "insertion des jeunes" OR "parcours professionnels" OR "licenciement*" OR "travail obligatoires" OR "jeune en alternance" OR "37 heures" OR "contrat de génération" OR "loi travail" OR "augmenter le salaire" OR "prime pour l'emploi" OR "code du travail" OR "formation des fonctionnaires" OR "dialogue social" OR "salaire des fonctionnaires" OR "loi el khomri" OR "sous-traitants" OR "devenir entrepreneurs" OR "souffrance au travail" OR "gel des traitements" OR "obligation pour eux d'accepter un emploi" OR "maitrise de la masse salariale" OR "non remplacement de un fonctionnaire sur deux" OR "suppressions de postes" OR "artisanat" OR "réduction du temps de travail" OR "RTT" OR "cice" OR "mobilité professionnelle" OR "âge légal de départ à la retraite" OR "retraite solidaire" OR "pénibilité " OR "salaire moyen" OR "cdi" OR "cdd" OR "délocalisations" OR "relocalisation" OR "revenu universel" OR "revenuuniversel" OR "contrat de travail" OR "syndicalisation" OR "cotisations sociales" OR "détachement des travailleurs" OR "travailleurs détachés" OR "délocalisation" OR "relocalisation" OR "plans sociaux"

- ***Economy & Taxation*** "révolution fiscale" OR "coût de la vie" OR "pouvoir d'achat" OR "libre échange" OR "libéralisme économique" OR "économie sociale et solidaire" OR entreprise* OR "pme" OR "pmi" OR actionnaire* OR relocalisation* OR "crédit impôt" OR nationalisation* OR commerçant* OR "plus-values" OR délocalisation* OR "scop" OR "niches fiscales" OR "gafa" OR "cice" OR "taux d'imposition" OR "retenue à la source" OR "crédit impôt-recherche" OR "impôt sur les sociétés" OR "services innovants" OR "marchés publics" OR "livret d'épargne industrie" OR "exportation" OR "impôt sur la fortune " OR "conseils d'administration" OR "small business act" OR "surplus" OR "capital-risque" OR "comités de rémunération" OR "représentants des salariés" OR "harmonisation fiscale" OR "complexité administrative" OR "reprise d'entreprises" OR "stock options " OR "start-ups" OR "pme européennes" OR "commandes publiques" OR "fiscalité européenne" OR "impôt minimal" OR "chiffre d'affaires mondial " OR "prélèvement à la source" OR "échanges commerciaux" OR " travailleurs handicapés" OR " OR "commerce indépendant" OR "politique commerciale" OR "développement économique" OR "politique commerciale" OR "charges patronales" OR "tva anti-délocalisations" OR "allègements de charges" OR "cice" OR "alléger les charges patronales" OR "allégement des charges patronales" OR "centrales d'achat" OR "concurrence internationale " OR "exploitations familiales" OR "financiarisation" OR "travailleurs indépendants" OR "liberté syndicale" OR "austérité" OR fisc* OR "impôt OR "imposition" OR "cotisations sociales" OR "retenue à la source" OR "tranche supplémentaire" OR "isf" OR "prélèvement à la source" OR "cotisations chômage" OR "exonérations de cotisation sociale" OR "tva" OR "charges patronales" OR "tva" OR "csg" OR "taxe" OR "allègements de charges" OR "cice" OR "trésor public" OR "droits de mutation" OR "dette des états" OR "dette de l'états" OR "dette publique" OR banque* OR financi* OR "subprimes" OR bancaire* OR "épargne" OR spécul* OR "livret A" OR "agios" OR "taux d'usure" OR emprunt* OR "agence de notation" OR "crédit à la consommation" OR "surendettement" OR "assurance-vie" OR "loi sapin" OR "banque centrale européenne" OR "euro-obligations " OR "pacte de responsabilité " OR "taxe mondiale sur les transactions financières" OR "taxe Tobim" OR "dépôts des particuliers" OR "crise bancaire" OR "crise financière" OR trader* OR "ras-le-bol fiscal"

- ***Democracy*** "front républicain" OR "ni-ni" OR "non-cumul des mandats" OR "conseil constitutionnel" OR "ve république" OR "6e république" OR "6eme république" OR "5e république" OR "vie politique" OR "données publiques" OR "%peneloppe%" OR "%pénélope%" OR "%pénéloppe%" OR "démocratie sociale" OR "cour des comptes" OR "proportionnelle" OR "parité " OR "assemblée nationale" OR "élections législatives" OR "fonction publique territoriale" OR "formation des fonctionnaires" OR "durée du quinquennat" OR "préfet" OR "établissements publics" OR "cour européenne des droits de l'Homme" OR préférence nationale" OR "nationalistes" OR "patriotes" OR "progressistes" OR %penelope%" OR parlement* OR élu* OR "corruption" OR "ena" OR "révisions constitutionnelles " OR "réserve parlementaire" OR "statut pénal du chef de l'état" OR scandale OR (marine AND 300000) OR (marine AND "300 000") OR ("Le Pen" AND 300000) OR ("le pen" AND "300 000") OR "inéligibilité" OR "fonction présidentielle" OR "e-démocratie" OR "49.3" OR "déclaration de patrimoine" OR "égalité républicaine"

- ***Research & Education*:** lycée* OR "pisa" OR "éducation" OR apprentissage* OR "maternelle" OR "décrochage scolaire" OR "scolarité" OR "scolaire" OR "service civique" OR "savoirs fondamentaux" OR "méthodes pédagogiques" OR "enseignement professionnel et technologique" OR "établissements scolaires" OR "tutorat" OR "échec scolaire" OR "écoles d'ingénieurs" OR "financer leurs études" OR " banque de la jeunesse" OR "écoles de commerce"OR "e-learning" OR "éducation artistique" OR "enseignement primaire" OR universit* OR "la recherche" OR chercheur* OR "R&D" science* OR scientifique* OR enseignant* OR professeur* OR "loi lru" OR "enseignants-chercheurs" OR "enseignement supérieur" OR "grandes écoles" OR "investissements d'avenir" OR "loi fioraso" OR "crédit impôt-recherche" OR "conseil national des universités" OR "CNU" OR "autonomie des universités" OR "universités" OR "ZEP"

- ***Immigration & Multiculturalism*:** migr* OR immigr* OR "nationalité" OR clandest* OR "droit de séjour" OR "schengen" OR "pays d'origine" OR "contrôle des frontières" OR "réadmission" OR "régularisations" OR "regroupement familial" OR naturalisation* OR "reconduites à la frontière" OR "titres de séjour" OR "procédures d'expulsion" OR "séjour des étrangers" OR "libre circulation des personnes" OR "connaissance préalable de la langue française" OR "intégration des étrangers" OR "citoyenneté française" OR "droit du sol" OR "droit du sang" OR islam* OR musul* or muzz* OR salaf* OR "burkini" OR "barbarie" OR "laïcité" OR juif* OR juive* OR chrétien* OR "communautarisme" OR

"multiculturalisme" OR "modèle républicain" OR "identité nationale" OR "civilisation française" OR "port du voile" OR "préférence raciale" OR race OR "demande d'asile" OR "submersion migratoire"

- ***Health***: "santé" OR médecin* OR médic* OR hôpit* OR "sécurité sociale" OR "autisme" OR handicap* OR "mdph" OR "désamiantage" OR "cancer" OR "assurance-maladie" OR "hébergement en établissement" OR "maintien à domicile" OR "soins d'urgence" OR "urgences sanitaires" OR "maladies" OR "perturbateurs endocriniens" OR "accès aux soins" OR "hospitalisation" OR "soins dispensés" OR "parcours de soins" OR " centre de soin" OR "malentendants" OR "malvoyants" OR "dépassements d'honoraires" OR "transports sanitaires" OR "arrêt de travail" OR "numerus clausus" OR "carte vitale" OR "perte d'autonomie" OR "ehpad" OR "agences de sécurité sanitaire et alimentaire" OR "tiers payant" OR "recours aux génériques" OR "lunettes"

- ***Ecology & Environment***: écolo* OR "biodiversité" OR "protection environnementale" OR "protection de l'environnement" OR pollution* OR "diesel" OR "ogm" OR "changement climatique" OR "fessenheim" OR "flamanville" OR "gaz de schiste" OR "pesticides" OR "gaz à effet de serre" OR "ressources naturelles" OR "quotas de pêche" OR "agroécologique" OR "énergies marines renouvelables" OR "pêche illégale" OR "performance énergétique" OR "rénovation thermique" OR "isolation de l'habitat" OR "précarité énergétique" OR "isolation de l'habitat" OR "forêts" OR "sylvicultures" OR "changements environnementaux" OR "services écosystémiques" OR "déforestation" OR "aires protégées" OR "émissions de carbone" OR "émission de CO2" OR "empreinte carbone" OR "gaz à effet de serre" OR "politique climatique" OR "durabilité" OR "obsolescence programmée" OR "niveau des mers" OR "niveau de la mer" OR "vagues de chaleur"

- ***Energy Policy***: "politique énergétique" OR électricité" OR "nucléaire" OR "énergies renouvelables" OR "éolien" OR "énergies marines renouvelables" OR "énergie renouvelable" OR "énergie solaire" OR "epr" OR "flamanville" OR "charbon" OR "centrales thermiques" OR "diesel" OR "fessenheim" OR "flamanville" OR éol* OR "performance énergétique" OR "fukushima" OR "rénovation thermique" OR "isolation de l'habitat" OR "précarité énergétique" OR "gaz de schiste" OR "énergies marines renouvelables" OR "isolation de l'habitat" OR "sources d'énergie" OR "transition énergétique" OR "conversion écologique" OR "énergies alternatives" OR "dépendance énergétique" OR "combustibles fossiles" OR "énergies fossibles" OR "efficacité énergétique"

- ***Foreign Policy***: europ* OR souverain* OR "CETA" OR "TAFTA" OR "bruxelles" OR "russie" OR "Poutine" OR "les russes" OR "pacte de stabilité" OR "directive européenne" OR "traité franco-allemand" OR "trump" OR "washington" OR "maison blanche" OR "moscou" OR "crise syrienne" OR "libye" OR "iran" OR "irak" OR "syrie" OR "OTAN" OR "traité transatlantique" OR "traités internationaux"

- ***Homeland security***: terror* OR "attentat" OR "cyber-sécurité" OR "guerre civile" OR "surveillance" OR délinqu* OR espion* OR "loi taubira" OR "vidéo-surveillance" OR "vidéo-protection" OR "sécurité des français" OR "laxisme judiciaire " OR "prescription" OR "peines planchers" OR "remises de peine" OR "Daech" OR "cyber-attaques" OR "cyber-attaque" OR "#cyberthreats" OR "#cyberthreat" OR "cyber-guerre" OR "cyber guerre" OR "cyber-menace" OR "#cybersecurity" OR "cyber-sécurité" OR "cyber sécurité" OR "cybersécurité" OR "cyberdéfense" OR "cyberarmée" OR "daech" OR "daesh"

- ***Agriculture***: agricult* OR agro* OR "pac" OR "alimentation" OR "engrais" OR "circuits courts" OR "transition agricole" OR "surplus" OR intrant* OR "bio" OR "pesticides"

G.2 Distribution of topics by political communities

We studied the diversity of communities discussing each theme. We calculated the entropy of the communities speaking on a theme (see Table D).

Although most of the themes have a fairly similar entropy, it appears that the theme *immigration and multiculturalism* and *homeland security* are discussed by a smaller number of communities because their entropy is low. Conversely, the theme *employment* is the one most uniformly discussed by all communities. In addition to entropy, we also show for each theme the two communities taking special account of this theme. As communities are very diverse in

size, we show the communities with the highest score of $tf\text{-}idf$ on a theme instead of the most present communities.

Thus, if a small community only speaks of one theme, it can still be part of the main communities on this theme even if in absolute value it participates less than others. The $tf\text{-}idf$ score of a c_j community for a t_i theme is calculated as follows:

$$tf\text{-}idf(c_j, t_i) = \frac{tweets(c_j, t_i)}{\sum_k tweets(c_k, t_i)} \; log\left(\frac{\sum_{k,l} tweets(c_k, t_l)}{\sum_l tweets(c_j, t_l)}\right) \; ,$$

where $tweets(c_j, t_i)$ is the number of tweets from the community c_j on theme t_i.

Theme	Entropy	# communities	Main communities
Agriculture	2.37	5.2	François Fillon, Marine Le Pen
Economy & Taxation	2.32	5.0	François Fillon, Jean-Luc Mélenchon
Employment	2.41	5.3	Jean-Luc Mélenchon, Benoît Hamon
Democracy	2.32	4.9	Emmanuel Macron, Marine Le Pen
Ecology & Environment	2.14	4.4	Benoît Hamon, Jean-Luc Mélenchon
Immigration & Multiculturalism	1.58	3.0	Marine Le Pen, François Fillon
Research & Education	2.41	5.3	François Fillon, Emmanuel Macron
Health	2.38	5.2	François Fillon, Benoît Hamon
Homeland security	2.07	4.2	Marine Le Pen, François Fillon
Energy Policy	2.19	4.6	Jean-Luc Mélenchon, Benoît Hamon
Foreign Policy	2.37	5.2	Marine Le Pen, Emmanuel Macron
All themes	2.31	5.0	Marine Le Pen, François Fillon

Table D. Entropy of 3−communities discussing each theme. The 2 main communities according to the $TF-IDF$ score are also displayed for each theme. The maximum entropy is 2.585 when the 6 communities have the same volume of tweets on this theme.

We also studied the diversity of themes discussed in each community by calculating the entropy of themes in each community (see Table 8 of the paper).

On this point, there are disparities between the candidates. However, the themes that we have defined are not exhaustive and do not allow us to conclude on the diversity of themes addressed by the candidates.

In order to better understand the representativeness of these themes and the presence of each candidate, the evolution of the proportion of tweets belonging to a theme and the evolution of the proportions of each theme are presented in Figs I to N.

G.3 Evolution of community vocabulary

The companion platform of this article, the *Politoscope* (https://presidentielle2017.politoscope.org/dashboard) proposes to monitor the use of the 15 keywords most specific to each of the main political communities when they deal with each of the eleven selected themes. These keywords were identified using the aggregation algorithm based on the χ^2 test implemented in Elasticsearch[4].

[4]See https://www.elastic.co/guide/en/elasticsearch/reference/current/search-aggregations-bucket-significantterms-aggregation.html et Manning et al., Chapter 13.5.2 - https://nlp.stanford.edu/IR-

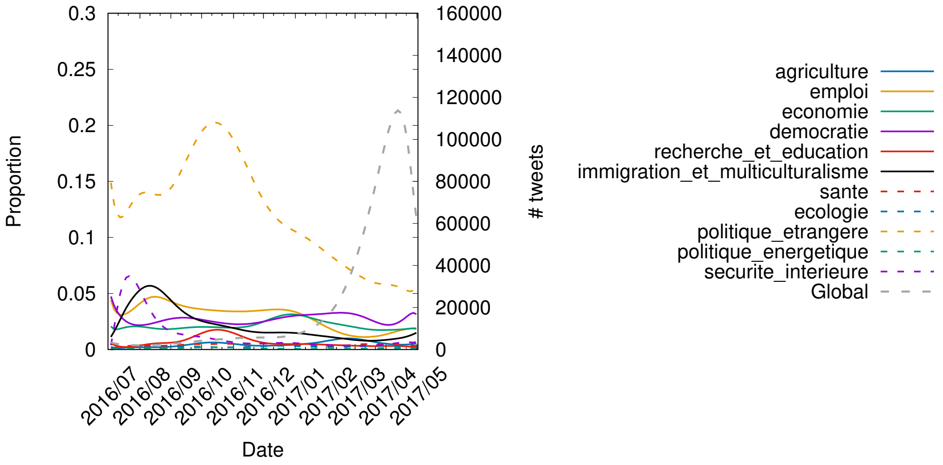

Fig I. Evolution of the topics discussed by the community François Asselineau between June 01, 2016 and May 08, 2017.

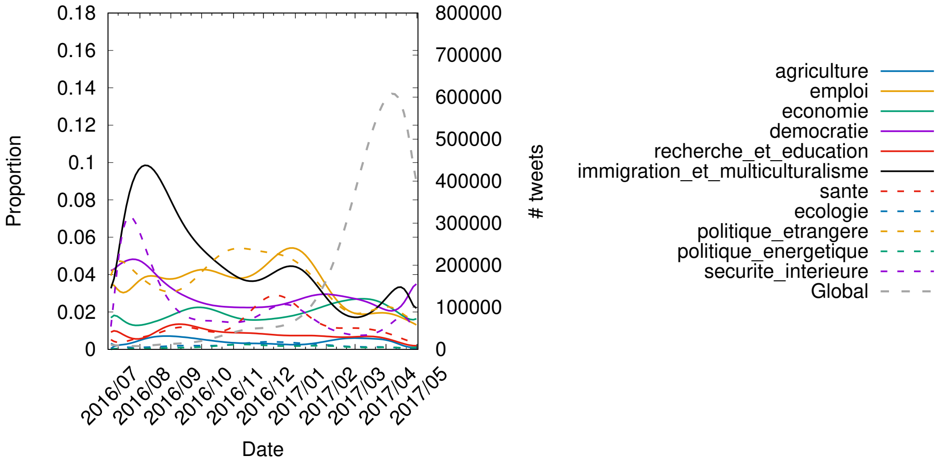

Fig J. Evolution of the topics discussed by the community François Fillon between June 01, 2016 and May 08, 2017.

It can be observed that, in addition to local semantic specificities at the level of the communities, the use of the vocabulary evolves over time for the different communities, with certain terms appearing, strengthening or disappearing along the way.

Here are a few examples, the reader can refer to the platform for an exhaustive exploration.

book/pdf/irbookonlinereading.pdf

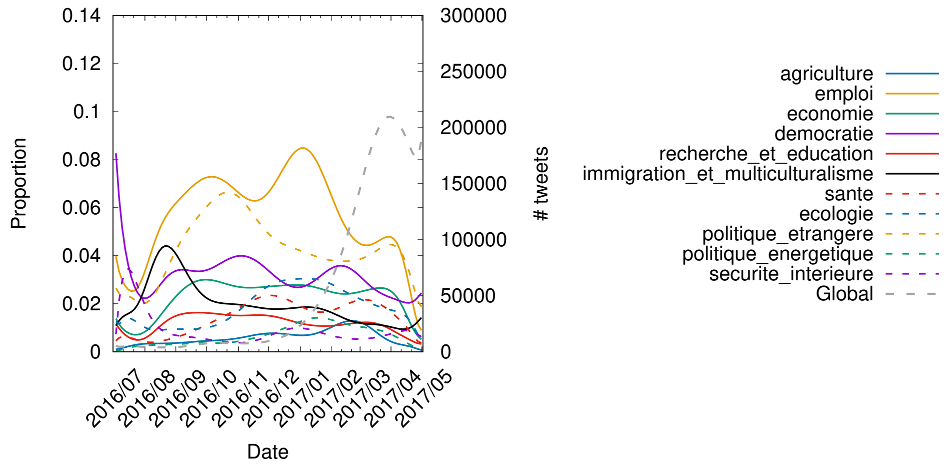

Fig K. Evolution of the topics discussed by the community Benoit Hamon between June 01, 2016 and May 08, 2017.

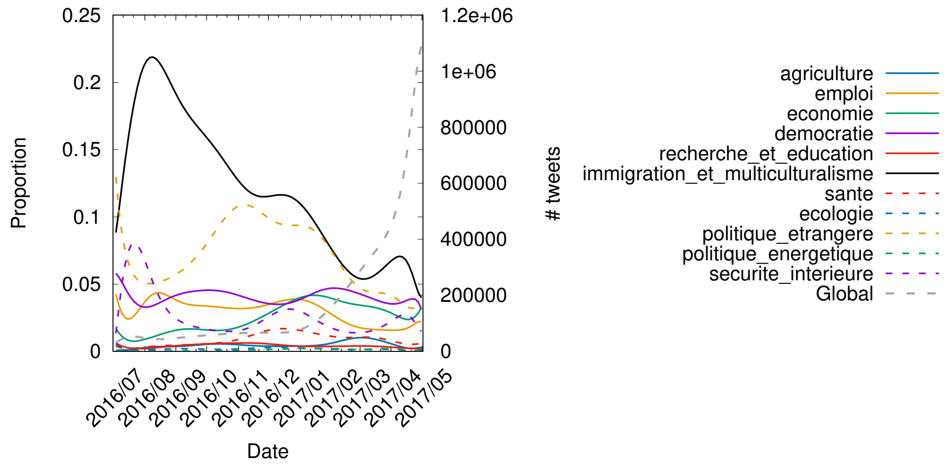

Fig L. Evolution of the topics discussed by the community Marine Le Pen between June 01, 2016 and May 08, 2017.

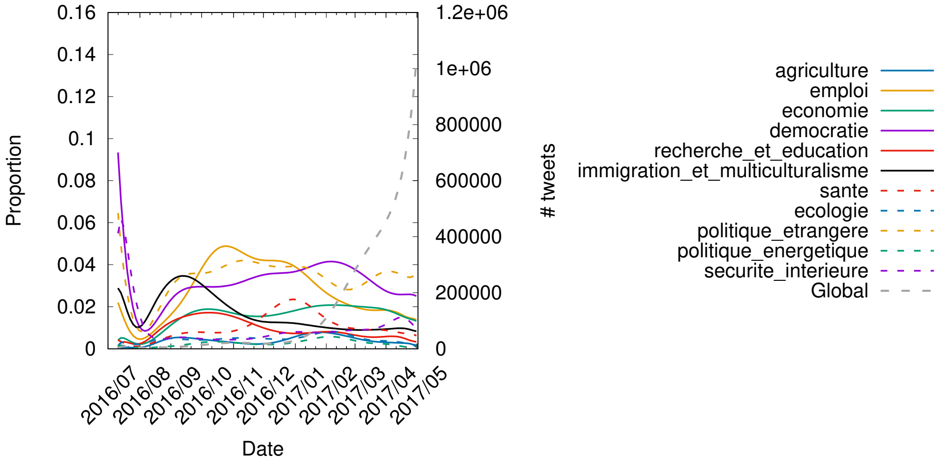

Fig M. Evolution of the topics discussed by the community Emmanuel Macron between June 01, 2016 and May 08, 2017.

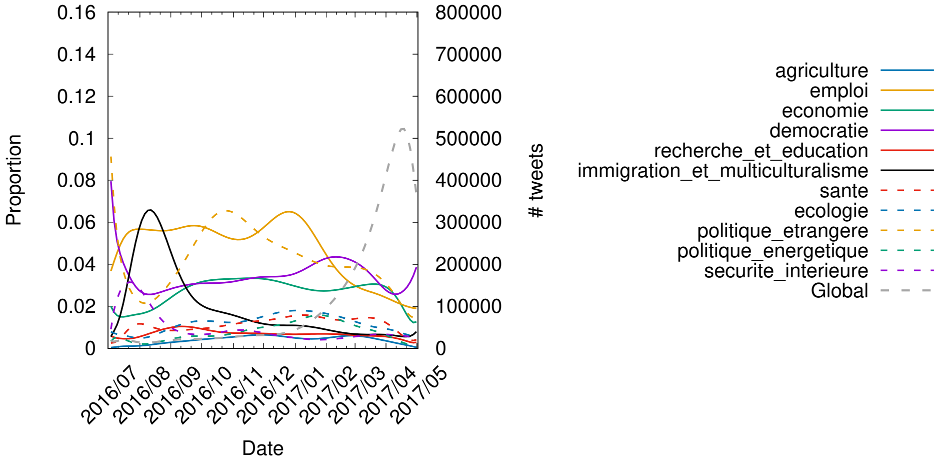

Fig N. Evolution of the topics discussed by the community Jean-Luc Mélenchon between June 01, 2016 and May 08, 2017.

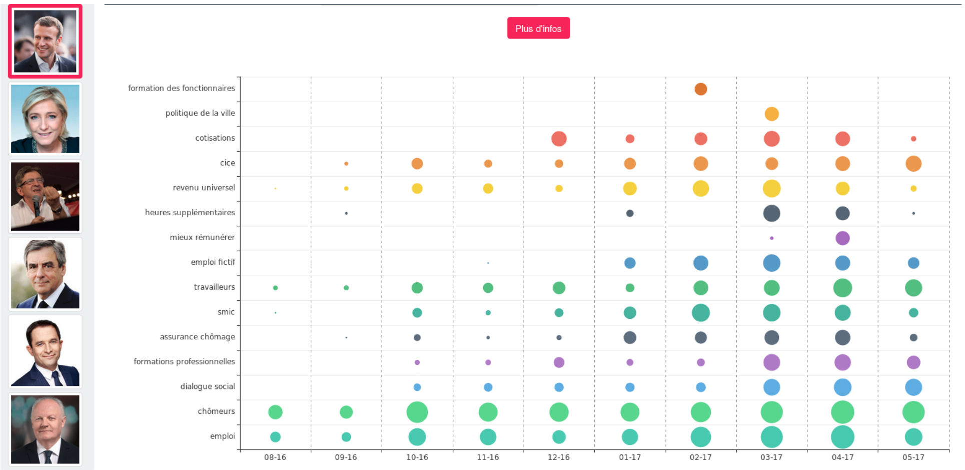

Fig O. Monthly usage of the 15 most specific terms in the Macron community for the topic *employment*.

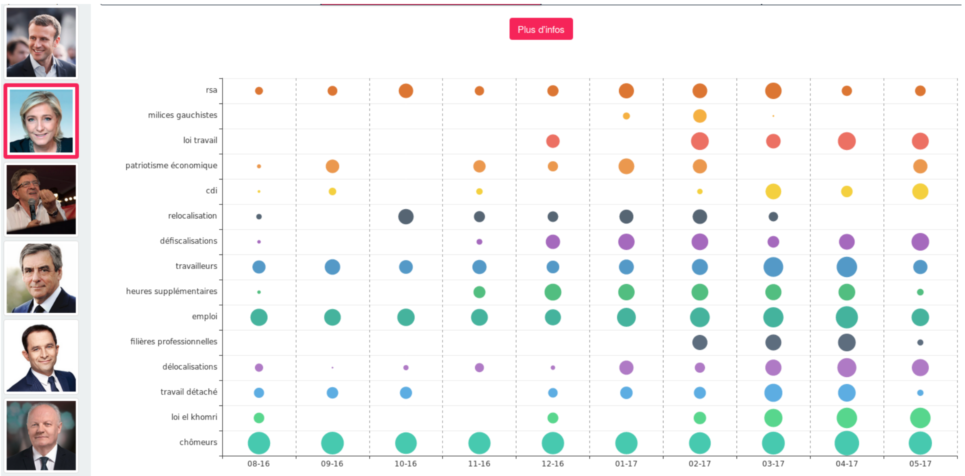

Fig P. Monthly usage of the 15 most specific terms in the Le Pen community for the topic *employment*.

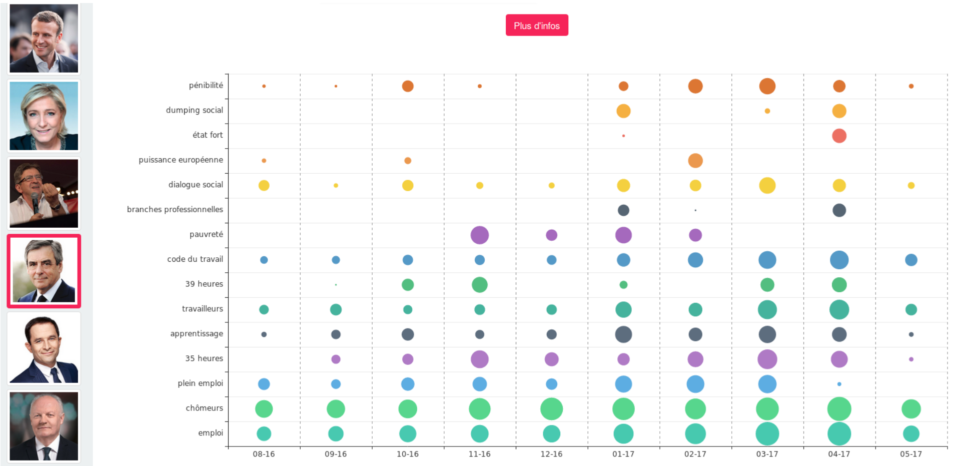

Fig Q. Monthly usage of the 15 most specific terms in the Fillon community for the topic *employment*.

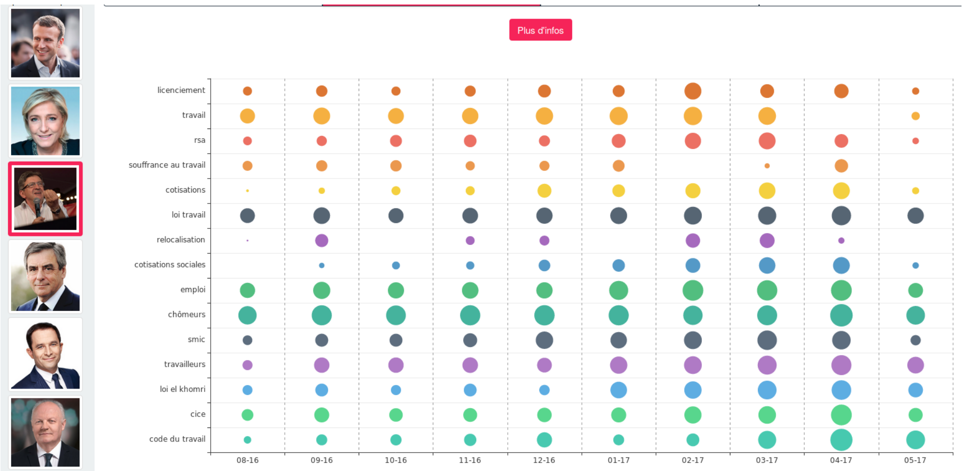

Fig R. Monthly usage of the 15 most specific terms in the Mélenchon community for the topic *employment*.

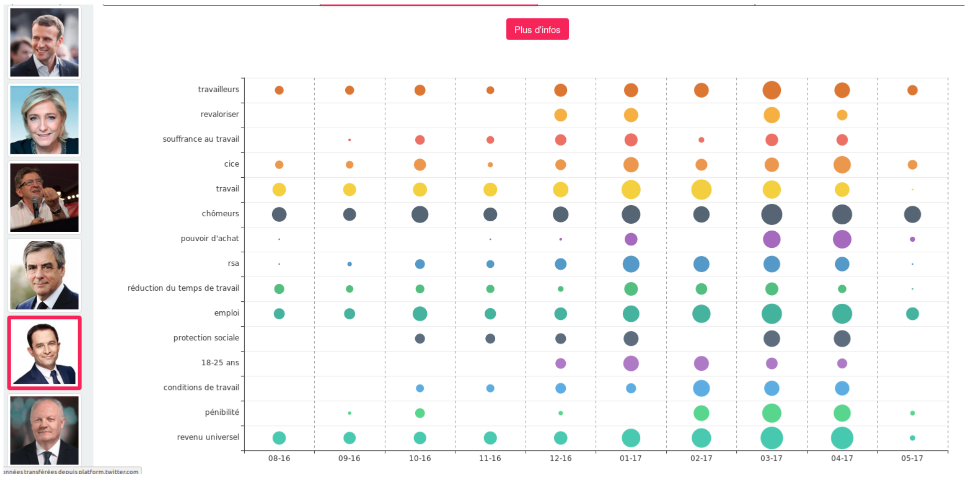

Fig S. Monthly usage of the 15 most specific terms in the Hamon community for the topic *employment*.

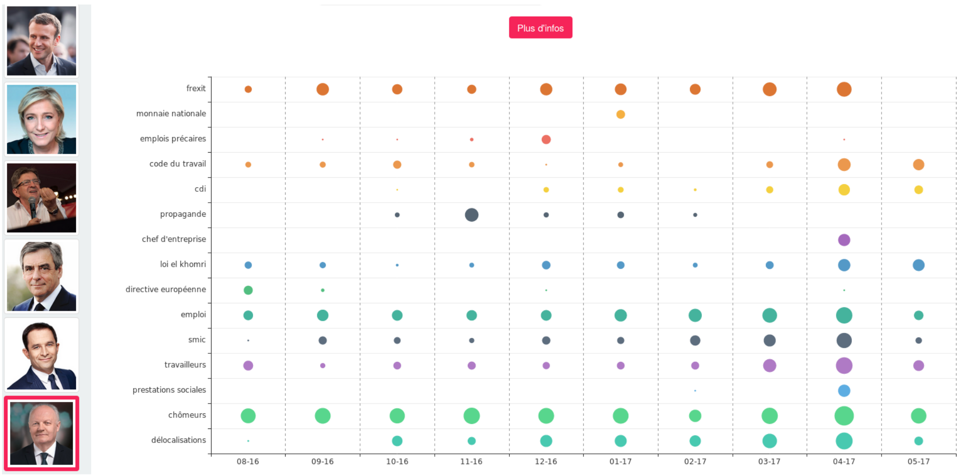

Fig T. Monthly usage of the 15 most specific terms in the Asselineau community for the topic *employment*.

H Fake news data cleaning

In order to track fake news and debunks in the *Politoscope* database, we normalized all the URLs contained in tweets. To avoid false negatives caused by the use of url shortener service such as bit.ly or fb.me, we replaced URLs by the URLs reached when querying them. We also made sure to ignore the query options added to track people and we did not take into account the use of *http* or *https* as it refers to the same website.

We screened the list of links that were both present in our dataset and in the 5,148 fake news links provided by the *Decodex*. We removed 4 urls that we did not consider as containing fake news because of their openly parodic presentation by the website or unclear content at the moment of our screening (in brackets, the number of tweets mentioning the link in $\mathcal{D}$):

- [158] https://www.buzzbeed.com/macron-veut-faire-payer-un-loyer-aux-proprietaires,
- [3,642] http://www.legorafi.fr/2017/04/23/la-belgique-annonce-la-construction-dun-mur-pour-repousser-les-vagues-dimmigrants-francais,
- [98] http://www.legorafi.fr/2016/06/01/emmanuel-macron-quand-je-serre-la-main-dun-pauvre-je-me-sens-sale-pour-toute-la-journee,
- [54] http://candidat-2017.fr

This cleaning has not decreased the number of stories.

We checked that the mentions in tweets of URLs related to fake news and debunk reflect the spreading of both the associated fake news and debunks within the online political communities. To this aim, we selected a random sample of 100 tweets with fake news links mentions and a sample of 100 tweets with debunk links mentions in order to manually evaluate them. 100% of tweets with a fake link were spreading fake news - $95\% CI = [96.3\% \ ; \ 100\%]$ [5]) and 98% of tweets with a debunk link mention were spreading the debunk - $CI = [93.0\% \ ; \ 99.8\%]$).

[5] The confidence interval are Clopper–Pearson interval.

I Vocabulary of the political discourse of the presidential election 2017

The terms considered equivalent have been grouped together and appear in the following analyses under a label which is the first term of the group. A csv version of this terms list is provided by File A9 at DOI:10.7910/DVN/AOGUIA.

List of term groups extracted with the help of the Gargantext software (https://gargantext.org).

- #frexit OR frexit
- 18-25 ans
- 300 000 euros OR 300 000 €OR 300 000€
- 35 heures OR 35h
- 37 heures
- 39 heures
- 6ème république OR 6emerepublique OR 6erepublique OR 6erépublique
- abattements fiscaux
- abattoirs
- abroger OR abrogation
- abus de position dominante
- accès à la propriété
- accès aux soins
- accessibilité des personnes handicapées
- accord de libre-échange
- accords bilatéraux
- accords de schengen
- accords du touquet
- accueil des réfugiés
- accusé
- actifs
- action économique
- action publique
- actionnaires
- actionnariat salarié
- actions d'entreprises
- activités de conseil
- administration centrale
- administration fiscale
- administration pénitentiaire
- administratives OR administration OR administrations
- aéroports
- affaires criminelles
- afghanistan
- afp
- afrique
- âge légal de départ à la retraite
- agence publique européenne de notation
- agences de sécurité sanitaire et alimentaire
- agents publics
- agglomération
- agios
- agriculture OR agriculteurs OR agricole OR agricoles OR agriculteur
- agriculture biologique OR agriculture bio
- agroécologique
- aide à la construction
- aide médicale d'état OR aide médicale d'etat
- aide sociale à l'enfance
- aides publiques
- aides sociales OR aide sociale
- airfrance
- algérie
- alimentation
- allègement
- allègements de charges
- allégements fiscaux
- alléger les charges patronales
- allemagne
- allocation adulte handicapé OR aah
- allocation aux adultes handicapés
- allocations OR allocs
- allocations chômage
- allocations familiales OR allocation familiale
- allocations sociales
- alpes-maritimes
- alstom
- alternance
- alternance forte
- aluminium
- alzheimer
- aménagement du territoire
- amendes
- anciens combattants
- animaux
- ankara
- apa
- apl
- apologie du terrorisme
- apologie du terrorisme
- apple
- apprentis
- apprentissage OR apprentissages
- l'arabie saoudite OR l'arabie saoudit
- argent public
- armée d'afrique
- armées OR armée
- arrangements fiscaux
- arrêt de travail
- artisanat local
- artisans OR artisanat
- artistes OR artiste
- aspa
- assange

- assassinat politique
- assassiner OR assassins
- assemblée constituante
- assemblée nationale
- assimilation
- assistance médicalisée
- assistant parlementaire OR assistante parlementaire
- associations OR association
- assurance-chômage
- assurance-maladie OR assurance maladie
- assurance-vie
- atteindre l'équilibre budgétaire
- attentatnice
- attentats OR attentat
- augmenter
- augmenter le salaire
- austérité
- autisme
- autonomie aux communes
- autonomie fiscale
- autorité
- autorité de l'état
- autorité hiérarchique
- autorité indépendante
- autorités françaises
- autoroutes
- auxiliaire de vie scolaire
- avions ravitailleurs
- avocats
- avortement
- avs
- ayant-droits
- baccalauréat
- bachar al-assad
- baisse d'impôts OR baisses d'impôts OR baisse d'impôt
- baisse des dépenses interventions de l'etat
- baisser
- baisser les prix
- balkany
- banlieues OR banlieue
- banque centrale
- banque centrale européenne OR bce
- banque de détail
- banque de données
- banque de la jeunesse
- banque postale
- banque publique d'investissement
- banques OR banque
- banques d'affaires
- banques de dépôt OR banques de dépôts
- barbarie OR barbare OR barbares
- barbarie terroriste
- bas revenus
- bâtiments
- bâtiments publics OR bâtiments nationaux
- bayrou
- bénéficiaires du rsa
- bénévoles
- benoît hamon
- bi-langues
- bibliothèques
- bien-être animal
- bien-être des animaux
- biens communs
- bio
- biodiversité
- biogaz
- blessés
- blindés
- blocages
- bois
- bonus
- bonus-malus
- bonus/malus
- branches professionnelles
- brexit OR #brexit
- bricolage
- bruxelles
- budget européen
- budgets participatifs
- bureaucratie
- burkini OR #burkini
- burnout
- burqa
- buy european act
- bygmalion
- cadeaux fiscaux
- caf
- caisse des dépôts et consignations
- caissière
- calais
- cancers OR cancer
- cannabis
- cantine
- cantines bio
- cantines scolaires
- capital-risque
- caricature OR caricatures
- carnées
- carrières judiciaires
- carte de séjour
- carte vitale biométrique
- casier judiciaire
- caution solidaire
- caution universelle
- cavous
- cdd
- cdi OR contrat à durée indéterminée
- censure
- centrales OR central OR centrale
- centrales d'achat
- centrales nucléaires
- centres éducatifs fermés
- ceseda
- ceta
- chaînes publiques de télévision
- chambres régionales des comptes
- champ d'honneur
- changement climatique
- charges OR charge

- charges patronales
- charges salariales
- charges sociales salariales
- chef d'entreprise OR chefs d'entreprise OR chefs d'entreprises
- chef de l'état
- chefs d'etat OR chef d'etat OR chef d'état OR chefs d'état
- chiffre d'affaires mondial
- chine
- chirurgie ambulatoire
- chômeur de longue durée
- chômeurs OR chômeur OR chômage
- chrétiens d'orient OR chretiensdorient
- cice
- circuits courts
- citoyenneté française
- citoyens
- civilisation européenne
- civilisation française
- civilisations OR civilisation
- clandestine
- clandestins OR clandestin
- classe politique
- classes moyennes
- clause générale de compétence
- clients des banques
- climat
- clubs professionnels
- cnspf
- cocaïne
- code civil
- code de l'entrée et du séjour des étrangers
- code de la nationalité française
- code du travail
- cohésion nationale
- cohésion sociale
- collectivités OR collectivité
- collectivités d'outre-mer
- collectivités locales
- collectivités territoriales OR collectivité territorial
- collèges OR collège
- combattants
- comités de rémunération
- commandes publiques OR commande publique
- commerçants
- commerçants indépendants
- commerce
- commerce indépendant
- commission européenne
- communautarisme OR communautarismes
- communautés urbaines
- communiste OR communistes
- compassion
- compensation salariale
- compétitivité
- complexité administrative
- complot OR complotiste
- compte bancaire
- compte individuel de formation
- comptes publics
- concertation
- concurrence OR concurrents
- concurrence déloyale
- concurrence internationale
- concurrence loyale
- concurrence sociale déloyale
- condamnations OR condamnation OR condamnés
- conditions de travail
- confession juive OR juif OR juifs
- confiance confiance
- conflits d'intérêt
- congé parental
- conjoints survivants
- connaissance préalable de la langue française
- conseil constitutionnel
- conseil de sécurité
- conseil national des universités
- conseil supérieur de l'audiovisuel
- conseil supérieur de la magistrature OR csm
- conseiller territorial
- conseils d'administration
- conseils généraux
- conseils régionaux
- consommations OR consommer OR consommation OR consommateurs
- consommation responsable
- constitution
- construction
- construction européenne
- contrat de génération
- contrat de professionnalisation
- contrats d'assurance
- contribution fiscale
- contrôle de légalité a priori
- contrôle des frontières OR contrôle de ses frontières
- contrôles d'identité
- convention de dublin
- convention européenne des droits de l'homme
- conventions citoyennes
- conversion écologique
- conversion écologique et énergétique
- convocation
- convocation judiciaire OR convocation des juges
- cop21
- corporatisme
- correctionnalisation
- corruption
- cotisation vieillesse
- cotisations OR cotisation OR cotiser
- cotisations chômage
- cotisations sociales

- coup d'État institutionnel
- coupables
- couples homosexuels
- cour des comptes
- cours d'assises
- coût de la vie
- coût du travail
- couverture mobile
- création artistique
- création d'entreprise
- crèches
- crédit à la consommation
- crédit impôt OR crédit d'impôt
- crédit impôt réparation
- crédit impôt-recherche OR crédit d'impôt recherche OR crédit impôt recherche
- crédits OR crédit
- crimes OR crime
- criminel OR criminels
- crise
- crise bancaire
- crise bancaire économique
- croissance
- csg
- culture OR cultures
- culture française
- cumul
- cyber-attaques OR cyber-attaque OR #cyberthreats OR #cyberthreat OR cyber-guerre OR cyber-guerre OR cyber guerre OR cyber-menace
- #cybersecurity OR cyber-sécurité OR cyber sécurité OR cybersécurité
- cyberdéfense OR cyberarmée
- daech OR daesh
- décentralisation
- déchets
- décisions de remise en liberté
- déclassement
- découverts bancaires
- décrochage scolaire OR déscolarisé
- décrocheurs
- déductibilité fiscale des dons
- défense de la laïcité
- défense européenne commune
- défense nationale
- déficit
- défiscalisations OR défiscaliser OR défiscalisation OR défiscalisées
- dégradation du mobilier urbain
- délais de jugement
- délinquants étrangers
- délit OR délits OR délinquance OR délinquante OR délinquants
- délocalisations OR délocalisent OR delocalisees OR délocalisé
- demandes d'asile OR demandeurs d'asile
- dématérialisation OR dématérialisée
- démocratie OR démocraties OR democracy OR democracies
- démocratie sociale
- dentaires
- départements OR département
- dépassements d'honoraires
- dépendance
- dépendance au pétrole
- dépenses administratives
- dépenses culturelles
- dépenses d'investissement
- dépenses de l'etat OR dépenses publiques OR dépenses de fonctionnement et d'intervention de l'etat OR dépense publique
- dépôts des particuliers
- dérives jurisprudentielles
- désamiantage
- désendettement
- désengorger les urgences
- déserts médicaux
- déserts universitaires
- détachement des travailleurs
- détourné d'argent public OR détournement d'argent public
- dette OR dettes
- dette des etats OR dette des états OR dette publique
- développement durable
- développement économique
- devenir entrepreneurs
- dialogue social
- dictatures OR dictature
- diesel
- dieu
- diffusion de l'art
- dignité
- diplomatie
- directive européenne
- directives de libéralisation
- discrimination
- discrimination positive
- discriminations
- dislocation
- dispositif de renseignement
- dissuasion nucléaire
- distribution d'électricité
- diversité culturelle
- dividendes
- djihadistes OR djihad
- dmto
- doctorants
- documents officiels
- donald trump OR d.trump OR donaldtrump OR d..trump OR d trump
- donations
- données publiques
- dons
- dotations de l'état OR dotations de l'etat
- double nationalité
- drapeau français
- drogue OR drogues

- droit à l'oubli
- droit d'auteur OR droits des créateurs
- droit d'être syndiqué
- droit de faire appel des décisions
- droit de mourir dans la dignité
- droit de séjour
- droit de veto
- droit de vote
- droit de vote des étrangers
- droit du sol
- droit européen
- droit français
- droits à construire
- droits civiques
- droits culturels
- droits de douane
- droits de mutation OR droit de mutation
- droits de succession
- droits humains
- drones
- dumping social
- durée du quinquennat
- e-learning
- eau OR eaux
- eaux
- échanges commerciaux
- échec scolaire
- éco-conception
- école OR écoles
- école nationale de la magistrature OR ecole nationale de la magistrature OR ENM
- écoles d'ingénieurs
- écoles de commerce
- écoles primaires OR école primaire
- écologie OR écologique OR écologiques OR ecologie
- écologistes
- économie OR économies OR economie
- économie maritime OR economiedelamer
- économie numérique
- économie réelle
- économie sociale et solidaire
- économies sur les dépenses
- économiste OR économistes
- edf
- éducatives OR éducation
- éducation artistique
- éducation nationale
- eelv
- effectifs de l'etat OR effectifs de l'état
- effort de défense
- effort national
- égalité OR egalite
- égalité républicaine
- égalité salariale
- ehpad
- el khomri
- électeurs
- élection présidentielle OR élections présidentielles
- élections OR élection
- élections législatives
- élections locales
- électricité
- élevage
- élevage intensif
- élèves
- éleveur OR éleveurs
- élu
- élucidation des affaires
- élus condamnés
- élus locaux
- embaucher
- émissions de gaz à effet de serre
- emotion
- empêcher les abus
- emploi OR emplois OR embauches OR embauche
- emploi fictif
- emploi présumé fictif
- emploi stable
- emplois précaires
- employés
- employeurs
- emprunts
- ena OR école nationale d'administration
- endettement de l'etat
- énergie OR énergies
- énergies marines renouvelables
- énergies renouvelables OR énergie renouvelable OR éolien OR énergie solaire
- éolien
- enfants OR enfant
- enfants récidivistes
- engrais
- enmarche
- enquêtes OR enquête
- enr
- enseignants OR enseignant
- enseignants-chercheurs
- enseignement élémentaire
- enseignement primaire
- enseignement professionnel et technologique
- enseignement supérieur
- enseignements OR enseignement OR enseigner
- ententes
- entrepreneuriat culturel
- entrepreneurs
- entreprises OR entreprise
- entreprises innovantes
- entreprises publiques
- épargne
- épargne bancaire
- épargne populaire
- epr
- équilibre budgétaire
- erasmus
- espaces maritimes
- espaces naturels protégés
- établissements hospitaliers
- établissements privés

- établissements publics
- établissements scolaires
- établissements universitaires
- etat de droit
- état fort
- état libre
- état palestinien
- état-major
- éthique OR éthiques
- étudiants
- étudiants étrangers
- euro-atlantistes
- euro-obligations
- europe OR union européenne OR ue
- exception culturelle
- exode des rapatriés
- exonérations OR exonération
- exonérations de cotisation sociale
- exonérer de charges patronales
- expérimentations animales
- expertise citoyenne
- exploitations familiales
- exportation
- exportations agricoles
- expulsions
- faceauxchrétiens
- facebook
- faciès
- falsification
- familles
- familles françaises
- fan-zones OR fan zone
- favoriser
- femme-homme OR femmes-hommes
- féodalités locales
- fermes-usines
- fessenheim
- fichés s
- fierté d'être français
- filières du travail clandestin
- filières industrielles
- filières professionnalisantes
- filières professionnelles
- fin de vie
- finance spéculative
- financement de la recherche
- financement étranger
- financement public
- financer leurs études
- finances publiques
- finances publiques locales
- financiarisation
- financiers
- fiscalité OR fiscal OR fiscales OR fiscale
- fiscalité européenne
- fiscalité locale
- fixation des tarifs
- flamanville
- florange
- flotte
- flux migratoires
- fnsea
- fonction présidentielle
- fonction publique OR fonctions publiques
- fonction publique hospitalière
- fonction publique territoriale
- fonctionnaires OR personnel de l'etat
- fonds européen de défense
- fonds régionaux
- forces armées
- forêts
- formation OR formations
- formation de nos jeunes
- formation des fonctionnaires
- formation initiale
- formations politiques
- formations professionnelles OR formation professionnelle
- fossiles
- frais bancaires
- françafrique
- france du désastre
- france métropolitaine
- franchises OR franchise
- françois asselineau
- françois hollande
- francophonie
- frapor
- fraternité
- fraude OR frauduleux OR fraudes
- fraude aux prestations sociales
- fraude fiscale
- fraude sociale
- fraudes commerciales
- frenchtech
- frères musulmans
- frontière OR frontières
- frontières extérieures
- frustration
- fuite des cerveaux
- g20
- gafa
- gains de productivité
- garde à vue
- garde d'enfants
- garde nationale
- garde-frontières
- gauche irréconciliable OR gauches irréconciliables
- gaulois OR gauloi
- gaz
- gaz à effet de serre
- gaz de schiste
- gel des traitements
- gel des transferts
- gendarmes OR gendarmerie
- général de gaulle
- génération
- gestion planifiée
- google
- gouvernement algérien
- gpa
- grand emprunt
- grand patronat

- grand remplacement
- grande bretagne OR grande-bretagne
- grande distribution OR grandes surfaces OR grands distributeurs
- grande guerre
- grande réforme fiscale
- grandes écoles
- grandes entreprises OR grands groupes OR grosses entreprises
- grèce
- gros patrimoines
- gros travaux
- guadeloupe
- guerre civile
- guerre mondiale
- guerres
- guyane
- gwada
- hacker
- hadopi
- harcèlement
- harki OR harkis
- harmonisation fiscale
- hébergement d'urgence
- hébergement en établissement
- héritage
- heures supplémentaires
- histoire nationale
- hlm
- hommage aux victimes
- hôpital OR hôpitaux hopital OR hopitaux
- hôpital public
- hôpitaux de proximité
- hospitalisation
- identité
- identité de genre
- identité nationale
- imams
- immigration de masse
- immigration illégale OR migrants clandestins OR immigration clandestine OR immigrés clandestins
- immigration légale
- immobilier
- importation OR importations
- impôt OR impôts OR imposition
- impôt minimal
- impôt sur la fortune
- impôt sur le revenu
- impôt sur les sociétés
- impôts locaux
- incivilités
- incubateurs
- indemnisation
- indemnités des élus locaux
- indemnités journalières
- indépendance de la justice
- indépendance stratégique
- industrie OR industriels
- industries stratégiques
- inéligibilité
- infirmières
- inflation
- ingénieurs OR ingénieur
- ingérence
- injustices OR injustice
- innovation OR innovations
- insertion des jeunes
- insertion professionnelle
- inspection générale de l'etat
- inspections générales interministérielles
- insultes
- intégration des étrangers
- intercommunalités OR intercommunalité
- interdiction OR interdire
- intérêt général
- intérêts économique OR intérêts économiques
- intérêts nationaux OR intérêt national
- intermittent du spectacle
- internet
- intrants
- investissement privé
- investissement public
- investissements d'avenir
- investissements étrangers
- iran
- irlande
- isf
- islam OR l'islam
- islamistes OR islamiste OR islamique OR islamiques
- isolation de l'habitat
- israël
- istanbul
- ivg
- jaures OR jaurès
- jdd
- jean-luc mélenchon
- jeune en alternance
- jeunesse OR jeunesses
- journalistes
- juges OR juge
- jungle OR jungles
- juppé
- jury de cour d'assise
- jurys citoyens
- justice
- justice des mineurs
- justice fiscale
- justice sociale
- kinésithérapeutes
- égalité femmes-hommes OR égalité femmes/hommes
- etat islamique OR état islamique OR ei
- les étrangers
- excellence environnementale
- extrême-droite OR extrême droite
- identité heureuse
- immigration
- islam politique
- facho OR fachos OR fachisme OR fachistes OR fachiste

- l'union européenne
- la poste
- la recherche OR chercheurs
- la république
- la russie
- labels de qualité
- lagarde
- laïcité
- langue française
- langues régionales
- latin
- laxisme
- laxisme judiciaire
- le pen
- légitime défense
- les collectivités
- les étrangers
- les jeunes OR jeunes OR la jeunesse
- libéralisation des services
- libéralisme économique
- libération conditionnelle
- liberonsnosconvictions
- liberté d'expression
- liberté religieuse
- liberté syndicale
- librairies indépendantes
- libre circulation
- libre circulation des personnes
- libre-échange
- libye
- licence globale
- licences professionnelles
- licenciement OR licenciements
- licenciements collectifs OR licenciement collectif
- lieux de culte
- litiges
- livret a
- livret d'épargne industrie
- locataires
- logements OR logement
- logements sociaux OR logement social
- loi d'orientation
- loi de 1905
- loi de moralisation de la vie publique
- loi el khomri
- loi fioraso
- loi hadopi
- loi lru
- loi religieuse
- loi sapin
- loi sru
- loi sur la protection des sources
- loi sur le renforcement de la démocratie et des libertés locales
- loitaubira OR loi taubira
- loitravail OR loi travail
- loyers
- lunettes
- lutte antiterroriste
- lutte contre l'évasion fiscale
- lutte contre l'immigration clandestine
- lutte contre l'optimisation fiscale
- lutte contre la contrefaçon
- lutte contre la discrimination
- lutte contre la fraude sociale
- lutte contre la précarité OR lutterai contre la précarité
- lutte contre les discriminations
- lutte contre les fraudes
- lycéens
- lycées OR lycée
- lycées professionnels
- machines OR machine
- madeinfrance
- magistrats OR magistrat OR magistrature
- maintien à domicile
- maisons de santé
- maisons de soins
- maitrise de la langue française
- maitrise de la masse salariale
- majorité silencieuse
- malades OR malade
- maladies OR maladie
- maladies graves ou contagieuses
- malentendants
- malvoyants
- mandat
- manifpolice
- marché commun
- marché unique européen
- marchés publics
- marges des producteurs
- mariage
- marine nationale
- maritime
- marseillaise
- massacre
- maternelle
- mdph
- mécénat populaire
- médecine
- médecins
- médecins généralistes
- medef
- médias
- médicaments
- méditerranée
- membres du parquet
- menace OR menaces
- menace terroriste OR menace terroristes
- ménages
- mensonges OR mensonge
- méritocratie
- mesures emblématiques
- mesures fiscales
- méthodes pédagogiques
- métiers de l'entreprenariat
- métiers de la finance
- métiers du droit
- microentreprise
- mieux rémunérer

- migrants
- migratoires
- militaires
- millefeuille administratif
- mineurs
- minimum vieillesse
- ministère de l'intérieur, de l'immigration et de la laïcité
- ministère des souverainetés
- ministres du culte
- mis en examen
- mixité sociale
- mlp_officiel
- mobilisation OR mobilise
- mobilité professionnelle
- modèle républicain
- modem
- modernisation
- moins de 18 ans
- monde carcéral
- monde judiciaire
- monde rural
- mondialisation
- monnaie
- monnaie nationale
- montauban
- montée de la délinquance
- moraliser
- moratoire
- mosquées OR mosquée
- moyen-orient OR moyen orient
- moyenne européenne
- moyens de nos armées
- multiculturalisme
- multilatéralisme
- multinationales
- musique OR musiques
- musulman OR musulmans OR musulmanes
- mutation digitale
- mutuelles
- nation OR nations
- nationalisation OR nationaliser
- nationalité
- nationalité française
- naturalisations OR naturalisation
- négociation salariale
- neutralité
- niceattentat OR attentat de nice
- niches fiscales OR niche fiscale
- nicolassarkozy
- non remplacement de un fonctionnaire sur deux OR non-remplacement d'un fonctionnaire sur deux
- non-cumul des mandats
- norme nationale
- normes OR norme
- normes environnementales
- normes européennes
- nos soldats
- notation sociale
- notre sécurité
- notredémocratie
- nouvelle calédonie OR nouvelle-calédonie
- nouvelles technologies
- nucléaire OR industrie nucléaire OR filière nucléaire OR part du nucléaire
- nuitdebout
- nullité de procédure
- numérique OR numériques OR numérisation
- numerus clausus
- obligation pour eux d'accepter un emploi
- obligations légales
- officines
- offre de logements
- offres en ligne
- ogm
- omc
- onema
- ong
- onu OR nations unies
- opa
- open data OR open-data
- opérateurs téléphoniques
- opérations extérieures OR opex
- opérations spéculatives
- opinion publique
- optimisation fiscale
- ordonnances
- organisations syndicales
- orthophonistes
- otan OR l'otan
- outil de défense
- outre-mer OR outre-mers
- ouvriers
- pac OR politique agricole commune
- pacs
- pacte de confiance
- pacte de responsabilité
- pacte de stabilité
- paix
- palestine
- paradis fiscaux
- parcours de soins
- parcours européens
- parcours professionnels
- parcours scolaire
- parents
- parisiens OR parisien
- parité
- parlement
- parlement européen
- parlementaire
- partenaires européens
- partenaires sociaux
- partenariats public-privé
- patrimoine
- patriotisme OR patriotes
- patriotisme économique
- patrons OR patron
- pays d'afrique
- pays d'origine
- pays de l'union OR état membre

- paysans OR paysanne OR paysannes
- pêche
- pêche illégale
- peines OR peine
- bi-nationalité OR binationalité OR binationaux
- déchéance de nationalité
- peines de prison OR peines d'emprisonnement
- peines de réclusion criminelle
- peines planchers
- penelope fillon OR fillongate
- pénibilité
- pensions OR pension
- performance énergétique
- période de sûreté
- periscope
- perpétuité
- personnels soignant OR personnel soignant OR personnels soignants
- personnes âgées
- personnes de plus de 55 ans
- personnes handicapées OR handicap OR handicaps OR handicapés OR personnes handicapés
- perte d'autonomie
- perturbateurs endocriniens
- pesticides
- petite enfance
- petites retraite OR petites retraites
- pétrodollars
- peuple
- peurs OR peur
- pib
- pisa
- places de prison
- places en crèche
- plan carcéral
- plan d'investissement
- plan de rénovation
- planète
- planification
- planification écologique
- planification stratégique
- plein emploi OR plein-emploi
- plus de 45 ans
- plus-value des terrains à bâtir
- plus-values OR plus-value
- pma
- pme
- pme européennes
- pmi
- pnru
- pôle emploi
- pôles de santé de proximité
- police OR policiers
- politique agricole commune
- politique commerciale OR politiques commerciales
- politique d'application des peines
- politique de coopération
- politique de défense
- politique de la ville
- politique étrangère
- politique industrielle de défense
- politique nataliste
- politiques culturelles
- politiques publiques
- pollution atmosphérique
- pollutions OR pollution OR polluants OR polluant
- polynésie
- pompiers
- porte-avions
- position dominante
- postes supplémentaires
- poursuites disciplinaires
- poutine OR putin
- pouvoir d'achat
- pré-apprentissage
- précarité
- précarité énergétique
- préfet
- prélèvement à la source
- prélèvements indexés sur le revenu
- prélèvements obligatoires
- premier ministre
- prescription à 10 ans
- prescriptions OR prescription
- présomption d'innocence
- prestations sociales
- prêts aidés
- prévention
- primaire à droite
- primaires citoyennes OR primairescitoyennes
- prime à la détention longue de terrains constructibles
- prime annuelle
- prime pour l'emploi
- primes OR prime
- principe de précaution
- priorité d'accès
- priorité nationale
- prison OR prisons
- prison ferme
- prison supplémentaires
- privatisation OR privatiser OR privatisations
- privilège
- prix des médicaments
- prix planchers
- procédures d'expulsion
- procès
- processus judiciaire
- producteurs
- production OR productions
- production agricole
- production d'électricité
- produit intérieur
- produits agricoles
- produits agricoles et alimentaires
- produits de santé
- produits dérivés spéculatifs
- produits financiers
- professionnels de santé

- professions judiciaires
- professions libérales
- programmes jeunesse
- progrès social
- progressistes OR progressiste
- proportionnelle
- propriétaires
- protection animale
- protection du territoire national
- protection sociale
- protectionnisme
- prothèses auditives
- ptz
- publicité
- puissance européenne
- puissances d'argent
- pupilles de la nation
- qatar
- qualité alimentaire
- quartiers OR quartier
- quartiers populaires
- quartiers prioritaires
- quinoa
- quotas OR quota
- quotas d'importation
- quotient familial
- racines chrétiennes
- racisme OR racism OR raciste
- anti-sémite OR antisémite OR antisémites OR OR antisémitisme
- xenophobe OR xénophobe
- racisme anti-français
- radicalisation
- rationalisation
- reinformation OR réinformation OR ré-information
- réadmission
- réarmement
- recettes
- recettes publiques
- recherche agronomique
- recherche publique
- récidivistes
- reconduites à la frontière
- reconnaissance
- recours aux génériques
- rectorats
- redistributif citoyen
- redressement OR redresser
- réduction
- réduction de la déductibilité des dépenses de gros travaux en revenus fonciers
- réduction de la dépense
- réduction des déficits
- réduction du taux d'endettement public OR réduction de la dette publique
- réduction du temps de travail
- réduire de moitié l'immigration
- réduire le nombre de parlementaires
- réduire les surcoûts
- référendum
- réforme
- réforme de la dépendance
- réforme de la fiscalité
- réfugiés
- régalien
- régime dérogatoire
- régime fiscal
- régime général
- régime social des indépendants
- régions OR région
- réglementation européenne
- règles du détachement
- regroupement familial
- régularisations
- régulariser des clandestins
- réhabilitation des logements
- réindustrialisation OR réindustralisation OR ré-industrialisation
- religion OR religions OR religieux
- relocalisation
- rembourser OR remboursements OR remboursable OR remboursement
- remises automatiques de peine
- remises de peine
- remplacement OR remplacer
- rémunération du président de la république
- rémunération forfaitaire
- rémunérations OR rémunération
- renégociation
- renouvelables OR renouvelable
- renouvellement urbain
- rénovation
- rénovation thermique
- rénovation urbaine
- renseignement intérieurs
- rentrées fiscales OR recettes fiscales
- représentants de l'état
- représentants des salariés
- reprise d'entreprises
- république
- république française OR république françaises
- réseaux sociaux
- réserve parlementaire
- réservée aux migrants clandestins
- réservistes
- résidences principales
- résistance OR résistances
- responsabilité pénale
- responsable politiques OR responsable politique OR responsables politiques
- ressources naturelles
- restaurants d'entreprise
- restauration collective OR restaurations collectives
- restauration scolaire
- rétention administrative

- retenue à la source
- retraite OR retraités OR retraites OR retrait
- retraite solidaire
- revalorisation OR revaloriser
- revenir à l'équilibre de nos comptes publics
- revenu universel
- revenus de l'assistance
- revenus du capital
- revenus modestes
- révisions constitutionnelles
- révolution citoyenne
- révolution fiscale
- révolution numérique
- rgpp OR révision générale des politiques publiques
- richesse nationale
- robots OR robotisation
- route migratoire
- royaume-uni
- rsa
- rsi
- ruralité
- russes OR russe
- russie
- rythmes scolaires
- sacrifices OR sacrifice
- salafisme OR salafiste OR salafistes
- salaire des fonctionnaires OR point d'indice
- salaire horaire
- salaire minimum
- salaire moyen
- salaires OR salaire
- salarié OR salariés OR salariées OR salarié.e.s
- sanctions
- santé
- santé publique
- savoirs fondamentaux
- schengen
- sciences OR science
- scolarité
- scolarité obligatoire
- scop
- secret de l'instruction
- secteur amateur
- secteur bancaire
- secteur privé
- secteur public
- secteurs régaliens
- secteurs stratégiques
- sécurité
- sécurité collective
- sécurité des français
- sécurité intérieure
- sécurité nationale
- sécurité sociale
- seniors OR senior OR séniors
- sens de l'etat
- séparation des pouvoirs
- service civique
- service militaire
- service national
- service public OR services publics
- service sanitaire
- services de l'état
- services innovants
- services publics marchands
- simplification OR simplifier
- situation irrégulière
- small business act
- smart nation
- smic
- smicards
- sncf
- snowden
- société civile
- société du travail
- soins d'urgence
- soins dispensés
- solaire
- soldats
- solidarité OR solidarités OR solidaire OR solidaires
- sols
- solutions de garde
- sommetelevage
- sondage OR sondages
- sortir du nucléaire
- souffrance au travail
- souffrances OR souffrance
- sources d'énergie
- sous-traitants
- souveraineté
- souverainetés démocratiques
- souverainetés nationales OR souveraineté nationale
- spectacle vivant
- spéculation OR spéculateurs
- sport
- sportifs amateurs
- stages OR stage
- start-ups OR startups
- statut pénal du chef de l'état
- stock options OR stock-options
- stratégie de défense
- structures parapubliques
- subprimes
- subvention publique
- successions
- suicides OR suicide
- suppression
- suppression des cotisations minimales
- suppressions de postes
- supprimant OR supprimer
- surendettement
- sûreté
- surplus
- surveillance
- suspension des allocations
- sylvicultures
- syndicat OR syndicats OR syndicaliste OR syndical OR syndicalistes OR syndicalisation
- système de santé
- système scolaire
- système universel
- tafta

- tarification progressive
- tarifs réglementés
- taux d'élucidation
- taux d'imposition
- taux d'usure
- taux de chômage
- taxe OR taxes OR taxation OR taxer OR taxerai
- taxe d'habitation
- taxe foncière
- taxe mondiale sur les transactions financières
- technologies
- technologies numériques
- télé
- téléphones portables
- temps de travail
- temps partiel
- terrains constructibles
- territoire national
- territoires OR territoire
- territoires ruraux
- terrorisme
- terrorisme islamiste
- tgv
- thales
- théorie du complot
- tiers payant
- tisa
- titres de séjour
- tolérance zéro
- totalitarisme islamique
- tourisme
- toxiques
- tpe OR très petites entreprises
- traçabilité
- traité franco-allemand
- traités
- traités européens OR traité européen
- traités internationaux
- traités transatlantiques OR traité transatlantique
- tranche supplémentaire
- transactions financières
- mariage pour tous OR mariage gay
- transactions immobilières
- manif pour tous OR manifpourtous
- transformation économique
- transition agricole
- transition écologique
- transition énergétique
- transition numérique
- transparence
- transports OR transport
- transports régionaux
- transports sanitaires
- travail
- travail clandestin
- travail détaché OR travailleur détaché
- travail obligatoires
- travailleurs
- travailleurs handicapés
- travailleurs indépendants
- très haut débit
- trésor public
- tribunal OR tribune
- tribunal administratif
- tribunal de première instance
- tribunaux correctionnels
- tribunaux pénaux
- tribunaux pénaux de proximité
- tunisie
- turquie
- tutorat
- tva
- tva à 5,5
- tva anti-délocalisations
- uber
- ultra-marins
- souverainiste
- une france puissante
- une honte
- une nouvelle société
- une république forte
- une société du travail
- uniforme à l'école
- universités OR université
- urgences sanitaires
- urssaf
- usines
- vacances
- valeurs républicaines OR valeurs de la république
- valls OR vall
- ve république
- véhicule neuf
- vente à l'étranger
- victime OR victimes
- vidéo-surveillance
- vie politique
- vieux véhicules
- village OR villages
- violencesfaitesauxfemmes OR violences faites aux femmes
- vivre-ensemble OR vivre ensemble
- voile
- voitures OR voiture
- vote utile
- votées OR votés
- washington
- web
- zone euro
- zone inondable
- zone rurale
- zones de sécurité prioritaires
- zones désertifiées
- zones prioritaires OR zone prioritaire
- zones sans réseau

J Analysis of late retweets

Several authors [3, 4] have conjectured that retweets could be negative, in particular when there are published long after the original tweet.

We studied the retweet time of all the original tweets of the accounts we follow [6]. We consider that a retweet is “late” when it takes place more than 14 days after the original tweet.

We analyzed the original tweets having at least 10% of late retweets and we kept the 100 tweets with the most retweets (109 retweets minimum). The list below shows for these 100 tweets the author name, the number of retweets, the amount of late retweets, the text of the tweet, [7], the date of the original retweet, the median date of the late retweets and finally the url of the tweet. The retweets that we categorized with certainty as negative are indicated by a red dot. The list is sorted by decreasing proportion of late retweets.

Of these 100 tweets, 30% generated negative or ironic retweets. This proportion of high negative retweets corroborates the link between negativity and retweet time. However, these seem to be limited to extreme cases of tweets having a lot of late retweets.

- ● **@Fillon2017_fr** 2848 RT (1): *"Moi, si j'étais mis en examen, je ne serais pas candidat à l'élection présidentielle. Question de morale." @FrancoisFillon #BourdinDirect* - **29/08/2016** 01/03/2017 `https://twitter.com/Fillon2017_fr/status/770150054480998400`
- ● **@bayrou** 5864 RT (1): *Emmanuel #Macron est le principal responsable de la politique économique de François #Hollande depuis quatre ans. Pour quel résultat ? #QDI* - **21/09/2016** 25/03/2017 `https://twitter.com/bayrou/status/778677784184971265`
- ● **@bayrou** 297 RT (1): *La première mesure annoncée par E. #Macron est de proposer que les jeunes travaillent plus pour gagner moins. #le79inter* - **07/12/2016** 22/02/2017 `https://twitter.com/bayrou/status/806405379605274624`
- ● **@bayrou** 1252 RT (1): *Je ne me reconnais pas ds ce qu'E. #Macron incarne. Son projet de société est proche de celui défendu par N. #Sarkozy en 2007. #QuestionsPol* - **11/09/2016** 22/02/2017 `https://twitter.com/bayrou/status/774926260266999808`
- ● **@bayrou** 116 RT (0.99): *À propos d'Emmanuel #Macron : Il ne suffit pas de dire qu'on est ni de droite ni de gauche pour être centriste #E1soir* - **22/09/2016** 22/02/2017 `https://twitter.com/bayrou/status/779008508423471104`
- ● **@BrunoLeRoux** 214 RT (0.97): *Il n'y a pas de complot, François #Fillon doit s'expliquer devant la justice #LaMatinaleInfo #cnews https://t.co/SVdDDqT2OU* - **02/03/2017** 21/03/2017 `https://twitter.com/BrunoLeRoux/status/837201126332194816`
- ● **@Fillon2017_fr** 686 RT (0.96): *"Si j'étais mis en examen je ne me présenterais pas à l'élection présidentielle. C'est évident." @FrancoisFillon #8h30Aphatie* - **02/09/2016** 01/03/2017 `https://twitter.com/Fillon2017_fr/status/771600966655234048`
- ● **@bayrou** 2782 RT (0.96): *Derrière Emmanuel #Macron il y a des gds intérêts financiers incompatibles ac l'impartialité exigée par la fonction politique #BourdinDirect* - **07/09/2016** 21/04/2017 `https://twitter.com/bayrou/status/773413492963573760`
- **@JeanLucRomero** 1046 RT (0.93): *Malgré les gesticulations de la #ManifPourTous,65% des Français pour le maintien de la loi sur le #mariagepourtous L'amour triomphe tjrs!* - **14/09/2016** 12/12/2016 `https://twitter.com/JeanLucRomero/status/776005126901424128`
- ● **@FrancoisFillon** 254 RT (0.93): *Pas d'autorité de l'Etat sans honnêteté ! Le pouvoir doit être exemplaire pour être vraiment légitime. #Fillon2017* - **21/09/2016** 01/02/2017

[6] We limit the study to these tweets because they are the only ones for which we collect the retweets, see Text B in S1 File on data collection

[7] Some emoticons had to be removed from the text

https://twitter.com/FrancoisFillon/status/778684351013789696

- **@benoithamon** 266 RT (0.92): *Triste pour @Cristiano Ronaldo et le Portugal. Énorme joueur. #PORFRA* - **10/07/2016** 22/01/2017 https://twitter.com/benoithamon/status/752222252909924353
- **@jeanlassalle** 384 RT (0.87): *Et si la France se remettait à marcher sur l'eau ? #Presidentielle2017 #Résistons https://t.co/6oKyVtBLzg* - **30/12/2016** 07/04/2017 https://twitter.com/jeanlassalle/status/814781999625936896
- **@ramayade** 207 RT (0.85): *Pour que les femmes ne soient pas représentées que par les extrêmes (Artaud, Le Pen), @ramayade, une candidature nécessaire #7novembre16h34* - **07/11/2016** 29/01/2017 https://twitter.com/ramayade/status/795654465374195713
- **@VigierPhilippe** 327 RT (0.83): *#Macron en marche aux frais des contribuables. Il ne sert pas la Fr. , il se sert. Nous allons saisir la Haute Auto... https://t.co/auOhcikwlV* - **24/01/2017** 07/03/2017 https://twitter.com/VigierPhilippe/status/823851682626990080
- **@manuelvalls** 419 RT (0.8): *La primaire est un formidable moyen pour recréer l'unité. #CeQuiNousRassemble* - **05/12/2016** 29/03/2017 https://twitter.com/manuelvalls/status/805830310155972608
- **@bayrou** 273 RT (0.78): *Je ne sais pas - à cette date - qui est E. #Macron, quels sont son projet et son positionnement, avec qui il veut gouverner. #BourdinDirect* - **01/02/2017** 22/02/2017 https://twitter.com/bayrou/status/826699689038905344
- **@FrancoisFillon** 460 RT (0.75): *Pour gouverner un pays, ma conviction est qu'il faut être irréprochable. Je pose le principe de l'exemplarité du Président et des ministres.* - **18/09/2016** 04/03/2017 https://twitter.com/FrancoisFillon/status/777431613923885056
- **@BrunoLeMaire** 625 RT (0.75): *Que les choses soient claires une bonne fois pour toutes : je ne me rallierai à AUCUN candidat au 2nd tour et je ga... https://t.co/yihPCmneMN* - **02/10/2016** 20/11/2016 https://twitter.com/BrunoLeMaire/status/782567180063813634
- **@bayrou** 532 RT (0.73): *J'ai lu avec beaucoup de soin le discours d'@EmmanuelMacron mais je n'y ai pas trouvé beaucoup de substance. #LeGrandJury* - **05/02/2017** 22/02/2017 https://twitter.com/bayrou/status/828208897261445121
- **@Bernard_Monot** 193 RT (0.72): *L'islamisme, ce cancer qui détruit tout. #Fillon, #Valls, #Sarkozy ne l'ont pas arrêté. @MLP_officiel le fera !... https://t.co/iCvsxdKUKj* - **04/01/2017** 21/03/2017 https://twitter.com/Bernard_Monot/status/816686129118375936
- **@bayrou** 213 RT (0.72): *Je ne suis pas pour que le pouvoir de l'argent prenne le pas sur la politique.#Macron #BourdinDirect @BFMTV @RMCinfo https://t.co/DittqSBegJ* - **07/09/2016** 23/02/2017 https://twitter.com/bayrou/status/773515676711399425
- **@Marion_M_Le_Pen** 3706 RT (0.68): *En avant vers la victoire ! #AssisesMLP https://t.co/4SYKqNoI01* - **04/02/2017** 23/04/2017 https://twitter.com/Marion_M_Le_Pen/status/827991739327713280
- **@EmmanuelMacron** 17669 RT (0.65): *I have a message for you guys. #ScienceMarch https://t.co/ZnkFIIksdx* - **10/02/2017** 08/05/2017 https://twitter.com/EmmanuelMacron/status/829849353867128832
- **@benoithamon** 660 RT (0.63): *Oui pour le moral j'écoute Still - Dr Dre https://t.co/oTSNCazlkJ #Punchline #Hamon2017* - **23/10/2016** 22/01/2017 https://twitter.com/benoithamon/status/790145245262544896
- **@MAZUEL_2017** 128 RT (0.62): *@KingSalman Sir, may Your Majesty pardon young #AliMohammedAlNimr. Philippe Mazuel, candidate to French Presidency https://t.co/RP3xShqLiB* - **10/09/2016** 26/11/2016 https://twitter.com/MAZUEL_2017/status/774722675361087489
- **@lepenjm** 496 RT (0.62): *Ni Droite, Ni Gauche : #Macron ! Droits d'auteur ! Mon pronostic : 3 à 4%.* - **16/11/2016** 07/05/2017 https://twitter.com/lepenjm/status/798846004556546049

- **@GLarrive** 112 RT (0.62): *La France n'a besoin, ni de la brutalité extrémiste de Marine Le Pen, ni de l'irréalité bougiste d'Emmanuel Macron... https://t.co/NlPgz1YSUB* - **23/02/2017** 10/04/2017 `https://twitter.com/GLarrive/status/834761651383566337`
- **@LydiaGuirous** 411 RT (0.61): *Retrouvez dans @Valeurs ma tribune face à l'islamisme, 2017 sera "Le quinquennat de la dernière chance" https://t.co/z1Au8CEkx9* - **09/12/2016** 08/03/2017 `https://twitter.com/LydiaGuirous/status/807204636641861632`
- ● **@FrancoisFillon** 708 RT (0.61): *Beaucoup de Français ont l'impression de travailler pour ceux qui ne travaillent pas. #PrimaireLeDebat* - **17/11/2016** 01/02/2017 `https://twitter.com/FrancoisFillon/status/799368039552708609`
- **@Simonnet2** 377 RT (0.58): *En vidéo l'arnaque de l'ubérisation, paupérisation organisée par des multinationales hors-la-loi ! La vidéo complèt... https://t.co/sEKuPuXxr4* - **23/12/2016** 04/03/2017 `https://twitter.com/Simonnet2/status/812410976989564929`
- **@ThomasScuderi** 110 RT (0.57): *#JouerCollectif pour éviter les extrèmes, tel est le défi #citoyen qui réinventera la #Politique ! Merci... https://t.co/MYeodTN1OH* - **13/11/2016** 20/12/2016 `https://twitter.com/ThomasScuderi/status/797771376094564352`
- **@marseille** 149 RT (0.57): *Suivez toute l'actualité de Marseille sur Twitter* - **20/01/2017** 09/02/2017 `https://twitter.com/marseille/status/822396311722807296`
- **@DamienObrador** 113 RT (0.57): *La #Patriosphere mobilisée pour la victoire de @MLP_officiel en 2017 ! #AvecMarine https://t.co/wmpzFFa35g* - **16/10/2016** 16/04/2017 `https://twitter.com/DamienObrador/status/787727421898391553`
- **@OuchikhKarim** 436 RT (0.54): *La République est laïque, mais la France est chrétienne. Une vérité qui s'impose à tous, y compris en politique https://t.co/jHpI0u6Izu* - **04/09/2016** 05/11/2016 `https://twitter.com/OuchikhKarim/status/772411000637317121`
- **@JLMelenchon** 349 RT (0.54): *@LiemHoangNgoc : Nous allons revaloriser les petites retraites et augmenter le minimum vieillesse. #JLMChiffrage... https://t.co/HS2gwbHopW* - **19/02/2017** 16/03/2017 `https://twitter.com/JLMelenchon/status/833320688048099328`
- ● **@FrancoisFillon** 372 RT (0.54): *Il faut une justice rapide et ferme. L'impunité zéro doit être la règle ! #PalaisDesCongrès https://t.co/puyJLiyuLj* - **18/11/2016** 01/03/2017 `https://twitter.com/FrancoisFillon/status/799690695883657216`
- **@Simonnet2** 155 RT (0.53): *Pourquoi le Gvt n'engage pas d'enquête sur Lafarge après les révélations du Monde sur sa collaboration avec #Daech ? https://t.co/q6paaQz4uP* - **06/07/2016** 13/08/2016 `https://twitter.com/Simonnet2/status/750711159737741312`
- ● **@FrancoisFillon** 133 RT (0.53): *De nous, les Français attendent transparence et intégrité : pour rétablir ordre et confiance, l'exemple doit venir d'en haut. #CNLR* - **02/07/2016** 01/02/2017 `https://twitter.com/FrancoisFillon/status/749233025184108544`
- **@PhilippePoutou** 256 RT (0.52): *Vu sur un mur : "Pour une abstention utile, votez Poutou" https://t.co/Ue3XKkODuh* - **24/11/2016** 04/04/2017 `https://twitter.com/PhilippePoutou/status/801892217803722752`
- **@EmmanuelMacron** 157 RT (0.52): *C'est un livre où j'explique la vision que j'ai du pays. #Révolution https://t.co/zwyXTULNaN* - **24/11/2016** 27/12/2016 `https://twitter.com/EmmanuelMacron/status/801850774594736128`
- **@Marion_M_Le_Pen** 663 RT (0.49): *"#Fillon est pour le droit du sol, le regroupement familial, contre la restauration des frontières, contre la prior... https://t.co/uYH3mZxDtJ* - **15/12/2016** 13/03/2017 `https://twitter.com/Marion_M_Le_Pen/status/809324258312781826`
- **@PhdeVilliers** 151 RT (0.48): *Mathieu Bock-Côté : "Un certain catholicisme se mue en utopie multiculturaliste" https://t.co/pg5sXONbU7* - **24/01/2017** 05/03/2017 `https://twitter.com/PhdeVilliers/status/823894913678974976`
- ● **@manuelvalls** 234 RT (0.46): *Le revenu universel que je défends, c'est une grande innovation sociale : garantir un revenu à tous et*

à tout momen... https://t.co/zfbbsZswUV - **27/10/2016** 24/01/2017 `https://twitter.com/manuelvalls/status/791653817544826881`

- **@lesRepublicains** 155 RT (0.46): *Trouvez le bureau de vote le plus proche de chez vous pour voter à la #Primaire2016 des 20 & 27 novembre ! https://t.co/Eb1SFDuU5e* - **13/10/2016** 09/11/2016 `https://twitter.com/lesRepublicains/status/786494987080990720`
- **@OuchikhKarim** 277 RT (0.45): *Pour #2017, je confirme ma position au #Scan : "J'appelle à voter pour #MarineLePen". "Mon coeur ne balance pas" ! https://t.co/4Qv1vfodst* - **14/10/2016** 03/11/2016 `https://twitter.com/OuchikhKarim/status/786965898540220416`
- ● **@lepenjm** 1749 RT (0.44): *Un éléphant, ça TRUMP, ça TRUMP, un éléphant ça TRUMP énormément... (l'éléphant est l'animal emblème du Parti Républicain)* - **20/07/2016** 09/11/2016 `https://twitter.com/lepenjm/status/755809512649547776`
- **@alainhoupert** 503 RT (0.43): *"La seule activité continue de l'Elysée, c'est l'hostilité à Sarkozy" Jean d'Ormesson https://t.co/U3HBfMrze7* - **02/10/2016** 14/11/2016 `https://twitter.com/alainhoupert/status/782483601225113600`
- **@PhdeVilliers** 150 RT (0.41): *Alexandre Devecchio : "Derrière l'affaire Théo, les banlieues en sécession" https://t.co/mPmz4ZXqre* - **14/02/2017** 27/03/2017 `https://twitter.com/PhdeVilliers/status/831510972661641216`
- **@Paris** 118 RT (0.41): *.@RElementaires organise une collecte de tampons et serviettes pour la #JourneeInternationaleDesDroitsDesFemmes ... https://t.co/Mje4mQdWg3* - **08/03/2017** 25/03/2017 `https://twitter.com/Paris/status/839509879316443137`
- **@SIEL_Off** 146 RT (0.4): *Le #SIEL apporte son soutien à #MarineLePen aux #Presidentielle2017 et aux candidats SIEL aux législatives https://t.co/ElAHoyhjzY* - **26/02/2017** 29/03/2017 `https://twitter.com/SIEL_Off/status/835950123855282176`
- ● **@FrancoisFillon** 303 RT (0.4): *Au sommet de l'Etat, l'intégrité du Président et de ses ministres doit être irréprochable car il n'y a pas d'autorité sans exemplarité.* - **18/11/2016** 31/01/2017 `https://twitter.com/FrancoisFillon/status/799696415555551232`
- **@Paris** 552 RT (0.39): *Parc #RivesDeSeine, venez admirer Paris au fil de l'eau https://t.co/9gfz5Uy1ln https://t.co/8e5jQFSFL1* - **22/03/2017** 20/04/2017 `https://twitter.com/Paris/status/844513445823303680`
- **@EmmanuelMacron** 1680 RT (0.39): *Non, nous ne devons pas accepter qu'en Tchétchénie des camps pour homosexuels soient réouverts. #MacronPrésident https://t.co/wriwoHdvoc* - **17/04/2017** 02/05/2017 `https://twitter.com/EmmanuelMacron/status/854021146924240896`
- ● **@DLF_Officiel** 113 RT (0.39): *.@dupontaignan "Je suis un gaulliste social. Chez DLF, il n'y a pas d'arrière boutique d'extrême droite comme au #FN" #LEntretienPolitique* - **15/03/2017** 29/04/2017 `https://twitter.com/DLF_Officiel/status/842096496417898503`
- **@alainjuppe** 163 RT (0.39): *Je crois à l'esprit Bordelais. Une ville fidèle à son esprit girondin, à la tempérance, à la modération. Tout ne vient pas d'en haut.* - **12/01/2017** 06/02/2017 `https://twitter.com/alainjuppe/status/819500637083881473`
- ● **@FrancoisFillon** 146 RT (0.37): *Un délit : une sanction ! Voilà la règle qui doit être parfaitement claire pour les délinquants. #PalaisDesCongrès* - **18/11/2016** 31/01/2017 `https://twitter.com/FrancoisFillon/status/799690849311490048`
- **@OuchikhKarim** 206 RT (0.36): *L'#islam est incompatible avec la #République : il doit être mis sous tutelle ! Ma tribune sur @BVoltaire https://t.co/LvvLYDoclJ* - **16/11/2016** 10/12/2016 `https://twitter.com/OuchikhKarim/status/798787380496863232`
- ● **@FrancoisFillon** 196 RT (0.36): *Pour être Président de la République, il faut être soi-même irréprochable. #BourdinDirect* - **18/11/2016** 29/01/2017 `https://twitter.com/FrancoisFillon/status/799519082538209280`

- **@UPR_Asselineau** 482 RT (0.35): *Frédéric Robert, maire de Monêtier-Allemont : "Non Mr @EmmanuelMacron, La commune, La France ne sont pas à vendre !... https://t.co/DmQTXqqPDN* - **26/03/2017** 04/05/2017 `https://twitter.com/UPR_Asselineau/status/846108489479000064`
- **@FrancoisFillon** 249 RT (0.35): *Nous devons entendre les Français. Nous devons entendre leur demande d'honnêteté, de courage et de vérité. #CNlesRépublicains* - **14/01/2017** 01/02/2017 `https://twitter.com/FrancoisFillon/status/820233545046171648`
- **@FrancoisFillon** 152 RT (0.35): *C'est le cri de désespoir des Français lassés de travailler pour ceux qui ne travaillent pas qui a guidé la rédaction de mon programme.* - **03/09/2016** 25/01/2017 `https://twitter.com/FrancoisFillon/status/772024442377670656`
- **@dupontaignan** 534 RT (0.35): *Retrouvez mon clip officiel de campagne pour la #présidentielle A découvrir et à partager ! #NDA2017 #DeboutLaFrance https://t.co/sfffjfbkJc* - **28/03/2017** 20/04/2017 `https://twitter.com/dupontaignan/status/846759051505651712`
- **@RafikSmati** 155 RT (0.33): *Ce que cache vraiment la candidature Macron... https://t.co/RIwz4VnirU* - **15/01/2017** 13/02/2017 `https://twitter.com/RafikSmati/status/820611357456404480`
- **@LydiaGuirous** 166 RT (0.33): *Ce n'est pas à la République de s'adapter à l'islam mais à l'islam de s'adapter à la République. #Laïcité ne se négocie pas #Valls ! @leJDD* - **31/07/2016** 26/08/2016 `https://twitter.com/LydiaGuirous/status/759687863072071680`
- **@LaurencePache** 269 RT (0.33): *Merci Mr #Vidalies, pour l'ouverture à la concurrence des #TER, pour l'augmentation des péages autoroutiers, pour l... https://t.co/orfp5jUWJm* - **19/09/2016** 10/01/2017 `https://twitter.com/LaurencePache/status/777993712546811904`
- **@LGRANDGUILLAUME** 131 RT (0.32): *#taxis #VTC #loti Je serai vers 18h Gare de Lyon pour commencer la distribution pour la loi https://t.co/ZOJA9GDXJe* - **23/10/2016** 12/11/2016 `https://twitter.com/LGRANDGUILLAUME/status/790191309764038657`
- **@LellouchePierre** 109 RT (0.32): *À libre absolument dans @lemondefr, le réquisitoire d'@Anne_Hidalgo contre @EmmanuelMacron. La meilleure attaque à... https://t.co/eGjmsRsa76* - **12/01/2017** 02/05/2017 `https://twitter.com/LellouchePierre/status/819611464168308739`
- **@JeanLucRomero** 1248 RT (0.32): *Tweetons tous cette campagne de prévention #VIH que fustigent tous les homophobes et bigots d'un autre temps !... https://t.co/vho592qDfv* - **18/11/2016** 17/01/2017 `https://twitter.com/JeanLucRomero/status/799681071214428160`
- **@FrancoisFillon** 130 RT (0.3): *Il n'y a pas d'autorité sans prestige, sans une certaine dignité et une intégrité certaine. #PalaisDesCongres* - **18/11/2016** 01/02/2017 `https://twitter.com/FrancoisFillon/status/799684116941676544`
- **@benoithamon** 372 RT (0.3): *Saisissons la chance de ne pas subir le dérèglement climatique,les mutations de la société,de l'industrie, mais d'e... https://t.co/omCWDIiNrA* - **19/03/2017** 04/04/2017 `https://twitter.com/benoithamon/status/843493085770334208`
- **@pierredeniziot** 399 RT (0.29): *Ce moment gênant où tu vas sur le site de #Macron et où il n'y a pas d'onglet #projet... https://t.co/mHx0WJVxSn* - **21/01/2017** 05/02/2017 `https://twitter.com/pierredeniziot/status/822908822608023552`
- **@PhdeVilliers** 776 RT (0.29): *Philippe de Villiers pense à s'afficher aux côtés de Marine Le Pen https://t.co/xUd3iiVfdF* - **27/03/2017** 29/04/2017 `https://twitter.com/PhdeVilliers/status/846416458594832384`
- **@LellouchePierre** 125 RT (0.29): *Je le dis avec gravité, la question de l'application de l'art. 68 de la Constitution (destitution pour manquement à ses devoirs) est posée* - **19/10/2016** 07/11/2016 `https://twitter.com/LellouchePierre/status/788754826712719361`
- **@UPR_Asselineau** 158 RT (0.27): *ÉVÉNEMENT : Grand rassemblement de l'UPR le 25 mars à Paris ! Réservez votre place sur notre site ! #Asselineau2017*

https://t.co/hHAEPnOfyt - **17/02/2017** 05/03/2017 `https://twitter.com/UPR_Asselineau/status/832525210729451520`

- **@Marion_M_Le_Pen** 771 RT (0.27): *#LesRépublicains : "Plutôt que de vous contenter d'un plan B, tournez-vous vers le plan M, le plan Marine" #Fillon https://t.co/r3rV34j7fB* - **06/02/2017** 06/03/2017 `https://twitter.com/Marion_M_Le_Pen/status/828571665315532801`
- ● **@Fillon2017_fr** 434 RT (0.27): *"Je veux une classe politique exemplaire, avec des ministres qui ne sont pas mis en examen." @FrancoisFillon #AvecFillonElevonsLeDébat* - **24/11/2016** 01/03/2017 `https://twitter.com/Fillon2017_fr/status/801882003536805890`
- **@najatvb** 137 RT (0.26): *Avec ce livre, "La vie a plus d'imagination que toi", j'ai voulu parler de la France telle que je la connais, telle... https://t.co/zLStE8DUCN* - **31/03/2017** 30/04/2017 `https://twitter.com/najatvb/status/847764078609260544`
- **@JLMelenchon** 919 RT (0.26): *#Juppé et #Fillon ont parlé de l'#ISF et des riches. Mais pas un mot sur les 9 millions de pauvres en France.... https://t.co/0pJbZHRpvx* - **24/11/2016** 01/04/2017 `https://twitter.com/JLMelenchon/status/801905686296657920`
- **@DidierTauzin** 174 RT (0.26): *Exigeons un casier judiciaire vierge à nos hommes politiques ! Ils doivent être exemplaires. https://t.co/VrvvAui83D* - **05/11/2016** 11/12/2016 `https://twitter.com/DidierTauzin/status/794954967840854020`
- **@valerieboyer13** 163 RT (0.25): *Porter le voile est perçu comme un signe d'allégeance à notre ennemi. Je proposerai un texte pour y mettre un terme,luttons c/ le salafisme* - **11/09/2016** 22/11/2016 `https://twitter.com/valerieboyer13/status/774908438417670144`
- **@NicolasSarkozy** 3203 RT (0.25): *Je souhaite le meilleur pour mon pays et pour celui qui aura à conduire la France que j'aime tant - NS https://t.co/xNYy61YUVV* - **20/11/2016** 30/03/2017 `https://twitter.com/NicolasSarkozy/status/800451750045970432`
- **@MLP_officiel** 248 RT (0.25): *"L'immense majorité des homosexuels ne demandent pas le mariage gay. Ils ne réclament qu'un PACS amélioré." #BFMPolitique* - **16/10/2016** 28/04/2017 `https://twitter.com/MLP_officiel/status/787604569593356289`
- **@MLP_officiel** 648 RT (0.24): *"On doit renvoyer les clandestins chez eux et maîtriser nos frontières nationales, sinon les camps se reconstituero... https://t.co/HEZ64kypQn* - **24/01/2017** 25/03/2017 `https://twitter.com/MLP_officiel/status/823916093064548355`
- **@MLP_officiel** 611 RT (0.24): *"Nous vivons la fin d'un monde et la naissance d'un autre ! C'est le retour des États-Nations !" #Koblenz https://t.co/IHH2wFSMz4* - **21/01/2017** 20/03/2017 `https://twitter.com/MLP_officiel/status/822762303925981184`
- ● **@manuelvalls** 1483 RT (0.24): *Je suis candidat à la présidence de la République. MV* - **05/12/2016** 29/01/2017 `https://twitter.com/manuelvalls/status/805827675482951680`
- **@LydiaGuirous** 562 RT (0.24): *Message aux "féministes" démissionnaires de gauche qui défendent #burkini voile burqa...au nom d'une fausse Liberté https://t.co/ku378P1PNf* - **07/09/2016** 20/10/2016 `https://twitter.com/LydiaGuirous/status/773586425342222337`
- **@Simonnet2** 281 RT (0.23): *Il y a pas écrit La Poste, là ! Ne laissons pas les supérettes faire main basse sur la ville, et sur le service pub... https://t.co/9AJZgojfI4* - **20/11/2016** 16/12/2016 `https://twitter.com/Simonnet2/status/800321460078252033`
- **@MLP_officiel** 447 RT (0.23): *"Les pompiers et les policiers sont immobilisés chaque jour pour régler les problèmes liés aux jungles de #migrants... https://t.co/ck0qUBXXhG* - **24/01/2017** 26/03/2017 `https://twitter.com/MLP_officiel/status/823916442613612544`
- **@GilbertCollard** 223 RT (0.23): *Et vous demandez comment on peut faire des économies : https://t.co/5UtaUJDxLV* - **06/01/2017** 22/03/2017 `https://twitter.com/GilbertCollard/status/817302224040853504`
- **@ECiotti** 1013 RT (0.23): *Je propose à @EliseLucet d'enquêter sur le financement de la*

campagne de M. #Macron,elle qui a enquêté sur M.... https://t.co/ABahs9ombb - **06/02/2017** 11/03/2017
https://twitter.com/ECiotti/status/828542977815494656

- **@valerieboyer13** 245 RT (0.21): *L'assassinat de #ZhangChaolin passe sous silence ... ! https://t.co/BbGwDjK86w* - **15/08/2016** 05/09/2016 https://twitter.com/valerieboyer13/status/765314139971526657
- **@valerieboyer13** 184 RT (0.21): *Jamais nous ne laisserons prendre en otage ni la démocratie ni le destin de notre pays. Soutien à @FrancoisFillon https://t.co/VCbOpTfO6Q* - **02/02/2017** 01/03/2017 https://twitter.com/valerieboyer13/status/827165016684130304
- **@Paris** 198 RT (0.21): *#ParisPlages revient du 20/07 au 04/09. Le programme complet https://t.co/hQoJzM430L https://t.co/60ZVJhVPT2* - **19/07/2016** 17/08/2016 https://twitter.com/Paris/status/755342759968530432
- **@OuchikhKarim** 308 RT (0.21): *Qui peut croire un seul instant que #Macron soit capable de garantir aux Français une majorité pour gouverner ! https://t.co/nr4cDS3HpA* - **09/03/2017** 27/03/2017 https://twitter.com/OuchikhKarim/status/839875214079057922
- **@MLP_officiel** 476 RT (0.21): *"Il n'y a rien pour nous de plus beau que la France, il n'y a rien pour nous de plus grand que la France !"... https://t.co/1jeyJvAeka* - **05/02/2017** 21/03/2017 https://twitter.com/MLP_officiel/status/828249040450101250
- **@GilAverous** 335 RT (0.21): *Si on me demande pourquoi je soutiens @NicolasSarkozy, la réponse est là ↓ #ToutPourLaFrance https://t.co/u2lSs9ohE9* - **16/09/2016** 15/10/2016 https://twitter.com/GilAverous/status/776765712232747008
- **@francoisbaroin** 163 RT (0.21): *Rassemblement total autour de @FrancoisFillon pour la présidentielle. 2/2* - **27/11/2016** 31/01/2017 https://twitter.com/francoisbaroin/status/802974514191728641
- **@alainjuppe** 668 RT (0.21): *Je me sens plus proche du Pape François que de Sens Commun et de la #ManifPourTous #JuppéPrésidentCest sur @France2tv #20H* - **21/11/2016** 16/04/2017 https://twitter.com/alainjuppe/status/800781567090130944
- **@OuchikhKarim** 560 RT (0.2): *#Macron est aussi l'homme qui a vendu #Alstom aux Américains. La France bradée, c'est lui ! https://t.co/B3H50PsoFH https://t.co/6nAy6ipJte* - **05/02/2017** 05/04/2017 https://twitter.com/OuchikhKarim/status/828193741504651264
- **@Marion_M_Le_Pen** 2499 RT (0.2): *"Tous les musulmans ne sont pas djihadistes, mais tous les djihadistes sont musulmans."#BBRGrandSud https://t.co/Kq7TfXxIJW* - **09/07/2016** 29/10/2016 https://twitter.com/Marion_M_Le_Pen/status/751776519538745344

References

1. Chavalarias D, Panahi M, Gaumont N. Politoscope data : tweets Ids for the 2017 French Presidential Elections; 2018. Available from: `doi:10.7910/DVN/6739SP`.

2. Colleoni E, Rozza A, Arvidsson A. Echo chamber or public sphere? Predicting political orientation and measuring political homophily in Twitter using big data. Journal of Communication. 2014;64(2):317–332.

3. Metaxas P, Mustafaraj E, Wong K, Zeng L, O'Keefe M, Finn S. What Do Retweets Indicate? Results from User Survey and Meta-Review of Research. In: Ninth International AAAI Conference on Web and Social Media; 2015. p. 658–661.

4. Guerra PC, Souza RCSNP, Assunção RM, Meira W. Antagonism also Flows through Retweets: The Impact of Out-of-Context Quotes in Opinion Polarization Analysis. arXiv preprint arXiv:170303895. 2017;.

K List of figures and tables

List of Figures

A **Description of the capture protocol centred on political leaders.** This is complemented by a keyword-centric capture protocol (Twitter track API). . . 9

B **Statistics on retweets capture volumes** *(a) Up:* Evolution of retweet capture rate over time. Some low points correspond to server failures. *(b) Bottom:* Daily number of tweets collected. 10

C **Synthetic description of the Multivac infrastructure used to set up the real-time analysis of the tweets.** This infrastructure leverages technologies such as Elasticsearch, Apache Hadoop, RabbitMQ, MongoDB and Redis. 12

D **Distribution of bot probability.** Blue curve: accounts that have been at least once in a community while having at least 3 links in the retweet graph. Orange curve: random accounts that have been at least once in a community. This detection was carried out in June 2016. 15

E **Twitter activity patterns.** *On the left:* Number of retweets observed per day and hour between 1 July 2016 and 2 May 2016; *on the right*: Cumulative distribution of retweet time. 17

F C**umulative distribution of time spent in a community between July and the 1st round.** 18

G **Smooth distribution of time spent in a community between July and the 1st round.** 18

H **Tweet propagation patterns.** *(a) left:* boxplot of the propagation entropy of each tweet within the communities, and within the entire network ; *(b) right:* a tweet from François Fillon "*A Rexecode study has shown that my program would allow 1.5 million jobs to be created. This is the program that we should implement.*" (779 retweets, entropy=0.245) and its preferred propagation within certain communities (the reference map is Fig. 4 of the paper). 20

I **Evolution of the topics discussed by the community François Asselineau between June 01, 2016 and May 08, 2017.** 25

J **Evolution of the topics discussed by the community François Fillon between June 01, 2016 and May 08, 2017.** 25

K **Evolution of the topics discussed by the community Benoit Hamon between June 01, 2016 and May 08, 2017.** 26

L **Evolution of the topics discussed by the community Marine Le Pen between June 01, 2016 and May 08, 2017.** 26

M **Evolution of the topics discussed by the community Emmanuel Macron between June 01, 2016 and May 08, 2017.** 27

N **Evolution of the topics discussed by the community Jean-Luc Mélenchon between June 01, 2016 and May 08, 2017.** 27

O **Monthly usage of the 15 most specific terms in the Macron community for the topic *employment*.** 28

P **Monthly usage of the 15 most specific terms in the Le Pen community for the topic *employment*.** 28

Q **Monthly usage of the 15 most specific terms in the Fillon community for the topic *employment*.** . . . 28
R **Monthly usage of the 15 most specific terms in the Mélenchon community for the topic *employment*.** . . . 29
S **Monthly usage of the 15 most specific terms in the Hamon community for the topic *employment*.** . . . 29
T **Monthly usage of the 15 most specific terms in the Asselineau community for the topic *employment*.** . . . 29

List of Tables

A **Bot presence in communities.** Average number of accounts per week and per community ($< accounts >$) as well as the average number of bots detected per week per community($< bots >$). . . . 15
B **Proportion of tweets posted by bots among political communities and by bots among randomly selected accounts.** For example, bots in political communities emitted 7.2% of the original tweets issued by all accounts being in a political community. . . . 16
C **Accounts statistics per community**. Table showing, for each candidate, the number of accounts present at least once in his/her community inside the community over the period July 1st 2016 - May 23th 2017. The communities of Le Pen and Fillon are clearly different to the others, in the sense that they are the most stable with a very high average time. The fact that the average time spent by an account within a community (last line) is very high indicates that a significant proportion of accounts change from one community to another, without disappearing from Twitter. . . . 17
D **Entropy of $3-$communities discussing each theme.** The 2 main communities according to the $TF - IDF$ score are also displayed for each theme. The maximum entropy is 2.585 when the 6 communities have the same volume of tweets on this theme. . . . 24
